# Brain-derived neurotrophic factor gene variants and obesity in former smokers

**DOI:** 10.1186/s12864-021-07928-0

**Published:** 2021-09-15

**Authors:** Shan-shan Yang, Yao He, Lin Xu, Yali Jin, Wei-sen Zhang, Chao-qiang Jiang, Kar Keung Cheng, Tai Hing Lam

**Affiliations:** 1 Institute of geriatrics, the 2nd Medical Center,Beijing Key Laboratory of Aging and Geriatrics, National Clinical Research Center for Geriatrics Diseases, Chinese PLA General Hospital, 28 Fuxing Road, Beijing, 100853 China; 2 Department of Disease Prevention and Control, the 1st Medical Center, Chinese PLA General Hospital, 28 Fuxing Road, Beijing, 100853 China; 3 School of Public Health, Sun Yat-sen University, Guangzhou, Guangdong China; 4 School of Public Health, The University of Hong Kong, Hongkong, China; 5 Guangzhou Number 12 People’s Hospital, Guangzhou, China; 6 Institute of Applied Health Research, University of Birmingham, Edgbaston, Birmingham, UK

**Keywords:** Obesity, Central obesity, Smoking cessation, *BDNF*

## Abstract

**Objective:**

From genome-wide association studies, brain-derived neurotrophic factor (*BDNF*) locus on chromosome 11 was the only SNP associated with both smoking and body mass index (BMI) in European, African and Asian population. This study aims to explore the unique genetic predisposition to obesity in former smokers by examining the effects of BDNF on BMI and waist circumference (WC).

**Methods:**

The study design is case-control study with a cohort validation in supplementary. We included 15,072 ethnic Chinese participants in the Guangzhou Biobank Cohort Study (GBCS) with data of four *BDNF* SNPs related to both BMI and smoking behavior. We used baseline smoke exposure data in 2003–2007 and follow-up outcomes of general obesity (by BMI) and central obesity (WC) in 2008–2012. Odds ratios (ORs) and 95% confidence intervals (CIs) for general obesity and central obesity associated with these SNPs were derived from logistic regression.

**Results:**

Of 15,072 participants (3169 men and 11,903 women), 1664 (11.0%) had general and 7868 (52.2%) had central obesity. In 1233 former smokers, the rs6265 GG, versus AA, genotype was associated with higher risks of general obesity (OR = 1.79, 95% CI = 1.06–3.01) and central obesity (OR = 2.08, 95% CI = 1.47–2.92) after adjustment. These associations were not significant in never or current smokers. In former heavy (≥20 cigarettes/day) smokers, the rs6265 GG genotype showed a higher odds for general obesity (OR = 2.15, 95% CI = 1.05–4.40), while no association was found in former light (1–9 cigarettes/day) smokers. Similar results were found for the association of rs6265 with central obesity and for the associations of other two *BDNF* SNPs (rs4923457 and rs11030104) with both general and central obesity.

**Conclusions:**

We firstly identified the genetic predisposition (BDNF SNPs) to general and central obesity in former smokers, particularly in former heavy smokers. The different associations of the SNPs for general/central obesity in different smoke exposure groups may be related to the competitive performance of the sites and epigenetic modification, which needs further study.

**Supplementary Information:**

The online version contains supplementary material available at 10.1186/s12864-021-07928-0.

## Introduction

Smoking is the leading preventable cause of death worldwide and large scale smoking cessation in the population is urgently needed to reduce the disease burden. However, smoking cessation is often associated with weight gain or even obesity. A large proportion (50% of female and 25% of male) of smokers do not give up smoking for this reason and some smoke to prevent obesity [1]. However, a meta-analysis of 62 studies around the world shows that 53% smokers did not have weight gain or gained less than 5Kg, and about 13% former smokers gained a body weight more than 10 kg [2]. Why some gain weight (and by varying amount) and some do not is unclear. The difference could be due to lifestyle changes, genetic factors or both. There is no study to date exploring whether weight gain attributable to smoking cessation has genetic susceptibility loci.

Previous genome-wide association studies (GWAS) identified 31 and 97 single nucleotide polymorphisms (SNPs) associated with smoking behavior [3] (a review, mostly based on GWAS conducted in European and African, only two studies based on Asian descent) and body mass index (BMI) [4] (a Genome-wide meta-analysis included association results for up to 339,224 individuals from 125 studies mostly based on European and African descent) respectively, and only brain-derived neurotrophic factor (BDNF) locus on chromosome 11 was common to both sets of GWAS. *BDNF* promotes synaptic remodeling and modulates the function of other neurotransmitters. It also plays a role in the reward response to many drugs, including nicotine [5]. Previous studies showed that BDNF played an important role in nicotine addiction and eating disorders [6, 7]. Thus, we hypothesized that SNPs on *BDNF* might be associated with weight gain/obesity related to smoking cessation. Furthermore, previous studies showed that the relationship was not consistent across all levels of BMI and waist circumference (WC), and that central obesity (defined by WC) showed stronger associations with diabetes and cardiovascular disease than general obesity (defined by BMI) [3, 8]. Even though previous GWAS of WC in European or Chinese populations did not report any significant associations from BDNF variants [9], some candidate gene studies showed a significant association of BDNF with WC in Chinese^11^ and Dutch populations^12^.

We hypothesized that SNPs on *BDNF* are the genetic factors of general or central obesity associated with smoking cessation. We used data from the Guangzhou Biobank Cohort Study (GBCS) [10–12] to analyze the associations of the BDNF SNPs with general obesity and central obesity separately in Chinese by smoking status to test the hypotheses. Firstly, we examined whether the four SNPs were associated with obesity in participants with different smoking status. Then we explored whether the genetic predisposition to obesity in former smokers was modified by the degree of tobacco exposure before smoking cessation.

## Design and methods

### Guangzhou biobank cohort study (GBCS)

Details of the methods of GBCS have been reported elsewhere [10–12]. Briefly, this is an on-going prospective cohort study including permanent Guangzhou residents aged 50 years or older that aims to examine environmental and genetic determinants of chronic diseases. The GBCS is a collaborative project among the Guangzhou 12th Hospital and the Universities of Hong Kong and Birmingham. Those who were receiving treatment for life-threatening diseases, such as cancer, or did not provide informed consent, were excluded. A total of 30,518 older Chinese individuals in Guangzhou were recruited at baseline from 2003 to 2007 and the first follow-up was conducted from 2008 to 2012. Of the 30,518 participants, 16,465 underwent genetic testing. After excluding 1393 participants with missing information, 15,072 participants with complete baseline, follow-up, and SNP data were included in the present analysis (Fig. S1).

### Measurements

Information on socioeconomic status and lifestyle, including age, sex, education, and tobacco and alcohol use, was collected using a computer-assisted standardized questionnaire administered by trained interviewers. Weight, waist circumference and standing height were measured with light indoor clothing and without shoes by trained nurses of the Guangzhou 12th Hospital.

A current smoker was defined as one who, at the time of the survey, smoked one cigarette daily for more than half a year. A former smoker was defined as a person who had smoked daily for at least 6 months during their lifetime but at the time of the survey did not use cigarettes [13, 14]. Cigarettes per day (CPD), Fagerstrom Test for Nicotine Dependence (FTND) (6 questions) and the Heavy Smoking Index (HSI) (based on 2 questions of FTND: time to the first cigarette of the day and number of cigarettes per day) were used to measure cigarette consumption and nicotine dependence in former and current smokers [15]. Furthermore, smoking exposure was measured by CPD (mild: 1–9 CPD, moderate: 10–19 CPD and heavy: ≥20 CPD) [16], and nicotine dependence was defined as FTND ≥6 and HSI ≥4 [15, 17]. BMI was calculated as weight (kg)/standing height (m)^2^. General obesity was defined as a BMI ≥ 28 kg/m^2^ using the criteria for the Asian population [18, 19]. Central obesity was based on the criteria also for the Asian population (waist circumference [WC] ≥ 90 cm in men and ≥ 80 cm in women) [20]. In this study, the smoke exposure data were identified by using the baseline data (at the recruitment in 2003–2007), and the relapsed smokers at follow up time (in 2008–2012) were identified as current smokers; the outcomes of general obesity and central obesity were identified by using the follow up data.

Educational attainment was classified as 0–6 years (primary school or less), 7–12 (middle to high school or equivalent) or ≥ 13 years (university or other tertiary education). Physical activity was measured using the short form of the International Physical Activity Questionnaire [10, 21]. We also collected the information about weight change and WC change since 18 years old; the two questions had 3 options: 1. No change, 2. Nearly the same, 3. Changed significantly.

### SNP selection

The inclusion criteria for SNP were as follows: (1) identified by GWAS or meta-analysis published before December 2018; (2) was associated with smoking behavior or BMI; (3) minor allele frequency (MAF) > 0·1 in the HapMap-CHB (Chinese Han Beijing) population; (4) detectable rate > 90% in the participants of the present study; (5) Hardy-Weinberg equilibrium (HWE) *P* > 0·001. Based on the above, we included four candidate SNPs (rs6265, rs11030104, rs4923457 and rs6484320) on *BDNF* associated with smoking behavior and BMI. Rs6265 and rs11030104 were selected from GWAS for BMI [4], and were retested for the association with BMI in our GBCS sample (Table S1–1); Rs4923457 and rs6484320 were selected from GWAS for smoking behavior in European [3, 22], and were retested for the association with CPD in our sample (Table S1–2). The four SNPs were tested for the association with WC in our sample by sex (Table S1–3).

Moreover, we tested linkage disequilibrium (LD) between the four SNPs using PLINK. As the four SNPs were in a LD region, we also conducted haplotype analysis to confirm the association between the LD region and general/central obesity in former smokers.

### Genotyping

Plasma aliquots were stored at − 80 °C. DNA was extracted from the whole peripheral blood sample using a standard proteinase K-phenol chloroform method [23]. The laboratory staff was blinded to the characteristics of the subjects, including their smoking and obesity status.

The SNPs were analyzed by a reputable commercial company in Beijing (Beijing Capital Bio Corporation) using a Mass ARRAY system (Sequenom, San Diego, CA, USA) [24]. After polymerase chain reaction (PCR) amplification, the primer extension products were analyzed using chip-based matrix-assisted laser desorption/ionization-time of flight mass spectrometry (MALDI-TOF MS) and SpectroTYPER 4·0 software, which automatically performed genotype calling using a set of digital filters optimized for the mass spectra of the oligonucleotides [25].

### Statistical analysis

HAPLOVIEW software version 4·2 (http://www.broadinstitute.org/haploview) and PLINK were used to perform the Hardy-Weinberg equilibrium (HWE) and linkage disequilibrium (LD) tests in the nested case-control study based on the cohort study (the cases were central/general obesity objectives). IBM SPSS Statistics for Windows, version 19·0 (Serial No: 5076595) was used for data analysis. The significance level for all tests was set at a two-tailed α value of 0·05. The differences in the proportions across different smoking groups were tested using Chi-square tests (Table 1). As the outcomes in this study include general obesity (defined by BMI) and central obesity (defined by WC), we also conducted Bonferroni correction and used the desired *P* value (*P* < 0.025) to be the significance level in the Tables 2 and 3. Logistic regression models were used to calculate adjusted odds ratios (aORs) of general obesity and central obesity for the SNPs in different smoke exposure groups, and by cigarette consumption or nicotine dependence before quitting in former smokers. Each genotype was analyzed as an independent group to show more information. Furthermore, *P* values for trends were calculated in the results of the additive model, and for comparison, the main results were analyzed by additive model again and shown in the supplementary tables (Table S6–1, S7–1, S8–1, S9–1, S10–1, S11–1, S22, S23). Age, sex (men/women), education (≦Primary school/ Middle school/≥College school), income level (<10000Yuan/10000-14999Yuan/≥15000Yuan, US$1 = 6.3Yuan) and physical activity (low/medium/high^11^) were included as covariates in the models. Model A was a crude model, model B was adjusted for age and sex, and model C was adjusted for age, sex, education, income and physical activity. There is such a large difference in smoking rates between men and women in China, so in this study, we first showed the main results in male and female separately in Table 2 and Table 3.

**Table 1:** Baseline characteristics (number (percentage)) of 15,072 participants

	N (%)	Smoke exposure			
	n = 15,072	Never (*n* = 12,852)	Former (*n* = 1233)	Current (*n* = 987)	*P*^§^
Sex					< 0.001
Male	3169 (21.0)	1263 (9.8)	1037 (84.1)	869 (88.0)	
Female	11,903 (79.0)	11,589 (90.2)	196 (15.9)	118 (12.0)	
Age (years)					< 0.001
50–59	7474 (49.6)	6807 (53.0)	278 (22.5)	389 (39.4)	
60–69	5794 (38.4)	4678 (36.4)	648 (52.6)	468 (47.4)	
≥ 70	1804 (12.0)	1367 (10.6)	307 (24.9)	130 (13.2)	
Education					< 0.001
≦Primary school	6025 (40.0)	5184 (40.3)	473 (38.4)	368 (37.3)	
Middle school	7762 (51.5)	6638 (51.6)	615 (49.9)	509 (51.6)	
≥ College school	1285 (8.5)	1030 (8.0)	145 (11.8)	110 (11.1)	
Income†					< 0.001
< 10000Yuan	4935 (32.7)	4356 (33.9)	307 (24.9)	272 (27.6)	
10,000-14999Yuan	6711 (44.5)	5829 (45.4)	525 (42.6)	357 (36.2)	
≥ 15000Yuan	2723 (16.4)	2106 (16.4)	337 (27.3)	280 (28.4)	
Don’t know	703 (4.7)	561 (4.4)	64 (5.2)	78 (7.9)	
Physical activity					< 0.001
Low	1237 (8.2)	1073 (8.3)	63 (5.1)	101 (10.2)	
Medium	5878 (39.0)	4937 (38.4)	502 (40.7)	439 (44.5)	
High	7957 (52.8)	6842 (53.2)	668 (54.2)	447 (45.3)	
Body Mass Index					< 0.001
< 18.5	743 (4.9)	622 (4.8)	42 (3.4)	79 (8.0)	
18.5–23.9	7360 (48.8)	6224 (48.4)	579 (47.0)	557 (56.4)	
24–27.9	5305 (35.2)	4536 (35.3)	491 (39.8)	278 (28.2)	
≥ 28	1664 (11.0)	1470 (11.4)	121 (9.8)	73 (7.4)	
Central obesity‡					< 0.001
Yes	7868 (52.2)	7090 (55.2)	492 (39.9)	286 (29.0)	
No	7204 (47.8)	5762 (44.8)	741 (60.1)	701 (71.0)	
Rs6265					0.85
AA	3988 (26.5)	3391 (26.4)	335 (27.2)	262 (26.5)	
GA	7571 (50.2)	6467 (50.3)	620 (50.3)	484 (49.0)	
GG	3513 (23.3)	2994 (23.3)	278 (22.5)	241 (24.4)	
Rs4923457					0.39
TT	3606 (23.9)	3065 (23.8)	314 (25.5)	227 (23.0)	
AT	7487 (49.7)	6402 (49.8)	606 (49.1)	479 (48.5)	
AA	3979 (26.4)	3385 (26.3)	313 (25.4)	281 (28.5)	
Rs11030104					0.44
GG	3999 (26.5)	3412 (26.5)	322 (26.1)	265 (26.8)	
AG	7584 (50.3)	6465 (50.3)	642 (52.1)	477 (48.3)	
AA	3489 (23.1)	2975 (23.1)	269 (21.8)	245 (24.8)	
Rs6484320					0.92
TT	3868 (25.7)	3291 (25.6)	325 (26.4)	252 (25.5)	
TA	7564 (50.2)	6456 (50.2)	620 (50.3)	488 (49.4)	
AA	3640 (24.2)	3105 (24.2)	288 (23.4)	247 (25.0)	

^§^P is for 3 group comparison by chi square

†US$1 = 6.3Yuan

‡Central obesity was based on the criteria for the Asian population (waist circumference (WC) ≥ 90 cm in men and ≥ 80 cm in women); General obesity was defined as a body mass index (BMI) ≥ 28 kg/m^2^ using the criteria for the Asian population

**Table 2:** Odds ratios (95%CI) of general obesity for different SNPs in different smoke exposure groups

		Never smokers				Former smokers				Current smokers		
Male	General obesity‡ n(%)	Model A	Model B	Model C	Cases	Model A	Model B	Model C	Cases	Model A	Model B	Model C
**Rs6265**												
**AA**	37 (10.4)	1	1	1	21 (7.5)	1	1	1	14 (5.9)	1	1	1
**GA**	72 (11.6)	1.13 (0.74–1.72)	1.11 (0.73–1.69)	1.11 (0.73–1.69)	39 (7.5)	1.00 (0.58–1.74)	1.02 (0.59–1.77)	1.01 (0.58–1.76)	27 (6.3)	1.07 (0.55–2.09)	1.08 (0.55–2.09)	1.07 (0.55–2.08)
**GG**	15 (5.3)	0.48 (0.26–0.90)	0.48 (0.26–0.89)	0.48 (0.26–0.89)	31 (13.0)	1.85 (1.03–3.31)	1.88 (1.05–3.37)	1.87 (1.04–3.36)	17 (8.2)	1.42 (0.68–2.95)	1.42 (0.68–2.96)	1.39 (0.67–2.91)
**P for G**		0.049	0.043	0.044		0.024**	0.03*	0.014**		0.348	0.346	0.379
**Rs4923457**												
**TT**	34 (11.3)	1	1	1	20 (7.6)	1	1	1	10 (5.0)	1	1	1
**AT**	74 (11.4)	1.01 (0.66–1.56)	0.99 (0.64–1.53)	0.99 (0.64–1.53)	37 (7.3)	0.96 (0.54–1.68)	0.97 (0.55–1.70)	0.96 (0.54–1.69)	30 (7.1)	1.44 (0.69–3.01)	1.45 (0.69–3.03)	1.44 (0.69–3.01)
**AA**	16 (5.1)	0.42 (0.23–0.78)	0.42 (0.23–0.78)	0.42 (0.23–0.78)	34 (12.6)	1.74 (1.01–3.12)	1.79 (1.00–3.20)	1.79 (1.00–3.20)	18 (7.4)	1.51 (0.68–3.36)	1.52 (0.68–3.37)	1.50 (0.67–3.33)
**P for A**		0.010**	0.019**	0.015**		0.012**	0.011**	0.009**		0.335	0.333	0.351
**Rs11030104**												
**GG**	38 (10.6)	1	1	1	19 (6.9)	1	1	1	15 (6.4)	1	1	1
**AG**	71 (11.3)	1.07 (0.70–1.62)	1.05 (0.69–1.59)	1.05 (0.69–1.6)	42 (7.9)	1.15 (0.66–2.02)	1.17 (0.67–2.06)	1.16 (0.66–2.04)	26 (6.2)	0.97 (0.50–1.87)	0.97 (0.50–1.87)	0.96 (0.50–1.85)
**AA**	15 (5.4)	0.48 (0.26–0.89)	0.48 (0.26–0.88)	0.48 (0.26–0.89)	30 (12.9)	1.99 (1.09–3.65)	2.04 (1.11–3.73)	2.04 (1.11–3.73)	17 (8.1)	1.29 (0.63–2.65)	1.29 (0.63–2.66)	1.26 (0.61–2.61)
**P for A**		0.043*	0.011**	0.039*		0.021**	0.018**	0.018**		0.487	0.483	0.530
**Rs6484320**												
**TT**	37 (10.9)	1	1	1	21 (7.7)	1	1	1	13 (5.8)	1	1	1
**TA**	71 (11.2)	1.03 (0.68–1.57)	1.02 (0.67–1.55)	1.01 (0.66–1.55)	41 (7.9)	1.03 (0.60–1.79)	1.04 (0.60–1.81)	1.04 (0.60–1.80)	29 (6.7)	1.17 (0.60–2.3)	1.17 (0.60–2.31)	1.17 (0.60–2.30)
**AA**	16 (5.5)	0.47 (0.26–0.87)	0.47 (0.25–0.86)	0.47 (0.25–0.86)	29 (11.9)	1.63 (0.90–2.93)	1.65 (0.91–2.98)	1.65 (0.91–2.97)	16 (7.5)	1.31 (0.62–2.8)	1.31 (0.62–2.8)	1.29 (0.60–2.76)
**P for A**		0.029*	0.025*	0.025*		0.099	0.091	0.092		0.483	0.482	0.516
**Female**												
**Rs6265**												
**AA**	328 (10.8)	1	1	1	7 (12.7)	1	1	1	5 (19.2)	1	1	1
**GA**	691 (11.8)	1.11 (0.96–1.27)	1.11 (0.96–1.27)	1.11 (0.96–1.27)	16 (15.8)	1.29 (0.5–3.36)	1.37 (0.52–3.62)	1.36 (0.51–3.64)	7 (12.1)	0.58 (0.16–2.02)	0.39 (0.10–1.51)	0.29 (0.07–1.27)
**GG**	327 (12.1)	1.13 (0.96–1.33)	1.13 (0.96–1.33)	1.13 (0.96–1.34)	7 (17.5)	1.46 (0.47–4.54)	1.45 (0.46–4.52)	1.47 (0.46–4.64)	3 (8.8)	0.41 (0.09–1.89)	0.32 (0.06–1.61)	0.26 (0.05–1.43)
**P for G**		0.131	0.131	0.126		0.051	0.062	0.053		0.142	0.166	0.130
**Rs4923457**												
**TT**	312 (11.3)	1	1	1	5 (9.6)	1	1	1	4 (14.8)	1	1	1
**AT**	663 (11.5)	1.02 (0.89–1.18)	1.02 (0.89–1.18)	1.03 (0.89–1.19)	17 (16.8)	1.90 (0.66–5.49)	1.94 (0.67–5.61)	1.89 (0.65–5.52)	7 (13.0)	0.86 (0.23–3.23)	0.64 (0.16–2.65)	0.62 (0.14–2.7)
**AA**	371 (12.1)	1.08 (0.92–1.27)	1.08 (0.92–1.27)	1.09 (0.93–1.28)	8 (18.6)	2.15 (0.65–7.13)	2.11 (0.63–7.02)	2.02 (0.60–6.82)	4 (10.8)	0.70 (0.16–3.08)	0.55 (0.12–2.61)	0.52 (0.10–2.60)
**P for A**		0.342	0.340	0.302		0.054	0.072	0.061		0.632	0.464	0.437
**Rs11030104**												
**GG**	330 (10.8)	1	1	1	7 (14.6)	1	1	1	5 (17.2)	1	1	1
**AG**	700 (12.0)	1.13 (0.98–1.29)	1.13 (0.98–1.29)	1.13 (0.98–1.3)	17 (15.3)	1.06 (0.41–2.75)	1.10 (0.42–2.87)	1.08 (0.41–2.84)	6 (10.9)	0.59 (0.16–2.12)	0.38 (0.09–1.53)	0.29 (0.07–1.32)
**AA**	316 (11.7)	1.10 (0.93–1.29)	1.10 (0.93–1.29)	1.10 (0.93–1.29)	6 (16.2)	1.13 (0.35–3.71)	1.13 (0.34–3.71)	1.12 (0.34–3.71)	4 (11.8)	0.64 (0.16–2.65)	0.46 (0.10–2.10)	0.40 (0.08–1.98)
**P for A**		0.258	0.257	0.254		0.836	0.835	0.852		0.537	0.340	0.296
**Rs6484320**												
**TT**	318 (10.8)	1	1	1	7 (13.7)	1	1	1	5 (17.9)	1	1	1
**TA**	700 (12.0)	1.13 (0.98–1.30)	1.13 (0.98–1.30)	1.13 (0.98–1.30)	15 (14.9)	1.10 (0.42–2.89)	1.15 (0.43–3.07)	1.19 (0.44–3.21)	6 (10.5)	0.54 (0.15–1.96)	0.35 (0.09–1.42)	0.28 (0.06–1.25)
**AA**	328 (11.7)	1.09 (0.93–1.29)	1.09 (0.93–1.29)	1.10 (0.93–1.29)	8 (18.2)	1.40 (0.46–4.22)	1.40 (0.46–4.25)	1.37 (0.45–4.20)	4 (12.1)	0.63 (0.15–2.64)	0.43 (0.09–1.98)	0.38 (0.08–1.88)
**P for A**		0.282	0.281	0.267		0.555	0.549	0.581		0.530	0.304	0.270
**Total**												
**Rs6265**												
**AA**	365 (10.8)	1	1	1	28 (8.4)	1	1	1	19 (7.3)	1	1	1
**GA**	763 (11.8)	1.11 (0.97–1.27)	1.11 (0.97–1.26)	1.11 (0.97–1.27)	55 (8.9)	1.07 (0.66–1.72)	1.10 (0.68–1.77)	1.09 (0.67–1.75)	34 (7.0)	0.97 (0.54–1.73)	0.94(0.52–1.68)	0.93 (0.52–1.67)
**GG**	342 (11.4)	1.07 (0.91–1.25)	1.07 (0.91–1.25)	1.07 (0.91–1.25)	38 (13.7)	1.74 (1.04–2.91)	1.79 (1.07–3.01)	1.79 (1.06–3.01)	20 (8.3)	1.16 (0.60–2.23)	1.11 (0.52–2.14)	1.10 (0.57–2.13)
**P for G**		0.378	0.389	0.382		0.034*	0.027*	0.027*		0.664	0.761	0.775
**Rs4923457**												
**TT**	346 (11.3)	1	1	1	25 (8.0)	1	1	1	14 (6.2)	1	1	1
**AT**	737 (11.5)	1.12 (0.89–1.17)	1.02 (0.89–1.17)	1.03 (0.90–1.18)	54 (8.9)	1.13 (0.69–1.86)	1.14 (0.70–1.88)	1.13 (0.69–1.86)	37 (7.7)	1.27 (0.67–2.41)	1.26 (0.67–2.39)	1.26 (0.67–2.39)
**AA**	387 (11.4)	1.01 (0.87–1.18)	1.01 (0.87–1.18)	1.02 (0.87–1.19)	42 (13.4)	1.79 (1.06–3.02)	1.87 (1.11–3.16)	1.87 (1.10–3.16)	22 (7.8)	1.29 (0.65–2.59)	1.26 (0.63–2.54)	1.28 (0.63–2.57)
**P for A**		0.863	0.866	0.806		0.015**	0.022**	0.015**		0.496	0.540	0.522
**Rs11030104**												
**GG**	368 (10.8)	1	1	1	26 (8.1)	1	1	1	20 (7.5)	1	1	1
**AG**	771 (11.9)	1.12 (0.98–1.28)	1.12 (0.98–1.28)	1.12 (0.98–1.28)	59 (9.2)	1.15 (0.71–1.87)	1.15 (0.71–1.87)	1.14 (0.70–1.85)	32 (6.7)	0.88 (0.49–1.57)	0.86 (0.48–1.54)	0.85 (0.47–1.52)
**AA**	331 (11.1)	1.04 (0.89–1.21)	1.03 (0.88–1.21)	1.04 (0.88–1.21)	36 (13.4)	1.76 (1.03–3.00)	1.81 (1.06–3.10)	1.81 (1.06–3.10)	21 (8.6)	1.15 (0.61–2.18)	1.10 (0.58–2.10)	1.10 (0.58–2.09)
**P for A**		0.609	0.623	0.615		0.036*	0.028*	0.028*		0.675	0.765	0.782
**Rs6484320**												
**TT**	355 (10.8)	1	1	1	28 (8.6)	1	1	1	18 (7.1)	1	1	1
**TA**	771 (11.9)	1.12 (0.98–1.28)	1.12 (0.98–1.28)	1.12 (0.98–1.28)	56 (9.0)	1.05 (0.66–1.69)	1.07 (0.66–1.72)	1.06 (0.66–1.71)	35 (7.2)	1.00 (0.56–1.81)	0.98 (0.54–1.78)	0.98 (0.54–1.78)
**AA**	344 (11.1)	1.03 (0.88–1.21)	1.03 (0.88–1.20)	1.03 (0.88–1.21)	37 (12.8)	1.56 (0.93–2.63)	1.59 (0.95–2.68)	1.56 (0.94–2.67)	20 (8.1)	1.15 (0.59–2.22)	1.11 (0.57–2.16)	1.11 (0.57–2.17)
**P for A**		0.686	0.697	0.671		0.087	0.077	0.079		0.685	0.753	0.758

‡General obesity was defined as a body mass index (BMI) ≥ 28 kg/m^2^ using the criteria for the Asian population

Model A: Crude model

Model B: Adjusted for age, sex

Model C: Adjusted for age, sex, education, income level and physical activity

*: *P* < 0.05

**: *P* < 0.025

**Table 3:** Odds ratios (95%CI) of central obesity for different SNPs in different smoke exposure groups

		Never smokers				Former smokers				Current smokers		
Male	Central obesity‡ n(%)	Model A	Model B	Model C	Cases	Model A	Model B	Model C	Cases	Model A	Model B	Model C
Rs6265												
AA	101 (28.3)	1	1	1	75 (26.8)	1	1	1	66 (28.0)	1	1	1
GA	184 (29.6)	1.07 (0.80–1.42)	1.06 (0.80–1.42)	1.06 (0.80–1.42)	173 (33.3)	1.37 (0.99–1.89)	1.37 (1.00–1.90)*	1.39 (1.01–1.92)*	97 (22.8)	0.76 (0.53–1.09)	0.76 (0.53–1.10)	0.76 (0.53–1.10)
GG	86 (30.3)	1.10 (0.78–1.55)	1.10 (0.78–1.55)	1.11 (0.79–1.56)	104 (43.7)	2.12 (1.47–3.07)**	2.13 (1.47–3.08)**	2.15 (1.49–3.12)**	56 (27.1)	0.96 (0.63–1.45)	0.96 (0.63–1.46)	0.95 (0.62–1.44)
P for G		0.575	0.579	0.553		< 0.001**	< 0.001**	< 0.001**		0.768	0.780	0.738
Rs4923457												
TT	90 (29.9)	1	1	1	74 (28.2)	1	1	1	49 (24.5)	1	1	1
AT	191 (29.4)	0.98 (0.73–1.32)	0.98 (0.72–1.32)	0.98 (0.73–1.32)	171 (33.9)	1.30 (0.94–1.80)	1.30 (0.94–1.81)	1.32 (0.95–1.83)	104 (24.5)	1.00 (0.68–1.48)	1.01 (0.68–1.49)	1.01 (0.68–1.49)
AA	90 (28.8)	0.95 (0.67–1.34)	0.95 (0.67–1.34)	0.95 (0.67–1.35)	107 (39.6)	1.67 (1.16–2.40)**	1.68 (1.17–2.41)**	1.70 (1.18–2.44)**	66 (27.0)	1.14 (0.74–1.75)	1.15 (0.75–1.77)	1.14 (0.74–1.75)
P for A		0.755	0.754	0.787		< 0.001**	0.005**	0.004**		0.517	0.500	0.530
Rs11030104												
GG	102 (28.6)	1	1	1	75 (27.4)	1	1	1	64 (27.1)	1	1	1
AG	184 (29.3)	1.03 (0.78–1.38)	1.03 (0.78–1.38)	1.03 (0.77–1.38)	175 (33.0)	1.30 (0.95–1.8)	1.31 (0.95–1.81)	1.33 (0.96–1.84)	99 (23.5)	0.82 (0.57–1.19)	0.83 (0.58–1.19)	0.83 (0.58–1.20)
AA	85 (30.7)	1.11 (0.79–1.56)	1.11 (0.78–1.56)	1.11 (0.79–1.57)	102 (44.0)	2.08 (1.44–3.02)**	2.10 (1.44–3.04)**	2.13 (1.47–3.09)**	56 (26.5)	0.97 (0.64–1.48)	0.98 (0.64–1.49)	0.97 (0.64–1.48)
P for A		0.569	0.573	0.554		< 0.001**	< 0.001**	< 0.001**		0.853	0.882	0.844
Rs6484320												
TT	95 (28.0)	1	1	1	75 (27.4)	1	1	1	64 (28.6)	1	1	1
TA	184 (29.1)	1.06 (0.79–1.41)	1.05 (0.79–1.41)	1.05 (0.78–1.41)	176 (33.9)	1.36 (0.99–1.88)	1.37 (0.99–1.88)	1.38 (1.00–1.91)*	98 (22.7)	0.74 (0.51–1.06)	0.74 (0.51–1.07)	0.74 (0.51–1.07)
AA	92 (31.5)	1.18 (0.84–1.66)	1.18 (0.84–1.66)	1.18 (0.84–1.67)	101 (41.4)	1.87 (1.30–2.71)**	1.88 (1.30–2.72)**	1.90 (1.31–2.75)**	57 (26.6)	0.91 (0.60–1.38)	0.91 (0.60–1.38)	0.90 (0.59–1.37)
P for A		0.344	0.347	0.340		0.001**	0.001**	0.001**		0.622	0.626	0.587
Female												
Rs6265												
AA	1697 (55.9)	1	1	1	36 (65.5)	1	1	1	17 (65.4)	1	1	1
GA	3408 (58.3)	1.10 (1.01–1.20)*	1.10 (1.01–1.20)*	1.10 (1.01–1.21)*	74 (73.3)	1.45 (0.71–2.94)	1.46 (0.71–2.99)	1.45 (0.70–2.99)	32 (55.2)	0.65 (0.25–1.70)	0.61 (0.23–1.63)	0.60 (0.22–1.61)
GG	1614 (59.6)	1.16 (1.05–1.29)*	1.16 (1.04–1.29)*	1.17 (1.05–1.30)*	30 (75.0)	1.58 (0.64–3.92)	1.58 (0.64–3.91)	1.62 (0.65–4.03)	18 (52.9)	0.6 (0.21–1.71)	0.57 (0.20–1.65)	0.56 (0.19–1.64)
P for G		0.005**	0.006**	0.004**		0.283	0.283	0.268		0.355	0.326	0.319
Rs4923457												
TT	1578 (57.1)	1	1	1	34 (65.4)	1	1	1	18 (66.7)	1	1	1
AT	3325 (57.8)	1.03 (0.94–1.13)	1.03 (0.94–1.13)	1.03 (0.94–1.14)	73 (72.3)	1.38 (0.67–2.83)	1.38 (0.67–2.84)	1.42 (0.68–2.93)	29 (53.7)	0.58 (0.22–1.52)	0.56 (0.21–1.47)	0.56 (0.21–1.49)
AA	1816 (59.1)	1.09 (0.98–1.21)	1.08 (0.97–1.20)	1.09 (0.98–1.21)	33 (76.7)	1.75 (0.70–4.34)	1.74 (0.70–4.33)	1.83 (0.73–4.60)	20 (54.1)	0.59 (0.21–1.65)	0.56 (0.20–1.59)	0.57 (0.20–1.64)
P for A		0.115	0.142	0.101		0.218	0.220	0.186		0.351	0.319	0.327
Rs11030104												
GG	1714 (56.1)	1	1	1	34 (70.8)	1	1	1	18 (62.1)	1	1	1
AG	3401 (58.3)	1.09 (1.00–1.19)	1.09 (1.00–1.19)	1.09 (1.00–1.20)	78 (70.3)	0.97 (0.46–2.05)	0.98 (0.46–2.06)	0.99 (0.46–2.09)	29 (52.7)	0.68 (0.27–1.71)	0.64 (0.25–1.63)	0.64 (0.25–1.66)
AA	1604 (59.5)	1.15 (1.03–1.27)*	1.14 (1.03–1.27)*	1.15 (1.03–1.28)*	28 (75.7)	1.28 (0.48–3.40)	1.28 (0.48–3.4)	1.31 (0.49–3.49)	20 (58.8)	0.87 (0.32–2.41)	0.82 (0.29–2.31)	0.85 (0.30–2.42)
P for A		0.010**	0.012**	0.01**		0.655	0.655	0.628		0.831	0.768	0.805
Rs6484320												
TT	1658 (56.2)	1	1	1	35 (68.6)	1	1	1	19 (67.9)	1	1	1
TA	3404 (58.4)	1.10 (1.00–1.20)	1.10 (1.00–1.20)	1.10 (1.00–1.20)	74 (73.3)	1.25 (0.60–2.62)	1.27 (0.60–2.66)	1.30 (0.62–2.76)	30 (52.6)	0.53 (0.20–1.36)	0.48 (0.18–1.28)	0.49 (0.18–1.30)
AA	1657 (58.9)	1.12 (1.01–1.24)*	1.12 (1.00–1.24)*	1.12 (1.01–1.25)*	31 (70.5)	1.09 (0.45–2.62)	1.09 (0.45–2.62)	1.10 (0.46–2.67)	18 (54.5)	0.57 (0.20–1.62)	0.52 (0.18–1.53)	0.52 (0.18–1.57)
P for A		0.034*	0.040*	0.033*		0.820	0.819	0.802		0.322	0.273	0.281
Total												
Rs6265												
AA	1798 (53.0)	1	1	1	111 (33.1)	1	1	1	83 (31.7)	1	1	1
GA	3592 (55.5)	1.11*	1.10*	1.10*	247 (39.8)	1.34*	1.39*	1.39*	129 (26.7)	0.78	0.75	0.75
		(1.02–1.20)	(1.01–1.20)	(1.01–1.20)		(1.01–1.77)	(1.03–1.86)	(1.04–1.87)		(0.56–1.09)	(0.54–1.06)	(0.54–1.06)
GG	1700 (56.8)	1.16**	1.15**	1.16**	134 (48.2)	1.88**	2.06**	2.08**	74 (30.7)	0.96	0.90	0.89
		(1.05–1.29)	(1.04–1.28)	(1.05–1.28)		(1.35–2.61)	(1.46–2.90)	(1.47–2.92)		(0.66–1.39)	(0.61–1.32)	(0.60–1.32)
P for G		0.002**	0.005**	0.004**		< 0.001**	< 0.001**	< 0.001**		0.774	0.547	0.524
Rs4923457												
TT	1668 (54.4)	1	1	1	108 (34.4)	1	1	1	67 (29.5)	1	1	1
AT	3576 (54.9)	1.02	1.02	1.03	244 (40.3)	1.29	1.32	1.33	133 (27.8)	0.92	0.93	0.93
		(0.94–1.11)	(0.94–1.12)	(0.94–1.13)		(0.97–1.71)	(0.98–1.77)	(0.99–1.79)		(0.65–1.30)	(0.65–1.33)	(0.65–1.33)
AA	1906 (56.3)	1.01	1.07	1.08	140 (44.7)	1.54**	1.69**	1.70**	86 (30.6)	1.05	1.04	1.04
		(0.87–1.18)	(0.97–1.18)	(0.98–1.19)		(1.12–2.13)	(1.21–2.37)	(1.22–2.39)		(0.72–1.54)	(0.70–1.55)	(0.70–1.54)
P for A		0.124	0.185	0.137		0.008**	0.002**	0.002**		0.742	0.793	0.814
Rs11030104												
GG	1816 (53.2)	1	1	1	109 (33.9)	1	1	1	82 (30.9)	1	1	1
AG	3585 (55.5)	1.09*	1.09*	1.09*	253 (39.4)	1.27	1.25	1.26	128 (26.8)	0.82	0.81	0.81
		(1.01–1.19)	(1.00–1.18)	(1.00–1.19)		(0.96–1.68)	(0.93–1.68)	(0.94–1.70)		(0.59–1.14)	(0.58–1.14)	(0.58–1.14)
AA	1689 (56.8)	1.15**	1.14**	1.15**	130 (48.3)	1.83**	1.96** (1.39–2.78)	1.99**	76 (31.0)	1.00	0.97	0.96
		(1.05–1.27)	(1.03–1.26)	(1.04–1.27)		(1.31–2.55)		(1.41–2.81)		(0.69–1.46)	(0.66–1.42)	(0.65–1.42)
P for A		0.004**	0.009**	0.008**		< 0.001**	< 0.001**	< 0.001**		0.984	0.838	0.815
Rs6484320												
TT	1753 (53.3)	1	1	1	110 (33.8)	1	1	1	83 (32.9)	1	1	1
TA	3588 (55.6)	1.10*	1.09*	1.09	250 (40.3)	1.32*	1.35*	1.36*	128 (26.2)	0.72	0.71	0.71
		(1.01–1.19)	(1.00–1.19)	(1.00–1.19)		(1.00–1.75)	(1.00–1.80)	(1.01–1.82)		(0.52–1.01)	(0.51–1.00)	(0.51–1.00)
AA	1749 (56.3)	1.13**	1.12**	1.13**	132 (45.8)	1.65**	1.74**	1.75**	75 (30.4)	0.89	0.86	0.85
		(1.03–1.25)	(1.02–1.24)	(1.02–1.25)		(1.19–2.29)	(1.24–2.45)	(1.25–2.47)		(0.61–1.30)	(0.58–1.26)	(0.58–1.26)
P for A		0.013**	0.024**	0.019**		0.002**	0.001**	0.001**		0.518	0.418	0.397

‡Central obesity was based on the criteria for the Asian population (waist circumference [WC] ≥ 90 cm in men and ≥ 80 cm in women)

Model A: Crude model

Model B: Adjusted for age, sex

Model C: Adjusted for age, sex, education, income level and physical activity

*: *P* < 0.05

**: *P* < 0.025

For sensitivity and in-depth analysis, we excluded participants with BMI < 18·5 kg/m^2^; divided former smokers by duration of smoking cessation (whether or not ≥5 years) and repeated the analysis. Due to the cultural differences in China, the prevalence of smoking in Chinese women is low. Results of a nationwide representative survey in 2011 (China Health and Nutrition Survey) showed smoking prevalence of 51.6 and 2.9% in men and women, respectively [26]. Because most former and current smokers were men, we repeated the analysis including men only. Further, Cox regression models were used to calculate adjusted relative risk (aRRs) of new general obesity and new central obesity found during follow up for the SNPs in different smoke exposure groups.

### Ethical considerations

The study was performed in accordance with the ethical standards of the Declaration of Helsinki (1964) and its subsequent amendments. The GBCS was approved by the Medical Ethics Committee of the Chinese PLA General Hospital. All participants provided written informed consent before participating.

## Results

### Characteristics

A total of 15,072 participants (11,903 females and 3169 males) were included. At baseline, the average age (± SD) was 62.1 ± 15.4 years. Based on Asia criteria, 6969 participants were classified as overweight, 1664 as generally obese, and 7868 as centrally obese at follow up. There were 12,852 never smokers, 1233 former smokers, and 987 current smokers. These three smoke exposure groups differed in terms of sex, age, education, income, physical activity, central obesity and BMI (*P* < 0·01) but had similar distributions of rs6265, rs4923457, rs11030104, and rs6484320 genotypes (*P* > 0·05) (Table 1). In former smokers, the median (IQR) was 33.5 (17.5) years for smoking duration and 12.0 (14.0) years for the length of smoking cessation. Table S2 shows significant interactions between BDNF SNPs and smoking status on general obesity (defined by BMI) and central obesity (defined by WC) (P for interaction from < 0.001 to 0.005). However, the association with weight gain≥1 kg and WC increase ≥5 cm was not statistically significant. Results of other SNPs (rs4923457, rs11030104 and rs6484320) were similar in former smokers, but not in never smokers and current smokers.

### Primary outcomes: association of SNPs with obesity by smoking status

Table 2 shows that in male former smokers, compared to those with the AA rs6265 genotype, the GG genotype had a higher risk of general obesity (aOR = 1.87, 95% CI = 1.04–3.36) after adjusting for age, education, income and physical activity. Compared with the TT genotype of rs4923457, the aOR of AA was 1.79 (95%CI = 1.00–3.20; similarly, compared to the GG genotype of rs11030104, the aOR of AA was 2.04 (95%CI = 1.11–3.73). In male never smokers, compared to those with the AA rs6265 genotype, the GG genotype had a lower risk of general obesity (aOR = 0.48, 95% CI = 0.26–0.89) after adjustment. Compared with the TT genotype of rs4923457, the aOR of AA was 0.42 (95%CI = 0.23–0.78; compared to the GG genotype of rs11030104, the aOR of AA was 0.48 (95%CI = 0.26–0.89). In current smokers and female, no association was observed for the 3 SNPs above (Table 2). Table S22 showed the additive model results. In the former smokers of the whole sample, the results were similar as in male former smokers, and no significant association was found in never and current smokers of the whole sample.

Table 3 shows that the results for central obesity were similar to general obesity in former smokers. The aORs for GG genotype of rs6265, AA of rs4923457, AA of rs11030104 and AA of rs6484320 in male former smokers were 2.15 (95% CI = 1.49–3.12), 1.70 (95% CI = 1.18–2.44), 2.10 (95% CI = 1.44–3.04) and 1.88 (95% CI = 1.30–2.72), respectively. No associations were found in male never and current smokers. In female never smokers, the ones with GG genotype of rs6265, AA of rs4923457, AA of rs11030104 and AA of rs6484320 had lower risk to be central obesity, and no significant associations were found in female former and current smokers (Table 3). The aOR for GG genotype of rs6265, AA of rs4923457, AA of rs11030104 and AA of rs6484320 was 2.08 (95% CI = 1.47–2.92), 1.70 (95% CI = 1.22–2.39), 1.99 (95% CI = 1.41–2.81) and 1.75 (95% CI = 1.25–2.47), respectively in the whole sample. Table S23 showed the additive model results. These associations were also observed in never smokers, but not in current smokers.

The four SNPs located on *BDNF* exhibited linkage disequilibrium. Table S3 shows that in former smokers, when generally obese former smokers were considered as cases, the AGAA haplotype of the four SNPs showed a higher risk of general obesity compared with that of TATG (aOR = 1.40, 95% CI = 1.05–1.85). Table S4 shows that in former smokers, compared to the TATG haplotype of the four SNPs, those with AGAA had a higher risk of central obesity (aOR = 1.32, 95% CI = 1.11–1.57).

### Secondary outcomes: the modification effects of smoking exposure and nicotine dependence before smoking cessation on the association between the SNPs and obesity in former smokers

**Fig. 1:**
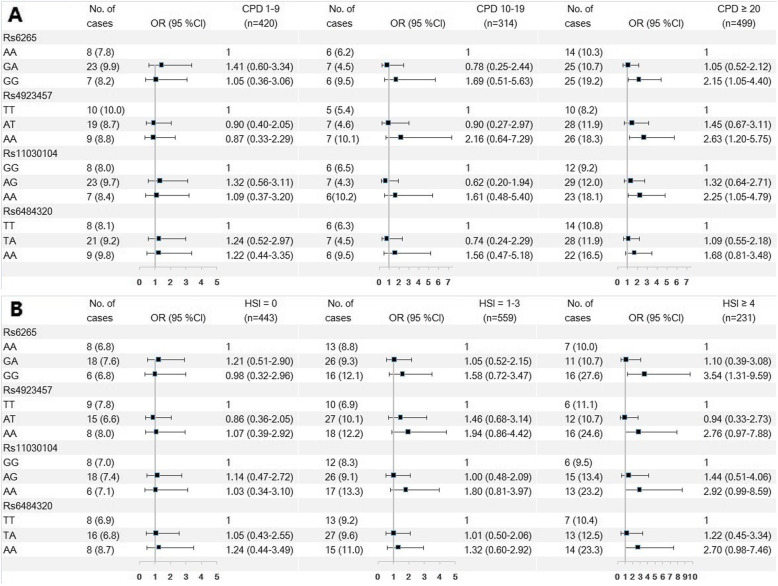
Association of SNPs with general obesity in former smokers by different smoke exposure before cessation. **A**: Cigarettes per day [CPD] 0–9 before cessation (left) vs. CPD 10–19 group (middle) vs. heavy smokers (CPD ≥ 20) before cessation (right); **B**: Heavy Smoking Index (HSI) with 0 score group (left) vs. HSI 1–3 score group (middle) vs. HSI ≥ 4 before cessation (right)

Former smokers with heavier consumption or nicotine dependence (in groups of CPD ≥ 20, HSI ≥ 4 and FTND ≥6 or by CPD, HSI and FTND as continuous variables) before smoking cessation had higher risk of general and central obesity (Table S5). Moreover, in former smokers before smoking cessation, heavy smokers (CPD ≥ 20) with a GG rs6265 genotype had a higher risk of general obesity (aOR = 2.15, 95%CI = 1.05–4.40), while the risk of the GG genotype in CPD 1–9 group was 1.05 (95%CI = 0.36–3.06), and in CPD 10–19 group was 1.69 (95%CI = 0.51–5.63) after adjustment, P for trend of CPD groups in GG rs6265 genotype =0.006. Similar dose-response associations were observed in AA rs4923457 and AA rs11030104 genotype. (Fig. 1A, Table S6, S6–1). Also, in former smokers before smoking cessation, in HSI ≥ 4 group, those with a GG rs6265 genotype had a higher risk of general obesity than AA genotype (aOR = 3.54, 95%CI = 1.31–9.59), while the risk of the GG genotype in HSI = 0 group was 0.98 (95%CI = 0.32–2.96) and in HSI 1–3 group was 1.58 (95%CI = 0.72–3.47) after adjustment, P for trend of HSI score groups in GG rs6265 genotype < 0.001 (Fig. 1B, Table S7, S7–1). Similar results were observed for FTND score groups (Table S8, S8–1).

**Fig. 2:**
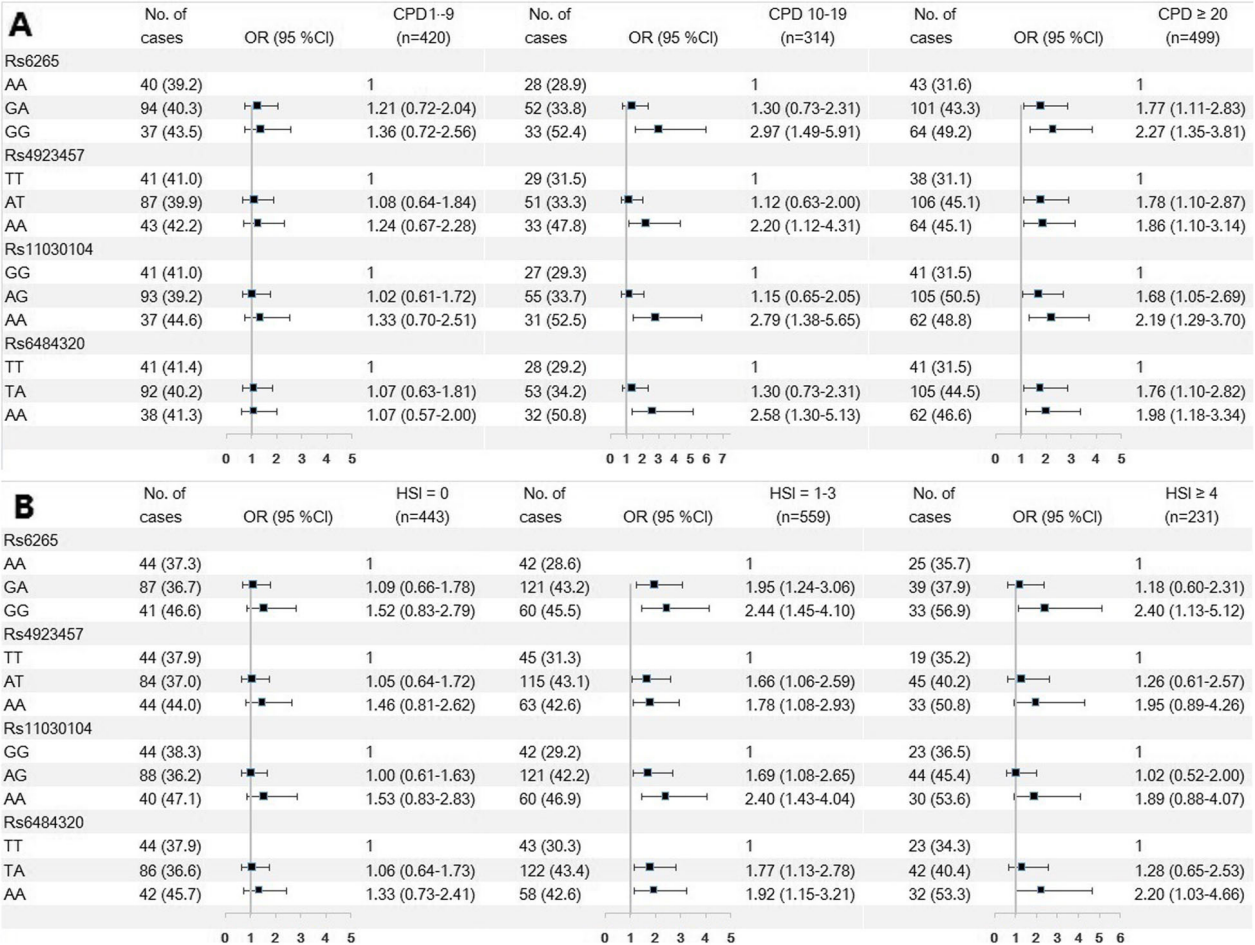
Association of SNPs with central obesity in former smokers by different smoke exposure before cessation. **A**: Cigarettes per day [CPD] 0–9 before cessation (left) vs. CPD 10–19 group (middle) vs. heavy smokers (CPD ≥ 20) before cessation (right); **B**: Heavy Smoking Index (HSI) with 0 score group (left) vs. HSI 1–3 score group (middle) vs. HSI ≥ 4 before cessation (right)

The results were similar for central obesity (Fig. 2). In heavy smokers before smoking cessation, the aOR for GG genotype of rs6265 (compared with AA) was 2.27 (95%CI = 1.35–3.81), and was 1.36 (95%CI = 0.72–2.56) in CPD 1–9 group and 2.97 (95%CI = 1.49–5.91) in CPD 10–19 group. Similar associations were observed in AA rs4923457, AA rs11030104 and AA rs6484320 genotype (Fig. 2A, Table S9, S9–1) and for HSI score and FTND score groups (Fig. 2B and Table S10-S11, S10–1 and S11–1).

### Sensitivity and in-depth analysis

Furthermore, we conducted sensitivity analyses excluding participants with BMI < 18.5 kg/m^2^. The associations of rs6265, rs4923457 and rs11030104 with general obesity in former smokers remained with similar aORs (Table S12). We also examined whether the effects of the SNPs on general and central obesity varied by duration of smoking cessation (≤ 5 years and > 5 years) and found that the AA rs4923457 genotype was significantly associated with general obesity in those with smoking cessation > 5 years only (Table S13). Similar associations of the GG rs6265, AA rs4923457, AA rs11030104 and AA rs6484320 genotypes with central obesity in those with smoking cessation ≤5 years, or > 5 years were found (Table S15).

Furthermore, we excluded participants with general (or central) obesity at baseline and assessed the association with the newly developed general (or central) obesity during follow-up. Because of small sample size, even though the associations between the genotypes of the four SNPs and general obesity were not statistically significant, the patterns were similar (Table S14). Both GG rs6265 and AA rs11030104 genotype remained significantly associated with new central obesity in former smokers (Table S16). Since individuals without weight change after the age of 18 were also unlikely to gain weight because of smoking cessation, we conducted sensitivity analyses excluding participants without weight change since 18 years old and only found significant associations of rs6265, rs4923457 and rs11030104 with general obesity in the former smokers (Table S17). We included participants whose WC changed significantly since 18 years old only and repeated the analysis as in Table 3 and in males only (Table S18 and S19). The associations of rs6265, rs4923457, rs11030104 and rs6484320 with central obesity were similar and only significant in former smoking men (Table S19). After excluding former smokers who had quitted smoking because of diseases, similar aORs for general obesity and central obesity were found (*n* = 746, Table S20).

## Discussion

This study showed that three of the four SNPs on *BDNF* were associated with general obesity and central obesity in former smokers but not in never and current smokers, which suggests genetic predisposition for obesity associated with smoking cessation. Furthermore, our LD analysis to verify the association of each base combination on general and central obesity showed that the association of SNPs on *BDNF* in former smokers with general and central obesity varied by cigarette consumption and nicotine dependence before smoking cessation.

Even though there has been no previous study that explored the genetic susceptibility loci of obesity related with smoking cessation, previous studies have tested the association between SNPs and BMI by different status of smoke exposure. We searched and found only two related studies. Megan D Fesinmeyer, et al. (25) used Population Architecture using Genomics and Epidemiology (PAGE) data (16,750 African Americans (AA) and 39,716 European Americans (EA)) and tested 10 SNPs selected by genome-wide association studies (GWAS) for BMI or obesity in different smoking exposure groups. However, they combined former smokers and never smokers as one group, so they did not report genetic susceptibility loci of obesity related with smoking cessation. Further, they only adjusted age as a confounder and stratified by racial/ethnic group and sex. They found that rs6548238/TMEM18 C genotype was associated with BMI in AA female nonsmokers (former and never smokers combined) only, and for rs9939609/FTO, the association between A allele and BMI was stronger in current EA female smokers (β = 0·017, *p* = 3·5 × 10^− 5^) than that in former/never female smokers (β = 0·006, *p* = 0·05). They concluded that there was limited evidence that smoking status may modify genetic effects of previously identified genetic risk factors for BMI [27]. Another study of European subjects used the 15q25 SNP-rs1051730 as an instrumental variable and tested the relationship between smoking exposure and BMI. They found that T of rs1051730 showed a lower BMI (by 0·23 kg/m^2)^ in ever smokers (and the association was significantly stronger in current smokers than former smokers), but no association was observed in never smokers. They concluded that exposure to smoking was causally associated with lower BMI but did not observe attenuation of the associations between genotype and BMI upon adjustment for smoking quantity in the former and current smokers [28]. To our knowledge, our study is the first to show that three SNPs on *BDNF* were associated with general obesity as well as central obesity in former smokers only, which may indicate a unique genetic mechanism for obesity related to smoking cessation.

Furthermore, the effect varied by cigarette consumption and nicotine dependence before smoking cessation. This phenomenon that the amount smoked before smoking cessation was an important factor that contributed to the magnitude of long term weight gain following smoking cessation as reported in a US population-based cohort study which did not show SNP results [29]. Our study showed that this phenomenon could be due to the effect of SNP genotype on *BDNF*. The results of our study suggest future research directions on the genetic mechanism for this phenomenon.

*BDNF* is an important member of the neurotrophin (NT) family, mainly expressed in the hippocampus and cortex. Previous GWAS studies found that SNPs in *BDNF* were related to tobacco addiction and BMI [4]. This may indicate that smoking behavior and overeating could be two kinds of addictive behavior triggered by *BDNF*. In former smokers, this addictive behavior indicated by cigarette consumption and nicotine dependence before smoking cessation may be expressed as compensatory overeating which leads to obesity after stopping smoking. There is also evidence that *BDNF* plays a role in regulation of appetite and result in a phenotype of hyperphagia and obesity [30]. Further studies are needed to clarify the mechanism, and *BDNF* may be the effective intervention targets of severe obesity after smoking cessation.

Our study had several limitations. First, the most suitable study design to examine the question would have been a prospective study following up a sufficient number of new quitters to examine their subsequent weight gain related to genotypes. However, this is not feasible as all participants of our study were older Chinese and the number of recent quitters was small. We did not have information on changes in BMI/WC in former smokers since smoking cessation. However, when we excluded participants with about the same weight or WC since the age of 18 (i.e. excluding those with weight changed significantly) and former smokers who quit smoking because of disease respectively, the results were similar (Table S19, S21, S22, S23). Furthermore, prospective analyses of the newly developed general and central obesity showed similar results, with GG rs6265 and AA rs11030104 genotype being significantly associated with new central obesity in former smokers (Table S17 and Table S19). Such results can strengthen the evidence for causality. However, in this study, lack of external replication cohort might be another limitation, which warrants further research. Second, we did not include a measure of energy intake and therefore could not comment on the role of energy intake. Third, although the GBCS is representative of the population aged 50 years or older in Guangzhou, participants in the present sample with genotype data included more women, were on average younger, and more physically active than those without genotype data. However, the BMI of these two groups did not differ (Table S21). Fourth, the number of former and current smokers in our population was relatively small, which may lead to less precise resluts. Fifth, after searching the EBI GWAS catalog on 2021/5/20, it is found that 8 new SNPs (Table S24) were associated with both BMI and smoking behavior in GWAS studies (the research was based on the European population), However, due to the lack of the detection data, this study did not analyze the eight SNPs. Lastly, our results did not account for local ancestry. However, as our participants were all ethnic Chinese in Guangzhou, and 91.56% was Han Nationality [31, 32], the confounding effects due to ancestry, if any, might not be substantial.

In conclusion, three of the four SNPs on *BDNF* had an association with general and central obesity in former smokers only, particularly those with heavy cigarette consumption or nicotine dependence before abstinence. These SNPs may be effective intervention targets to prevent obesity after smoking cessation. Our results suggest that heavy smokers with such genetic markers could be at higher risk of obesity after quitting and health care professionals helping them to quit need to pay special attention to this problem.

## Supplementary Information

**Additional file 1 Table S1–1** The relationship between rs6265, rs11030104 and body mass index (BMI) in the total study sample (*N* = 15,072). **Table S1–2** The relationship between rs4923457, rs6484320 and heavy smoking (cigarettes per day ≥ 20) in smokers (*N* = 2220). **Table S1–3** The relationship between the four SNPs and waist circumference by sex in the total study sample (female = 11,903, male = 3169). **Table S2** Interaction between different SNPs and smoke exposure group. **Table S3** Linkage disequilibrium (LD) analysis of the four SNPs’ association with general obesity‡ in former smokers. **Table S4** Linkage disequilibrium (LD) analysis of the four SNPs’ association with central obesity‡ in former smokers. **Table S5** Association of different smoke exposure groups before cessation with general obesity and central obesity (grouped and continuous variables) in former smokers. **Table S6** Association of SNPs with general obesity in different cigarettes per day (CPD) groups before cessation in former smokers. **Table S6–1** Association of SNPs with general obesity in different cigarettes per day (CPD) groups before cessation in former smokers (additive model). **Table S7** Association of SNPs with general obesity in different Heavy Smoking Index (HSI) score groups before cessation in former smokers. **Table S7–1** Association of SNPs with general obesity in different Heavy Smoking Index (HSI) score groups before cessation in former smokers (additive model). **Table S8** Association of SNPs with general obesity in different Fagerstrom Test for Nicotine Dependence (FTND) score groups before cessation in former smokers. **Table S8–1** Association of SNPs with general obesity in different Fagerstrom Test for Nicotine Dependence (FTND) score groups before cessation in former smokers (additive model). **Table S9** Association of SNPs with central obesity in different cigarettes per day (CPD) groups before cessation in former smokers. **Table S9–1** Association of SNPs with central obesity in different cigarettes per day (CPD) groups before cessation in former smokers (additive model). **Table S10** Association of SNPs with central obesity in different Heavy Smoking Index (HSI) score groups before cessation in former smokers. **Table S10–1** Association of SNPs with central obesity in different Heavy Smoking Index (HSI) score groups before cessation in former smokers (additive model). **Table S11** Association of SNPs with central obesity in different Fagerstrom Test for Nicotine Dependence (FTND) score groups before cessation in former smokers. **Table S11–1** Association of SNPs with central obesity in different Fagerstrom Test for Nicotine Dependence (FTND) score groups before cessation in former smokers (additive model). **Table S12** Odds ratios (ORs) (95%CI) of general obesity for different SNPs in different smoke exposure groups (excluding participants with body mass index (BMI) < 18.5 (*N* = 14,329). **Table S13** Odds ratios (ORs) (95%CI) of general obesity for different SNPs in former smokers (grouped by years of smoking cessation). **Table S14** Relative risks (RRs) (95%CI) of new general obesity for different SNPs in former smokers. **Table S15** Odds raios (ORs) (95%CI) of central obesity for different SNPs in former smokers (grouped by years of smoking cessation). **Table S16** Relative risks (RRs) (95%CI) of new central obesity for different SNPs in former smokers. **Table S17** Odds Ratios (ORs) (95%CI) of general obesity‡ for different SNPs in different smoke exposure groups (including participants whose weight changed significantly since 18 years old only, *N* = 11,891). **Table S18** Odds ratios (ORs) (95%CI) of central obesity‡ for different SNPs in different smoke exposure groups (including participants whose waist circumference changed significantly since 18 years old only, *N* = 11,485). **Table S19** Odds ratios (ORs) (95%CI) of central obesity‡ for different SNPs in different smoke exposure groups (including male participants whose waist circumference changed significantly since 18 years old only, *N* = 2299). **Table S20** Odds ratios (ORs) (95%CI) of general obesity and central obesity‡ for different SNPs in former smokers (excluding participants who quit smoking because of diseases, *N* = 746). **Table S21** Characteristics of the participants with or without genetic data. **Table S22** Odds ratios (95%CI) of general obesity for different SNPs in different smoke exposure groups (additive model). **Table S23** Odds ratios (95%CI) of central obesity for different SNPs in different smoke exposure groups (additive model). **S24** The new 8 SNPs both related with smoking behavior and BMI in EBI GWAS Catalog.

**Additional file 2: Fig. S1** The flow chart of participants.

## Data Availability

Data are available upon reasonable request from Dr. Yao He; mail: yhe301@x263.net

## References

[1] Spring B, Howe D, Berendsen M, McFadden HG, Hitchcock K, Rademaker AW, Hitsman B (2009). Behavioral intervention to promote smoking cessation and prevent weight gain: a systematic review and meta-analysis. Addiction.

[2] Aubin HJ, Farley A, Lycett D, Lahmek P, Aveyard P (2012). Weight gain in smokers after quitting cigarettes: meta-analysis. Bmj.

[3] Shanshan Yang YW, Liu M, Wu L, Wang J, He Y (2013). Research progress in genome-wide association of smoking behavior. Chin J Epidemiol.

[4] Locke AE, Kahali B, Berndt SI, Justice AE, Pers TH, Day FR, Powell C, Vedantam S, Buchkovich ML, Yang J (2015). Genetic studies of body mass index yield new insights for obesity biology. Nature.

[5] Forti LN, Njemini R, Beyer I, Eelbode E, Meeusen R, Mets T, Bautmans I (2014). Strength training reduces circulating interleukin-6 but not brain-derived neurotrophic factor in community-dwelling elderly individuals. Age.

[6] Zhen D, Liu L, Guan C, Zhao N, Tang X (2015). High prevalence of vitamin D deficiency among middle-aged and elderly individuals in northwestern China: its relationship to osteoporosis and lifestyle factors. Bone.

[7] Wong YY, Mccaul KA, Yeap BB, Hankey GJ, Flicker L (2013). Low vitamin D status is an independent predictor of increased frailty and all-cause mortality in older men: the health in men study. J Clin Endocrinol Metab.

[8] WHO. WHO report on the global tobacco epidemic, 2015: raising taxes on tobacco. Cell. 2015;48(2):261–70.

[9] Buta B, Choudhury PP, Xue QL, Chaves P, Bandeen-Roche K, Shardell M, Semba RD, Walston J, Michos ED, Appel LJ, McAdams-DeMarco M, Gross A, Yasar S, Ferrucci L, Fried LP, Kalyani RR (2017). The Association of Vitamin D Deficiency and Incident Frailty in older women: the role of Cardiometabolic diseases. J Am Geriatr Soc.

[10] Jiang C, Thomas G, Lam T, Schooling C, Zhang W, Lao X, Adab P, Liu B, Leung G, Cheng K (2006). Cohort profile: the Guangzhou biobank cohort study, a Guangzhou-Hong Kong-Birmingham collaboration. Int J Epidemiol.

[11] Yin P, Jiang CQ, Cheng KK, Lam TH, Lam KH, Miller MR, Zhang WS, Thomas GN, Adab P (2007). Passive smoking exposure and risk of COPD among adults in China: the Guangzhou biobank cohort study. Lancet (London, England).

[12] Yang S, Xu L, He Y, Jiang C, Jin Y, Cheng KK, et al. Childhood secondhand smoke exposure and pregnancy loss in never smokers: the Guangzhou biobank cohort study. Tob Control. 2016. 10.1136/tobaccocontrol-2016-053239 PMC5661265 28011924

[13] Organization WH: Guidelines for the conduct of tobacco-smoking surveys among health professionals: report of a WHO meeting held in Winnipeg, Canada, 7–9 July 1983 in collaboration with UICC and ACS. 1984.

[14] Lam KB, Jiang CQ, Jordan RE, Miller MR, Zhang WS, Cheng KK, Lam TH, Adab P (2010). Prior TB, smoking, and airflow obstruction: a cross-sectional analysis of the Guangzhou biobank cohort study. Chest.

[15] Wang TJ, Zhang F, Richards JB, Kestenbaum B, van Meurs JB, Berry D, Kiel DP, Streeten EA, Ohlsson C, Koller DL (2010). Common genetic determinants of vitamin D insufficiency: a genome-wide association study. Lancet (London, England).

[16] Shelef K, Diamond GS, Diamond GM, Myers MG. Changes in tobacco use among adolescent smokers in substance abuse treatment. Psychol Addict Behav. 23(2):355–61. 10.1037/a0014517 19586153

[17] Bayard R, Anna G, Andrew S, Kseniya K, Vladimir P, David R, Christian H, Martin M (2012). Prevalence and psychosocial determinants of nicotine dependence in nine countries of the former Soviet Union. Nicotine Tob Res.

[18] Aschner P, Beck-Nielsen H, Bennett P, Boulton A, Colagiuri R, Colagiuri S, Mcgill M, Sim K, Franz M, Gadsby R (2014). China guideline for type 2 diabetes 2010 Edn. Diab Res Clin Pract.

[19] WGOC (2004). Chinese adult overweight and obesity prevention and control guidelines (excerpts). Acta Nutrimenta Sinica.

[20] KG A, RH E, SM G, PZ Z, JI C, KA D, JC F, WP J, CM L, Jr SS. Harmonizing the metabolic syndrome: a joint interim statement of the international diabetes federation task force on epidemiology and prevention; National Heart, Lung, and Blood Institute; American Heart Association; World Heart Federation; International A. Circulation. 2009;120(16):1640–5. 10.1161/CIRCULATIONAHA.109.192644. 10.1161/CIRCULATIONAHA.109.192644 19805654

[21] Deng HB, Macfarlane DJ, Thomas GN, Lao XQ, Jiang CQ, Cheng KK, Lam TH (2008). Reliability and validity of the IPAQ-Chinese: the Guangzhou biobank cohort study. Med Sci Sports Exerc.

[22] Tobacco and Genetics Consortium. Genome-wide meta-analyses identify multiple loci associated with smoking behavior. Nat Genet. 2010;42(5):441–7. 10.1038/ng.571. Epub 2010 Apr 25. 10.1038/ng.571 PMC2914600 20418890

[23] Ho SA, Hoyle JA, Lewis FA, Secker AD, Cross D, Mapstone NP, Dixon MF, Wyatt JI, Tompkins DS, Taylor GR, et al. Direct polymerase chain reaction test for detection of Helicobacter pylori in humans and animals. J Clin Microbiol. 1991;29(11):2543–9. 10.1128/jcm.29.11.2543-2549.1991. 10.1128/jcm.29.11.2543-2549.1991 PMC270370 1723072

[24] Zhao J, Jiang C, Lam TH, Liu B, Cheng KK, Xu L, Au Yeung SL, Zhang W, Leung GM, Schooling CM (2014). Genetically predicted testosterone and cardiovascular risk factors in men: a Mendelian randomization analysis in the Guangzhou biobank cohort study. Int J Epidemiol.

[25] Ong KL, Li M, Tso AW, Xu A, Cherny SS, Sham PC, Tse HF, Lam TH, Cheung BM, Lam KS (2010). Association of genetic variants in the adiponectin gene with adiponectin level and hypertension in Hong Kong Chinese. Eur J Endocrinol.

[26] Li S, Meng L, Chiolero A, Ma C, Xi B (2016). Trends in smoking prevalence and attributable mortality in China, 1991-2011. Prev Med.

[27] Fesinmeyer MD, North KE, Unhee L, Petra B, Crawford DC, Jeffrey H, Gross MD, Fowke JH, Robert G, Shelley-Ann L (2013). Effects of smoking on the genetic risk of obesity: the population architecture using genomics and epidemiology study. BMC Med Genet.

[28] Freathy RM, Kazeem GR, Morris RW, Johnson PC, Paternoster L, Ebrahim S, Hattersley AT, Hill A, Hingorani AD, Holst C (2011). Genetic variation at CHRNA5-CHRNA3-CHRNB4 interacts with smoking status to influence body mass index. Int J Epidemiol.

[29] Veldheer S, Yingst J, Zhu J, Foulds J (2015). Ten-year weight gain in smokers who quit, smokers who continued smoking and never smokers in the United States, NHANES 2003-2012. Int J Obes.

[30] Harcourt BE, Bullen DVR, Kao KT, Tassoni D, Alexander EJ, Burgess T, White SM, Sabin MA (2018). Maternal inheritance of BDNF deletion, with phenotype of obesity and developmental delay in mother and child. Am J Med Genet A.

[31] Tabulation on the population census of the people's republic of china by county [http://www.stats.gov.cn/tjsj/pcsj/rkpc/6rp/indexch.htm].

[32] Zhang H (1988). Comprehensive analysis of finger pattern parameters in Han NATIONALITY of China. Acta Anthropologica Sinica.

