# Supplementary material for: Brain-derived neurotrophic factor gene variants and obesity in former smokers
Source: BMC Genomics. 2021 Sep 15;22:668. doi: 10.1186/s12864-021-07928-0 (PMC8442367; doi:10.1186/s12864-021-07928-0)
Supplement: Supplementary file 1 — Additional file 1 Table S1–1 The relationship between rs6265, rs11030104 and body mass index (BMI) in the total study sample (N = 15,072). Table S1–2 The relationship between rs4923457, rs6484320 and heavy smoking (cigarettes per day ≥ 20) in smokers (N = 2220). Table S1–3 The relationship between the four SNPs and waist circumference by sex in the total study sample (female = 11,903, male = 3169). Table S2 Interaction between different SNPs and smoke exposure group. Table S3 Linkage disequilibrium (LD) analysis of the four SNPs’ association with general obesity‡ in former smokers. Table S4 Linkage disequilibrium (LD) analysis of the four SNPs’ association with central obesity‡ in former smokers. Table S5 Association of different smoke exposure groups before cessation with general obesity and central obesity (grouped and continuous variables) in former smokers. Table S6 Association of SNPs with general obesity in different cigarettes per day (CPD) groups before cessation in former smokers. Table S6–1 Association of SNPs with general obesity in different cigarettes per day (CPD) groups before cessation in former smokers (additive model). Table S7 Association of SNPs with general obesity in different Heavy Smoking Index (HSI) score groups before cessation in former smokers. Table S7–1 Association of SNPs with general obesity in different Heavy Smoking Index (HSI) score groups before cessation in former smokers (additive model). Table S8 Association of SNPs with general obesity in different Fagerstrom Test for Nicotine Dependence (FTND) score groups before cessation in former smokers. Table S8–1 Association of SNPs with general obesity in different Fagerstrom Test for Nicotine Dependence (FTND) score groups before cessation in former smokers (additive model). Table S9 Association of SNPs with central obesity in different cigarettes per day (CPD) groups before cessation in former smokers. Table S9–1 Association of SNPs with central obesity in different cigarettes per da [file 12864_2021_7928_MOESM1_ESM.docx]

**Table S1-1 The relationship between rs6265, rs11030104 and body mass index (BMI) in the total study sample (N=15,072)**

|  | Model A | | | |  | Model B | | | |  | Model C | | | | |
| --- | --- | --- | --- | --- | --- | --- | --- | --- | --- | --- | --- | --- | --- | --- | --- |
|  |  | 95%CI | |  |  |  | 95%CI | |  |  |  | 95%CI | |  |  |
|  | ß | lower | upper | Standard ß | P for trend | ß | lower | upper | Standard ß | P for trend | ß | lower | upper | Standard ß | P for trend |
| rs6265 |  |  |  |  |  |  |  |  |  |  |  |  |  |  |  |
| AA (reference) | |  |  |  | <0.001 |  |  |  |  | <0.001 |  |  |  |  | <0.001 |
| GA | 0.243 | 0.108 | 0.378 | 0.034 |  | 0.240 | 0.105 | 0.374 | 0.034 |  | 0.237 | 0.102 | 0.371 | 0.034 |  |
| GG | 0.322 | 0.162 | 0.481 | 0.039 |  | 0.319 | 0.160 | 0.479 | 0.038 |  | 0.317 | 0.158 | 0.476 | 0.038 |  |
| rs11030104 | |  |  |  |  |  |  |  |  |  |  |  |  |  |  |
| GG (reference) | |  |  |  | <0.001 |  |  |  |  | <0.001 |  |  |  |  | <0.001 |
| AG | 0.245 | 0.110 | 0.380 | 0.035 |  | 0.244 | 0.109 | 0.378 | 0.035 |  | 0.243 | 0.109 | 0.377 | 0.035 |  |
| AA | 0.334 | 0.174 | 0.494 | 0.040 |  | 0.332 | 0.173 | 0.492 | 0.040 |  | 0.330 | 0.171 | 0.490 | 0.040 |  |
| Model A: Crude model Model B: Adjusted for age, sex Model C: Adjusted for age, sex, education, income level and physical activity | | | | | | | | | | | | | | | |

**Table S1-2 The relationship between rs4923457, rs6484320 and**

**heavy smoking (cigarettes per day ≥20) in smokers (N=2,220)**

|  | Cases | Model A | Model B | Model C |
| --- | --- | --- | --- | --- |
| Rs4923457 |  |  |  |  |
| TT | 225 (41.6) | 1 | 1 | 1 |
| AT | 453 (41.8) | 1.01 (0.82-1.24) | 1.00 (0.81-1.24) | 1.01 (0.81-1.25) |
| AA | 278 (46.8) | 1.24 (0.98-1.56) | 1.23 (0.97-1.57) | 1.25 (0.98-1.59) |
| P |  | 0.07 | 0.08 | 0.07 |
| Rs6484320 |  |  |  |  |
| TT | 235 (40.7) | 1 | 1 | 1 |
| TA | 471 (42.5) | 1.08 (0.88-1.32) | 1.09 (0.88-1.34) | 1.09 (0.88-1.35) |
| AA | 250 (46.7) | 1.28 (1.01-1.62) | 1.34 (1.03-1.67) | 1.31 (1.02-1.67) |
| P |  | 0.04 | 0.03 | 0.03 |
| Model A: Crude model Model B: Adjusted for age, sex Model C: Adjusted for age, sex, education, income level and physical activity | | | | |

**Table S1-3 The relationship between the four SNPs and waist circumference by sex in the total study sample (female=11,903, male=3,169)**

| Female | Model A | | | |  | Model B | | | |  | Model C | | | | |
| --- | --- | --- | --- | --- | --- | --- | --- | --- | --- | --- | --- | --- | --- | --- | --- |
|  |  | 95%CI | |  |  |  | 95%CI | |  |  |  | 95%CI | |  |  |
|  | ß | lower | upper | Standard ß | P for trend | ß | lower | upper | Standard ß | P for trend | ß | lower | upper | Standard ß | P for trend |
| rs6265 |  |  |  |  |  |  |  |  |  |  |  |  |  |  |  |
| AA (reference) | |  |  |  | 0.02 |  |  |  |  | 0.02 |  |  |  |  | 0.02 |
| GA | 0.699 | 0.111 | 1.286 | 0.026 |  | 0.695 | 0.110 | 1.279 | 0.026 |  | 0.687 | 0.103 | 1.271 | 0.025 |  |
| GG | 0.785 | 0.091 | 1.480 | 0.024 |  | 0.779 | 0.089 | 1.469 | 0.024 |  | 0.791 | 0.101 | 1.481 | 0.025 |  |
| rs11030104 | |  |  |  |  |  |  |  |  |  |  |  |  |  |  |
| GG (reference) | |  |  |  | 0.02 |  |  |  |  | 0.02 |  |  |  |  | 0.02 |
| AG | 0.737 | 0.150 | 1.324 | 0.027 |  | 0.728 | 0.144 | 1.311 | 0.027 |  | 0.724 | 0.141 | 1.307 | 0.027 |  |
| AA | 0.836 | 0.142 | 1.530 | 0.026 |  | 0.814 | 0.123 | 1.504 | 0.025 |  | 0.824 | 0.134 | 1.514 | 0.026 |  |
| rs4923457 |  |  |  |  |  |  |  |  |  |  |  |  |  |  |  |
| TT (reference) | |  |  |  | 0.11 |  |  |  |  | 0.10 |  |  |  |  | 0.10 |
| AT | 0.438 | -0.170 | 1.045 | 0.016 |  | 0.455 | -0.149 | 1.059 | 0.017 |  | 0.460 | -0.144 | 1.059 | 0.017 |  |
| AA | 0.571 | -0.118 | 1.260 | 0.019 |  | 0.579 | -0.105 | 1.264 | 0.019 |  | 0.591 | -0.105 | 1.264 | 0.019 |  |
| rs6484320 |  |  |  |  |  |  |  |  |  |  |  |  |  |  |  |
| TT (reference) | |  |  |  | 0.04 |  |  |  |  | 0.04 |  |  |  |  | 0.04 |
| AT | 0.787 | 0.193 | 1.380 | 0.029 |  | 0.769 | 0.178 | 1.358 | 0.028 |  | 0.764 | 0.174 | 1.354 | 0.028 |  |
| AA | 0.714 | 0.022 | 1.406 | 0.023 |  | 0.701 | 0.014 | 1.389 | 0.022 |  | 0.712 | 0.024 | 1.400 | 0.022 |  |

| Male | Model A | | | |  | Model B | | | |  | Model C | | | | |
| --- | --- | --- | --- | --- | --- | --- | --- | --- | --- | --- | --- | --- | --- | --- | --- |
|  |  | 95%CI | |  |  |  | 95%CI | |  |  |  | 95%CI | |  |  |
|  | ß | lower | upper | Standard ß | P for trend | ß | lower | upper | Standard ß | P for trend | ß | lower | upper | Standard ß | P for trend |
| rs6265 |  |  |  |  |  |  |  |  |  |  |  |  |  |  |  |
| AA (reference) | |  |  |  | 0.23 |  |  |  |  | 0.22 |  |  |  |  | 0.21 |
| GA | 1.053 | -0.287 | 2.394 | 0.033 |  | 1.082 | -0.258 | 2.423 | 0.033 |  | 1.098 | -0.243 | 2.439 | 0.034 |  |
| GG | 0.926 | -0.666 | 2.518 | 0.024 |  | 0.940 | -0.653 | 2.533 | 0.024 |  | 0.963 | -0.630 | 2.557 | 0.025 |  |
| rs11030104 | |  |  |  |  |  |  |  |  |  |  |  |  |  |  |
| GG (reference) | |  |  |  | 0.18 |  |  |  |  | 0.18 |  |  |  |  | 0.16 |
| AG | 1.034 | -0.307 | 2.375 | 0.032 |  | 1.066 | -0.275 | 2.408 | 0.033 |  | 1.089 | -0.253 | 2.431 | 0.034 |  |
| AA | 1.040 | -0.561 | 2.640 | 0.027 |  | 1.048 | -0.553 | 2.649 | 0.027 |  | 1.088 | -0.514 | 2.689 | 0.028 |  |
| rs4923457 |  |  |  |  |  |  |  |  |  |  |  |  |  |  |  |
| TT (reference) | |  |  |  | 0.35 |  |  |  |  | 0.35 |  |  |  |  | 0.33 |
| AT | 1.069 | -0.330 | 2.469 | 0.033 |  | 1.093 | -0.306 | 2.493 | 0.034 |  | 1.110 | -0.290 | 2.510 | 0.034 |  |
| AA | 0.794 | -0.799 | 2.387 | 0.022 |  | 0.787 | -0.807 | 2.380 | 0.021 |  | 0.818 | -0.775 | 2.412 | 0.022 |  |
| rs6484320 |  |  |  |  |  |  |  |  |  |  |  |  |  |  |  |
| TT (reference) | |  |  |  | 0.24 |  |  |  |  | 0.24 |  |  |  |  | 0.23 |
| AT | 0.949 | -0.408 | 2.305 | 0.029 |  | 0.972 | -0.385 | 2.329 | 0.030 |  | 0.979 | -0.378 | 2.336 | 0.030 |  |
| AA | 0.921 | -0.674 | 2.517 | 0.024 |  | 0.924 | -0.673 | 2.520 | 0.024 |  | 0.943 | -0.653 | 2.539 | 0.025 |  |

Model A: Crude model
Model B: Adjusted for age, sex
Model C: Adjusted for age, sex, education, income level and physical activity

**Table S2 Interaction between different SNPs and smoke exposure group**

|  | General obesity (BMI) | | | Central obesity (WC) | | |
| --- | --- | --- | --- | --- | --- | --- |
|  | Model A | Model B | Model C | Model A | Model B | Model C |
| Rs6265 by smoke exposure group | 0.91 (0.87-0.96) | 0.92 (0.87-0.96) | 0.93 (0.88-0.97) | 0.77 (0.75-0.80) | 0.76 (0.74-0.79) | 0.77 (0.75-0.79) |
| P | <0.001 | 0.001 | 0.003 | <0.001 | <0.001 | <0.001 |
| Rs4923457 by smoke exposure group | 0.92 (0.88-0.96) | 0.92 (0.88-0.97) | 0.93 (0.89-0.98) | 0.78 (0.76-0.81) | 0.77 (0.75-0.80) | 0.78 (0.75-0.80) |
| P | 0.001 | 0.001 | 0.005 | <0.001 | <0.001 | <0.001 |
| Rs11030104 by smoke exposure group | 0.91 (0.87-0.96) | 0.92 (0.87-0.97) | 0.93 (0.88-0.98) | 0.78 (0.75-0.80) | 0.76 (0.74-0.79) | 0.77 (0.75-0.79) |
| P | 0.000 | 0.001 | 0.003 | <0.001 | <0.001 | <0.001 |
| Rs6484320 by smoke exposure group | 0.91 (0.87-0.96) | 0.92 (0.87-0.96) | 0.93 (0.88-0.97) | 0.77 (0.75-0.8) | 0.76 (0.74-0.79) | 0.77 (0.74-0.79) |
| P | <0.001 | 0.001 | 0.003 | <0.001 | <0.001 | <0.001 |
| Model A: Crude model Model B: Adjusted for age Model C: Adjusted for age, education, income level and physical activity | | | | | | |

**Table S3 Linkage disequilibrium (LD) analysis of the four SNPs’ association with general obesity^‡^ in former smokers**

|  | Allele | % | X^2^ | P | OR (95%CI) | OR' (95%CI) | D' | r^2^ | LOD |
| --- | --- | --- | --- | --- | --- | --- | --- | --- | --- |
| rs4923457 | A | 50.0 | 5.358 | 0.021 | 1.33* (1.02-1.74) |  |  |  |  |
| rs6265 | G | 47.7 | 4.466 | 0.035 | 1.37* (1.05-1.79) |  |  |  |  |
| rs6484320 | A | 48.5 | 2.927 | 0.087 | 1.26 (0.97-1.65) |  |  |  |  |
| rs11030104 | A | 47.9 | 4.243 | 0.039 | 1.32* (1.01-1.73) |  |  |  |  |
| rs4923457-rs6265 | TA | 46.8 | 5.121 | 0.024 | 1 | 0.74* (0.56-0.97) | 0.865 | 0.683 | 283.62 |
|  | AG | 44.5 | 4.756 | 0.029 | 1.39* (1.05-1.84) | 1.35* (1.04-1.76) |  |  |  |
|  | AA | 5.5 | 0.105 | 0.746 | 1.26 (0.70-2.28) | 1.07 (0.60-1.89) |  |  |  |
|  | TG | 3.2 | 0.025 | 0.873 | 1.06 (0.48-2.37) | 0.89 (0.41-1.96) |  |  |  |
| rs6265-rs6484320 | AT | 50.3 | 3.040 | 0.081 | 1 | 0.79 (0.61-1.03) | 0.953 | 0.879 | 419.77 |
|  | GA | 46.5 | 4.337 | 0.037 | 1.31* (1.00-1.71) | 1.33* (1.02-1.73) |  |  |  |
|  | AA | 2.0 | 1.749 | 0.186 | 0.44 (0.11-1.84) | 0.40 (0.10-1.64) |  |  |  |
|  | GT | 1.2 | 0.025 | 0.875 | 1.20 (0.36-4.02) | 1.10 (0.33-3.68) |  |  |  |
| rs6484320-rs11030104 | TG | 49.8 | 2.910 | 0.088 | 1 | 0.79 (0.61-1.04) | 0.931 | 0.844 | 384.83 |
|  | AA | 46.1 | 4.278 | 0.039 | 1.28 (0.98-1.68) | 1.09 (0.83-1.42) |  |  |  |
|  | AG | 2.4 | 1.349 | 0.245 | 0.38 (0.09-1.57) | 0.50 (0.15-1.59) |  |  |  |
|  | TA | 1.7 | 0.000 | 0.987 | 0.82 (0.25-2.70) | 0.98 (0.34-2.73) |  |  |  |
| rs4923457-rs6484320 | TT | 46.3 | 3.370 | 0.066 | 1 | 0.78 (0.60-1.02) | 0.847 | 0.676 | 279.61 |
|  | AA | 44.8 | 4.867 | 0.027 | 1.35* (1.02-1.78) | 1.36* (1.04-1.77) |  |  |  |
|  | AT | 5.2 | 0.074 | 0.785 | 1.20 (0.65-2.21) | 1.05 (0.58-1.90) |  |  |  |
|  | TA | 3.7 | 1.636 | 0.201 | 0.61 (0.24-1.55) | 0.52 (0.21-1.31) |  |  |  |
| rs6265-rs11030104 | AG | 50.8 | 4.156 | 0.042 | 1 | 0.76* (0.58-0.99) | 0.947 | 0.891 | 423.68 |
|  | GA | 46.4 | 4.564 | 0.033 | 1.34* (1.02-1.75) | 1.34* (1.02-1.74) |  |  |  |
|  | AA | 1.5 | 0.091 | 0.763 | 0.97 (0.29-3.20) | 0.83 (0.25-2.74) |  |  |  |
|  | GG | 1.3 | 0.007 | 0.932 | 1.10 (0.33-3.66) | 0.95 (0.29-3.14) |  |  |  |
| rs4923457-rs6265-rs6484320 | TAT | 45.4 | 3.698 | 0.055 | 1 | 0.76* (0.58-1.00) |  |  |  |
|  | AGA | 44.2 | 5.164 | 0.023 | 1.37* (1.03-1.81) | 1.36* (1.04-1.78) |  |  |  |
|  | AAT | 4.9 | 0.158 | 0.691 | 1.27 (0.69-2.35) | 1.10 (0.61-1.98) |  |  |  |
|  | TGA | 2.3 | 0.353 | 0.552 | 1.01 (0.39-2.58) | 0.86 (0.34-2.17) |  |  |  |
|  | TAA | 1.4 | 1.892 | 0.169 | 0.31 (0.04-2.32) | 0.27 (0.04-1.95) |  |  |  |
| rs6265-rs6484320-rs11030104 | ATG | 49.3 | 3.219 | 0.073 | 1 | 0.78 (0.60-1.02) |  |  |  |
|  | GAA | 45.8 | 4.833 | 0.028 | 1.33* (1.01-1.74) | 1.35* (1.03-1.76) |  |  |  |
|  | AAG | 1.6 | 0.956 | 0.328 | 0.85 (0.26-2.80) | 0.49 (0.12-2.06) |  |  |  |
|  | ATA | 1.1 | 0.059 | 0.809 | 2.62* (1.05-6.54) | 1.20 (0.36-4.03) |  |  |  |
| rs4923457-rs6484320-rs11030104 | TTG | 45.0 | 3.746 | 0.053 | 1 | 0.77 (0.58-1.00) |  |  |  |
|  | AAA | 43.7 | 5.316 | 0.021 | 1.38* (1.04-1.82) | 1.37* (1.05-1.78) |  |  |  |
|  | ATG | 4.8 | 0.269 | 0.604 | 1.32 (0.72-2.44) | 1.14 (0.63-2.06) |  |  |  |
|  | TAA | 2.5 | 0.519 | 0.471 | 0.76 (0.27-2.15) | 0.65 (0.23-1.80) |  |  |  |
|  | TTA | 1.4 | 0.191 | 0.662 | 1.43 (0.49-4.13) | 1.23 (0.43-3.51) |  |  |  |
|  | TAG | 1.2 | 1.474 | 0.225 | 0.37 (0.05-2.74) | 0.31 (0.04-2.31) |  |  |  |
|  | AAG | 1.1 | 0.169 | 0.681 | 0.82 (0.19-3.52) | 0.70 (0.17-2.98) |  |  |  |
| rs4923457-rs6265-rs11030104 | TAG | 45.7 | 5.343 | 0.021 | 1 | 0.72* (0.55-0.95) |  |  |  |
|  | AGA | 43.6 | 5.276 | 0.022 | 1.41* (1.06-1.87) | 1.35* (1.03-1.76) |  |  |  |
|  | AAG | 5.1 | 0.363 | 0.547 | 1.39 (0.77-2.51) | 1.16 (0.66-2.06) |  |  |  |
|  | TGA | 2.7 | 0.205 | 0.651 | 1.07 (0.45-2.53) | 0.88 (0.38-2.06) |  |  |  |
|  | TAA | 1.1 | 0.046 | 0.830 | 1.32 (0.39-4.45) | 1.10 (0.33-3.67) |  |  |  |
| rs4923457-rs6265-rs6484320-rs11030104 | TATG | 44.4 | 4.063 | 0.044 | 1 | 0.74* (0.56-0.98) |  |  |  |
|  | AGAA | 43.4 | 5.723 | 0.017 | 1.40* (1.05-1.85) | 1.37* (1.05-1.79) |  |  |  |
|  | AATG | 4.8 | 0.238 | 0.625 | 1.42 (0.78-2.58) | 1.21 (0.68-2.15) |  |  |  |
|  | TGAA | 2.3 | 0.360 | 0.548 | 0.95 (0.37-2.42) | 0.79 (0.31-2.00) |  |  |  |
|  | TAAG | 1.2 | 1.408 | 0.236 | 0.37 (0.05-2.76) | 0.31 (0.04-2.29) |  |  |  |

‡General obesity was defined as a body mass index (BMI) ≥ 28 kg/m2 using the criteria for the Asian population.

*: P<0.05

**Table S4 Linkage disequilibrium (LD) analysis of the four SNPs’ association with central obesity^‡^ in former smokers**

|  | Allele | % | X^2^ | P | OR (95%CI) | OR' (95%CI) | D' | r^2^ | LOD |
| --- | --- | --- | --- | --- | --- | --- | --- | --- | --- |
| rs4923457 | A | 50.0 | 7.100 | 0.008 | 1.25* (1.06-1.46) |  |  |  |  |
| rs6265 | G | 47.7 | 14.185 | 0.000 | 1.36* (1.07-1.26) |  |  |  |  |
| rs6484320 | A | 48.5 | 9.151 | 0.003 | 1.28* (1.09-1.51) |  |  |  |  |
| rs11030104 | A | 47.9 | 12.039 | 0.000 | 1.33* (1.13-1.56) |  |  |  |  |
| rs4923457-rs6265 | TA | 46.8 | 8.566 | 0.003 | 1 | 0.79* (0.67-0.92) | 0.865 | 0.683 | 283.62 |
|  | AG | 44.5 | 12.444 | 0.000 | 1.34* (1.13-1.58) | 1.34* (1.14-1.58) |  |  |  |
|  | AA | 5.5 | 3.412 | 0.065 | 0.82 (0.56-1.20) | 0.71 (0.49-1.02) |  |  |  |
|  | TG | 3.2 | 0.527 | 0.468 | 1.33 (0.84-2.11) | 1.18 (0.75-1.85) |  |  |  |
| rs6265-rs6484320 | AT | 50.3 | 10.076 | 0.002 | 1 | 0.77* (0.66-0.91) | 0.953 | 0.879 | 419.77 |
|  | GA | 46.5 | 13.107 | 0.000 | 1.34* (1.13-1.57) | 1.35* (1.15-1.58) |  |  |  |
|  | AA | 2.0 | 4.477 | 0.034 | 0.57 (0.30-1.11) | 0.50* (0.26-0.96) |  |  |  |
|  | GT | 1.2 | 0.495 | 0.482 | 1.49 (0.70-3.16) | 1.31 (0.62-2.76) |  |  |  |
| rs6484320-rs11030104 | TG | 49.8 | 8.968 | 0.003 | 1 | 0.78* (0.67-0.92) | 0.931 | 0.844 | 384.83 |
|  | AA | 46.1 | 12.291 | 0.000 | 1.32* (1.12-1.56) | 1.34* (1.14-1.57) |  |  |  |
|  | AG | 2.4 | 2.421 | 0.120 | 0.71 (0.40-1.26) | 0.62 (0.35-1.09) |  |  |  |
|  | TA | 1.7 | 0.012 | 0.911 | 1.05 (0.56-1.98) | 0.93 (0.49-1.74) |  |  |  |
| rs4923457-rs6484320 | TT | 46.3 | 5.244 | 0.022 | 1 | 0.83* (0.70-0.97) | 0.847 | 0.676 | 279.61 |
|  | AA | 44.8 | 11.719 | 0.000 | 1.29* (1.09-1.53) | 1.33* (1.13-1.56) |  |  |  |
|  | AT | 5.2 | 2.787 | 0.095 | 0.82 (0.55-1.20) | 0.72 (0.50-1.06) |  |  |  |
|  | TA | 3.7 | 1.013 | 0.314 | 0.89 (0.57-1.39) | 0.80 (0.52-1.23) |  |  |  |
| rs6265-rs11030104 | AG | 50.8 | 12.732 | 0.000 | 1 | 0.75* (0.63-0.88) | 0.947 | 0.891 | 423.68 |
|  | GA | 46.4 | 13.474 | 0.000 | 1.36* (1.15-1.60) | 1.35* (1.15-1.59) |  |  |  |
|  | AA | 1.5 | 0.649 | 0.421 | 0.87 (0.43-1.76) | 0.75 (0.37-1.51) |  |  |  |
|  | GG | 1.3 | 0.197 | 0.657 | 1.36 (0.67-2.75) | 1.17 (0.58-2.37) |  |  |  |
| rs4923457-rs6265-rs6484320 | TAT | 45.4 | 6.097 | 0.014 | 1 | 0.81* (0.69-0.96) |  |  |  |
|  | AGA | 44.2 | 12.949 | 0.000 | 1.32* (1.11-1.56) | 1.36* (1.15-1.60) |  |  |  |
|  | AAT | 4.9 | 2.723 | 0.099 | 0.82(0.55-1.22) | 0.72 (0.49-1.07) |  |  |  |
|  | TGA | 2.3 | 0.015 | 0.902 | 1.14 (0.66-1.96) | 1.02 (0.60-1.74) |  |  |  |
|  | TAA | 1.4 | 3.758 | 0.053 | 0.50 (0.23-1.11) | 0.44* (0.20-0.98) |  |  |  |
| rs6265-rs6484320-rs11030104 | ATG | 49.3 | 9.355 | 0.002 | 1 | 0.78* (0.66-0.91) |  |  |  |
|  | GAA | 45.8 | 12.877 | 0.000 | 1.33* (1.13-1.57) | 1.36* (1.15-1.60) |  |  |  |
|  | AAG | 1.6 | 4.141 | 0.042 | 0.57 (0.28-1.18) | 0.50 (0.24-1.02) |  |  |  |
|  | ATA | 1.1 | 0.312 | 0.576 | 0.95 (0.44-2.08) | 0.84 (0.38-1.82) |  |  |  |
| rs4923457-rs6484320-rs11030104 | TTG | 45.0 | 5.942 | 0.015 | 1 | 0.81* (0.69-0.96) |  |  |  |
|  | AAA | 43.7 | 12.347 | 0.000 | 1.32* (1.11-1.56) | 1.33* (1.13-1.57) |  |  |  |
|  | ATG | 4.8 | 1.769 | 0.184 | 0.86 (0.58-1.29) | 0.76 (0.52-1.12) |  |  |  |
|  | TAA | 2.5 | 0.001 | 0.973 | 1.14 (0.68-1.92) | 1.02 (0.61-1.70) |  |  |  |
|  | TTA | 1.4 | 0.461 | 0.497 | 1.40 (0.70-2.82) | 1.26 (0.63-2.50) |  |  |  |
|  | TAG | 1.2 | 3.427 | 0.064 | 0.51 (0.22-1.21) | 0.45 (0.19-1.06) |  |  |  |
|  | AAG | 1.1 | 0.096 | 0.757 | 0.94 (0.43-2.05) | 0.83 (0.38-1.81) |  |  |  |
| rs4923457-rs6265-rs11030104 | TAG | 45.7 | 8.351 | 0.004 | 1 | 0.78* (0.66-0.92) |  |  |  |
|  | AGA | 43.6 | 11.861 | 0.000 | 1.34* (1.13-1.59) | 1.33* (1.13-1.57) |  |  |  |
|  | AAG | 5.1 | 2.448 | 0.118 | 0.86 (0.58-1.27) | 0.74 (0.51-1.08) |  |  |  |
|  | TGA | 2.7 | 0.567 | 0.452 | 1.36 (0.83-2.22) | 1.19 (0.73-1.94) |  |  |  |
|  | TAA | 1.1 | 0.043 | 0.836 | 1.11 (0.52-2.40) | 0.97 (0.45-2.09) |  |  |  |
| rs4923457-rs6265-rs6484320-rs11030104 | TATG | 44.4 | 6.087 | 0.014 | 1 | 0.81* (0.69-0.95) |  |  |  |
|  | AGAA | 43.4 | 12.744 | 0.000 | 1.32* (1.11-1.57) | 1.35* (1.15-1.59) |  |  |  |
|  | AATG | 4.8 | 1.994 | 0.158 | 0.85 (0.57-1.27) | 0.75 (0.51-1.11) |  |  |  |
|  | TGAA | 2.3 | 0.015 | 0.904 | 1.14 (0.66-1.96) | 1.02 (0.60-1.74) |  |  |  |
|  | TAAG | 1.2 | 3.133 | 0.077 | 0.51 (0.22-1.21) | 0.45 (0.19-1.06) |  |  |  |

‡Central obesity was based on the criteria for the Asian population (waist circumference [WC] ≥ 90 cm in men and ≥ 80 cm in women)

*: P<0.05

**Table S5 Association of different smoke exposure groups before cessation with general obesity and central obesity (grouped and continuous variables) in former smokers**

|  |  | General obesity (BMI ≥28.0) | | |  | Central Obesity (WC ≥90cm in male and WC ≥80cm in female) | | |
| --- | --- | --- | --- | --- | --- | --- | --- | --- |
|  | Cases | Model A | Model B | Model C | Cases | Model A | Model B | Model C |
| Heavy smoker |  |  |  |  |  |  |  |  |
| No | 57 (7.8) | 1 | 1 | 1 | 284 (38.7) | 1 | 1 | 1 |
| Yes | 64 (12.8) | 1.75* (1.20-2.55) | 2.15* (1.44-3.23) | 2.16* (1.44-3.24) | 208 (41.7) | 1.13 (0.90-1.43) | 1.59* (1.24-2.04) | 1.58* (1.23-2.03) |
| HIS≥4 |  |  |  |  |  |  |  |  |
| No | 87 (8.7) | 1 | 1 | 1 | 395 (39.4) | 1 | 1 | 1 |
| Yes | 34 (14.7) | 1.82* (1.19-2.78) | 2.08* (1.34-3.22) | 2.06* (1.32-3.20) | 97 (42.0) | 1.11* (0.83-1.49) | 1.42* (1.05-1.92) | 1.42* (1.05-1.92) |
| FTND≥6 |  |  |  |  |  |  |  |  |
| No | 100 (9.1) | 1 | 1 | 1 | 433 (39.5) | 1 | 1 | 1 |
| Yes | 21 (15.3) | 1.80* (1.09-3.00) | 2.01* (1.20-3.38) | 1.99* (1.18-3.36) | 59 (43.1) | 1.16 (0.81-1.66) | 1.46* (1.01-2.11) | 1.46* (1.01-2.11) |
| CPD groups |  |  |  |  |  |  |  |  |
| 1-9 | 38 (9.0) | 1 | 1 | 1 | 171 (40.7) | 1 | 1 | 1 |
| 10-19 | 19 (6.1) | 0.65 (0.37-1.15) | 0.77 (0.43-1.38) | 0.78 (0.43-1.39) | 113 (36.0) | 0.82 (0.61-1.11) | 1.09 (0.78-1.51) | 1.07 (0.77-1.48) |
| 20-29 | 39 (12.0) | 1.37 (0.86-2.20) | 1.79* (1.08-2.97) | 1.79* (1.08-2.98) | 132 (40.6) | 1.00 (0.74-1.34) | 1.57* (1.13-2.16) | 1.54* (1.11-2.13) |
| ≥30 | 25 (14.4) | 1.69 (0.98-2.89) | 2.24* (1.26-3.98) | 2.24* (1.26-3.99) | 76 (43.7) | 1.13 (0.79-1.61) | 1.83* (1.25-2.69) | 1.81* (1.24-2.66) |
| P for trend |  | 0.019 | 0.001 | 0.001 |  | 0.509 | <0.001 | <0.001 |
| HSI groups |  |  |  |  |  |  |  |  |
| 0 | 32 (7.2) | 1 | 1 | 1 | 172 (38.8) | 1 | 1 | 1 |
| 1-3 | 55 (9.8) | 1.40 (0.89-2.21) | 1.67* (1.04-2.67) | 1.66* (1.03-2.66) | 223 (39.9) | 1.05 (0.81-1.35) | 1.40* (1.06-1.85) | 1.38* (1.05-1.83) |
| ≥4 | 34 (14.7) | 2.22* (1.33-3.70) | 2.87* (1.66-4.93) | 2.84* (1.64-4.90) | 97 (42.0) | 1.14 (0.83-1.58) | 1.74* (1.23-2.47) | 1.73* (1.22-2.45) |
| P for trend |  | 0.003 | <0.001 | <0.001 |  | 0.438 | 0.001 | 0.001 |
| FTND groups |  |  |  |  |  |  |  |  |
| 0-3 | 77 (8.5) | 1 | 1 | 1 | 355 (39.2) | 1 | 1 | 1 |
| 4-5 | 23 (12.0) | 1.47 (0.90-2.41) | 1.67* (1.01-2.76) | 1.65* (1.00-2.74) | 78 (40.8) | 1.07 (0.78-1.47) | 1.65 (0.95-1.83) | 1.31 (0.94-1.83) |
| ≥6 | 21 (15.3) | 1.95* (1.16-3.28) | 2.25* (1.32-3.84) | 2.23* (1.30-3.83) | 59 (43.1) | 1.17 (0.82-1.69) | 1.54* (1.06-2.24) | 1.54* (1.06-2.24) |
| P for trend |  | 0.007 | 0.001 | 0.001 |  | 0.369 | 0.010 | 0.011 |
| HSI(continuous variable) | | 1.17 (1.06-1.29) | 1.22 (1.10-1.36) | 1.22 (1.10-1.35) |  | 1.03 (0.97-1.10) | 1.12 (1.05-1.20) | 1.12 (1.05-1.20) |
| P |  | 0.002 | <0.001 | <0.001 |  | 0.298 | 0.001 | 0.001 |
| FTND (continuous variable) | | 1.12 (1.04-1.21) | 1.16 (1.07-1.25) | 1.15 (1.07-1.25) |  | 1.03 (0.98-1.08) | 1.09 (1.04-1.15) | 1.09 (1.03-1.15) |
| P |  | 0.003 | <0.001 | <0.001 |  | 0.265 | 0.001 | 0.001 |
| BMI = body mass index, WC = waiste circumference, CPD = cigarettes per day HSI = Heavy Smoking Index FTND = Fagerstrom Test for Nicotine Dependence  Model A: Crude model Model B: Adjusted for age, sex Model C: Adjusted for age, sex, education, income level and physical activity  *P<0.05 | | | | | | | | |

**Table S6 Association of SNPs with general obesity in different cigarettes per day (CPD) groups before cessation in former smokers**

|  |  | CPD 1-9 (n=420) | | |
| --- | --- | --- | --- | --- |
|  | General obesity‡ n(%) | Model A | Model B | Model C |
| Rs6265 |  |  |  |  |
| AA | 8 (7.8) | 1 | 1 | 1 |
| GA | 23 (9.9) | 1.29 (0.56-2.98) | 1.46 (0.62-3.44) | 1.41 (0.60-3.34) |
| GG | 7 (8.2) | 1.05 (0.37-3.04) | 1.08 (0.37-3.13) | 1.05 (0.36-3.06) |
| Rs4923457 | |  |  |  |
| TT | 10 (10.0) | 1 | 1 | 1 |
| AT | 19 (8.7) | 0.86 (0.38-1.92) | 0.94 (0.41-2.11) | 0.90 (0.40-2.05) |
| AA | 9 (8.8) | 0.87 (0.34-2.24) | 0.92 (0.35-2.39) | 0.87 (0.33-2.29) |
| Rs11030104 | |  |  |  |
| GG | 8 (8.0) | 1 | 1 | 1 |
| AG | 23 (9.7) | 1.24 (0.53-2.87) | 1.35 (0.58-3.17) | 1.32 (0.56-3.11) |
| AA | 7 (8.4) | 1.06 (0.38-3.05) | 1.10 (0.38-3.21) | 1.09 (0.37-3.20) |
| Rs6484320 | |  |  |  |
| TT | 8 (8.1) | 1 | 1 | 1 |
| TA | 21 (9.2) | 1.15 (0.49-2.69) | 1.29 (0.54-3.04) | 1.24 (0.52-2.97) |
| AA | 9 (9.8) | 1.23 (0.46-3.35) | 1.26 (0.46-3.44) | 1.22 (0.44-3.35) |
|  |  | CPD 10-19 (n=314) | | |
| Rs6265 |  |  |  |  |
| AA | 6 (6.2) | 1 | 1 | 1 |
| GA | 7 (4.5) | 0.72 (0.24-2.22) | 0.72 (0.23-2.24) | 0.78 (0.25-2.44) |
| GG | 6 (9.5) | 1.60 (0.49-5.19) | 1.62 (0.49-5.34) | 1.69 (0.51-5.63) |
| Rs4923457 | |  |  |  |
| TT | 5 (5.4) | 1 | 1 | 1 |
| AT | 7 (4.6) | 0.83 (0.26-2.71) | 0.84 (0.26-2.77) | 0.90 (0.27-2.97) |
| AA | 7 (10.1) | 1.97 (0.60-6.48) | 2.06 (0.62-6.89) | 2.16 (0.64-7.29) |
| Rs11030104 | |  |  |  |
| GG | 6 (6.5) | 1 | 1 | 1 |
| AG | 7 (4.3) | 0.64 (0.21-1.98) | 0.58 (0.19-1.82) | 0.62 (0.20-1.94) |
| AA | 6(10.2) | 1.62 (0.50-5.29) | 1.58 (0.48-5.21) | 1.61 (0.48-5.40) |
| Rs6484320 | |  |  |  |
| TT | 6 (6.3) | 1 | 1 | 1 |
| TA | 7 (4.5) | 0.71 (0.23-2.18) | 0.70 (0.23-2.18) | 0.74 (0.24-2.29) |
| AA | 6 (9.5) | 1.58 (0.49-5.13) | 1.51 (0.46-4.96) | 1.56 (0.47-5.18) |
|  |  | CPD ≥ 20 (n=499) | | |
| Rs6265 |  |  |  |  |
| AA | 14 (10.3) | 1 | 1 | 1 |
| GA | 25 (10.7) | 1.05 (0.53-2.09) | 1.07 (0.53-2.14) | 1.05 (0.52-2.12) |
| GG | 25 (19.2) | 2.08* (1.03-4.20) | 2.14* (1.05-4.36) | 2.15* (1.05-4.40) |
| Rs4923457 | |  |  |  |
| TT | 10 (8.2) | 1 | 1 | 1 |
| AT | 28 (11.9) | 1.52 (0.71-3.23) | 1.46 (0.68-3.12) | 1.45 (0.67-3.11) |
| AA | 26 (18.3) | 2.51* (1.16-5.44) | 2.56* (1.17-5.59) | 2.63* (1.20-5.75) |
| Rs11030104 | |  |  |  |
| GG | 12 (9.2) | 1 | 1 | 1 |
| AG | 29 (12.0) | 1.34 (0.66-2.72) | 1.31 (0.64-2.68) | 1.32 (0.64-2.71) |
| AA | 23 (18.1) | 2.18* (1.03-4.59) | 2.22* (1.05-4.71) | 2.25* (1.05-4.79) |
| Rs6484320 | |  |  |  |
| TT | 14 (10.8) | 1 | 1 | 1 |
| TA | 28 (11.9) | 1.12 (0.52-1.98) | 1.09 (0.55-2.17) | 1.09 (0.55-2.18) |
| AA | 22 (16.5) | 1.64 (0.80-3.37) | 1.68 (0.81-3.48) | 1.68 (0.81-3.48) |
| P for trend of rs6265 GG |  | <0.001 | 0.01 | 0.01 |
| P for trend of rs4923457 AA |  | 0.03 | 0.01 | 0.01 |
| P for trend of rs11030104 AA |  | 0.04 | 0.02 | 0.02 |
| P for trend of rs6484320 AA |  | 0.12 | 0.03 | 0.04 |
| ‡General obesity was defined as a body mass index (BMI) ≥ 28 kg/m2 using the criteria for the Asian population.  Model A: Crude model Model B: Adjusted for age, sex Model C: Adjusted for age, sex, education, income level and physical activity *P<0.05 | | | | |

**Table S7 Association of SNPs with general obesity in different Heavy Smoking Index (HSI) score groups before cessation in former smokers**

|  |  | HSI=0 (n=443) | | | | |
| --- | --- | --- | --- | --- | --- | --- |
|  | General obesity‡ n(%) | Model A | Model B | | Model C | |
| Rs6265 |  |  |  | |  | |
| AA | 8 (6.8) | 1 | 1 | | 1 | |
| GA | 18 (7.6) | 1.13 (0.48-2.68) | 1.21 (0.51-2.90) | | 1.21 (0.51-2.90) | |
| GG | 6 (6.8) | 1.01 (0.34-3.01) | 0.96 (0.32-2.90) | | 0.98 (0.32-2.96) | |
| Rs4923457 |  |  |  | |  | |
| TT | 9 (7.8) | 1 | 1 | | 1 | |
| AT | 15 (6.6) | 0.84 (0.36-1.99) | 0.88 (0.37-2.08) | | 0.86 (0.36-2.05) | |
| AA | 8 (8.0) | 1.03 (0.38-2.79) | 1.04 (0.38-2.84) | | 1.07 (0.39-2.92) | |
| Rs11030104 |  |  |  | |  | |
| GG | 8 (7.0) | 1 | 1 | | 1 | |
| AG | 18 (7.4) | 1.07 (0.45-2.54) | 1.14 (0.48-2.72) | | 1.14 (0.47-2.72) | |
| AA | 6 (7.1) | 1.02 (0.34-3.05) | 1.00 (0.33-3.02) | | 1.03 (0.34-3.10) | |
| Rs6484320 |  |  |  | |  | |
| TT | 8 (6.9) | 1 | 1 | | 1 | |
| TA | 16 (6.8) | 0.99 (0.41-2.38) | 1.05 (0.43-2.55) | | 1.05 (0.43-2.55) | |
| AA | 8 (8.7) | 1.29 (0.46-3.57) | 1.21 (0.43-3.38) | | 1.24 (0.44-3.49) | |
|  |  | HSI 1-3 (n=559) | | | | |
| Rs6265 |  |  |  | |  | |
| AA | 13 (8.8) | 1 | 1 | | 1 | |
| GA | 26 (9.3) | 1.06 (0.53-2.12) | 1.06 (0.52-2.15) | | 1.05 (0.52-2.15) | |
| GG | 16 (12.1) | 1.42 (0.66-3.08) | 1.57 (0.72-3.46) | | 1.58 (0.72-3.47) | |
| Rs4923457 |  |  |  | |  | |
| TT | 10 (6.9) | 1 | 1 | | 1 | |
| AT | 27 (10.1) | 1.51 (0.71-3.21) | 1.46 (0.68-3.14) | | 1.46 (0.68-3.14) | |
| AA | 18 (12.2) | 1.86 (0.83-4.17) | 1.92 (0.85-4.36) | | 1.94 (0.86-4.42) | |
| Rs11030104 |  |  |  | |  | |
| GG | 12 (8.3) | 1 | 1 | | 1 | |
| AG | 26 (9.1) | 1.10 (0.54-2.24) | 1.02 (0.49-2.10) | | 1.00 (0.48-2.09) | |
| AA | 17 (13.3) | 1.69 (0.77-3.68) | 1.78 (0.81-3.93) | | 1.80 (0.81-3.97) | |
| Rs6484320 |  |  |  | |  | |
| TT | 13 (9.2) | 1 | 1 | | 1 | |
| TA | 27 (9.6) | 1.06 (0.53-2.11) | 1.02 (0.50-2.06) | | 1.01 (0.50-2.06) | |
| AA | 15 (11.0) | 1.23 (0.56-2.69) | 1.32 (0.59-2.91) | | 1.32 (0.60-2.92) | |
|  |  | HSI ≥ 4 (n=231) | | | | |
| Rs6265 |  |  | |  | |  |
| AA | 7 (10.0) | 1 | | 1 | | 1 |
| GA | 11 (10.7) | 1.08 (0.40-2.93) | | 1.11 (0.41-3.02) | | 1.10 (0.39-3.08) |
| GG | 16 (27.6) | 3.43^*^ (1.30-9.05) | | 3.47* (1.30-9.27) | | 3.54* (1.31-9.59) |
| Rs4923457 |  |  | |  | |  |
| TT | 6 (11.1) | 1 | | 1 | | 1 |
| AT | 12 (10.7) | 0.96 (0.34-2.71) | | 0.97 (0.34-2.76) | | 0.94 (0.33-2.73) |
| AA | 16 (24.6) | 2.61 (0.94-7.24) | | 2.77 (0.97-7.86) | | 2.76 (0.97-7.88) |
| Rs11030104 |  |  | |  | |  |
| GG | 6 (9.5) | 1 | | 1 | | 1 |
| AG | 15 (13.4) | 1.47 (0.54-4.00) | | 1.43 (0.52-3.91) | | 1.44 (0.51-4.06) |
| AA | 13 (23.2) | 2.87* (1.01-8.17) | | 2.85 (0.99-8.24) | | 2.92 (0.99-8.59) |
| Rs6484320 |  |  | |  | |  |
| TT | 7 (10.4) | 1 | | 1 | | 1 |
| TA | 13 (12.5) | 1.22 (0.46-3.25) | | 1.22 (0.46-3.25) | | 1.22 (0.45-3.34) |
| AA | 14 (23.3) | 2.61 (0.97-6.99) | | 2.66 (0.98-7.26) | | 2.70 (0.98-7.46) |
| P for trend of rs6265 GG |  | 0.001 | | <0.001 | | <0.001 |
| P for trend of rs4923457 AA |  | 0.004 | | 0.001 | | 0.001 |
| P for trend of rs11030104 AA |  | 0.01 | | 0.002 | | 0.01 |
| P for trend of rs6484320 AA |  | 0.02 | | 0.002 | | 0.003 |
| ‡General obesity was defined as a body mass index (BMI) ≥ 28 kg/m2 using the criteria for the Asian population.  Model A: Crude model Model B: Adjusted for age, sex Model C: Adjusted for age, sex, education, income level and physical activity  *P<0.05 | | | | | | |

**Table S8 Association of SNPs with general obesity in different Fagerstrom Test for Nicotine Dependence** (**FTND) score groups before cessation in former smokers**

|  | General obesity‡ n(%) | Model A | Model B | Model C |
| --- | --- | --- | --- | --- |
| FTND 0 (n=416) | | | | |
| Rs6265 |  |  |  |  |
| AA | 8 (7.0) | 1 | 1 | 1 |
| GA | 17 (7.8) | 1.12 (0.47-2.68) | 1.20 (0.50-2.89) | 1.21 (0.50-2.93) |
| GG | 6 (7.1) | 1.02 (0.34-3.06) | 0.98 (0.32-2.95) | 1.00 (0.33-3.04) |
| Rs4923457 |  |  |  |  |
| TT | 9 (7.9) | 1 | 1 | 1 |
| AT | 14 (6.7) | 0.84 (0.35-2.01) | 0.87 (0.36-2.10) | 0.86 (0.36-2.07) |
| AA | 8 (8.5) | 1.09 (0.40-2.93) | 1.09 (0.40-2.97) | 1.14 (0.42-3.11) |
| Rs11030104 |  |  |  |  |
| GG | 8 (7.3) | 1 | 1 | 1 |
| AG | 17 (7.6) | 1.04 (0.44-2.50) | 1.11 (0.46-2.68) | 1.05 (0.35-3.17) |
| AA | 6 (7.4) | 1.02 (0.34-3.06) | 1.01 (0.33-3.05) | 0.96 (0.90-1.02) |
| Rs6484320 |  |  |  |  |
| TT | 8 (7.1) | 1 | 1 | 1 |
| TA | 15 (6.9) | 0.97 (0.40-2.35) | 1.02 (0.42-2.51) | 1.04 (0.42-2.55) |
| AA | 8 (9.2) | 1.32 (0.47-3.66) | 1.25 (0.45-3.50) | 1.29 (0.46-3.63) |
| FTND 1-3 (n=489) | | | | |
| Rs6265 |  |  |  |  |
| AA | 12 (9.3) | 1 | 1 | 1 |
| GA | 20 (8.1) | 0.86 (0.41-1.82) | 0.78 (0.37-1.68) | 0.78 (0.36-1.68) |
| GG | 14 (12.4) | 1.38 (0.61-3.12) | 1.44 (0.63-3.27) | 1.45 (0.63-3.30) |
| Rs4923457 |  |  |  |  |
| TT | 10 (8.1) | 1 | 1 | 1 |
| AT | 20 (8.5) | 1.05 (0.47-2.31) | 0.98 (0.44-2.17) | 0.97 (0.44-2.18) |
| AA | 16 (12.3) | 1.59 (0.69-3.64) | 1.60 (0.69-3.69) | 1.65 (0.71-3.82) |
| Rs11030104 |  |  |  |  |
| GG | 11 (8.6) | 1 | 1 | 1 |
| AG | 21 (8.3) | 0.96 (0.45-2.06) | 0.85 (0.39-1.85) | 0.85 (0.39-1.85) |
| AA | 14 (13.1) | 1.60 (0.69-3.69) | 1.62 (0.70-3.76) | 1.66 (0.72-3.87) |
| Rs6484320 |  |  |  |  |
| TT | 12 (9.8) | 1 | 1 | 1 |
| TA | 21 (8.4) | 0.85 (0.40-1.79) | 0.77 (0.36-1.65) | 0.78 (0.36-1.65) |
| AA | 13 (11.2) | 1.17 (0.51-2.68) | 1.18 (0.51-2.72) | 1.19 (0.52-2.74) |
| FTND ≥ 4 (n=328) | | | | |
| Rs6265 |  |  |  |  |
| AA | 8 (8.7) | 1 | 1 | 1 |
| GA | 18 (11.6) | 1.38 (0.58-3.31) | 1.61 (0.65-3.98) | 1.70 (0.67-4.32) |
| GG | 18 (22.2) | 3.00* (1.23-7.34) | 3.47* (1.37-8.80) | 3.64* (1.40-9.45) |
| Rs4923457 |  |  |  |  |
| TT | 6 (7.8) | 1 | 1 | 1 |
| AT | 20 (12.3) | 1.67 (0.64-4.33) | 1.80 (0.68-4.78) | 1.84 (0.68-5.00) |
| AA | 18 (20.2) | 3.00* (1.13-8.00) | 3.49* (1.27-9.60) | 3.57* (1.28-9.95) |
| Rs11030104 |  |  |  |  |
| GG | 7 (8.3) | 1 | 1 | 1 |
| AG | 21 (12.9) | 1.63 (0.66-4.00) | 1.67 (0.67-4.18) | 1.76 (0.68-4.58) |
| AA | 16 (19.8) | 2.71* (1.05-6.98) | 2.96* (1.12-7.84) | 3.11* (1.14-8.49) |
| Rs6484320 |  |  |  |  |
| TT | 8 (8.9) | 1 | 1 | 1 |
| TA | 20 (13.1) | 1.54 (0.65-3.66) | 1.74 (0.71-4.24) | 1.82 (0.72-4.58) |
| AA | 16 (18.8) | 2.38 (0.96-5.89) | 2.80 (1.09-7.20) | 2.92* (1.11-7.69) |
| P for trend of rs6265 GG |  | 0.01 | 0.002 | 0.003 |
| P for trend of rs4923457 AA |  | 0.02 | 0.01 | 0.01 |
| P for trend of rs11030104 AA |  | 0.02 | 0.02 | 0.01 |
| P for trend of rs6484320 AA |  | 0.06 | 0.02 | 0.02 |
| ‡General obesity was defined as a body mass index (BMI) ≥ 28 kg/m2 using the criteria for the Asian population.  Model A: Crude model Model B: Adjusted for age, sex Model C: Adjusted for age, sex, education, income level and physical activity *P<0.05 | | | | |

**Table S9 Association of SNPs with central obesity in different cigarettes per day (CPD) groups before cessation in former smokers**

|  | Central obesity‡ n(%) | Model A | Model B | Model C |
| --- | --- | --- | --- | --- |
| CPD 1-9 (n=420) | | | | |
| Rs6265 |  |  |  |  |
| AA | 40 (39.2) | 1 | 1 | 1 |
| GA | 94 (40.3) | 1.05 (0.65-1.69) | 1.20 (0.71-2.02) | 1.21 (0.72-2.04) |
| GG | 37 (43.5) | 1.20 (0.67-2.14) | 1.36 (0.72-2.58) | 1.36 (0.72-2.56) |
| Rs4923457 |  |  |  |  |
| TT | 41 (41.0) | 1 | 1 | 1 |
| AT | 87 (39.9) | 0.96 (0.59-1.55) | 1.07 (0.63-1.81) | 1.08 (0.64-1.84) |
| AA | 43 (42.2) | 1.05 (0.60-1.84) | 1.24 (0.67-2.28) | 1.24 (0.67-2.28) |
| Rs11030104 |  |  |  |  |
| GG | 41 (41.0) | 1 | 1 | 1 |
| AG | 93 (39.2) | 0.93 (0.58-1.50) | 1.02 (0.60-1.71) | 1.02 (0.61-1.72) |
| AA | 37 (44.6) | 1.16 (0.64-2.09) | 1.33 (0.70-2.52) | 1.33 (0.70-2.51) |
| Rs6484320 |  |  |  |  |
| TT | 41 (41.4) | 1 | 1 | 1 |
| TA | 92 (40.2) | 0.95 (0.59-1.53) | 1.07 (0.63-1.80) | 1.07 (0.63-1.81) |
| AA | 38 (41.3) | 1.00 (0.56-1.77) | 1.07 (0.57-2.01) | 1.07 (0.57-2.00) |
| CPD 10-19 (n=314) | | | | |
| Rs6265 |  |  |  |  |
| AA | 28 (28.9) | 1 | 1 | 1 |
| GA | 52 (33.8) | 1.25 (0.72-2.18) | 1.27 (0.72-2.25) | 1.30 (0.73-2.31) |
| GG | 33 (52.4) | 2.71* (1.40-5.25) | 2.88* (1.45-5.71) | 2.97* (1.49-5.91) |
| Rs4923457 |  |  |  |  |
| TT | 29 (31.5) | 1 | 1 | 1 |
| AT | 51 (33.3) | 1.09 (0.62-1.89) | 1.10 (0.62-1.96) | 1.12 (0.63-2.00) |
| AA | 33 (47.8) | 1.99* (1.04-3.80) | 2.17* (1.11-4.23) | 2.20* (1.12-4.31) |
| Rs11030104 |  |  |  |  |
| GG | 27 (29.3) | 1 | 1 | 1 |
| AG | 55 (33.7) | 1.23 (0.71-2.13) | 1.12 (0.63-1.99) | 1.15 (0.65-2.05) |
| AA | 31 (52.5) | 2.67* (1.35-5.26) | 2.68* (1.33-5.39) | 2.79* (1.38-5.65) |
| Rs6484320 |  |  |  |  |
| TT | 28 (29.2) | 1 | 1 | 1 |
| TA | 53 (34.2) | 1.26 (0.73-2.19) | 1.29 (0.73-2.27) | 1.30 (0.73-2.31) |
| AA | 32 (50.8) | 2.51* (1.29-4.86) | 2.52* (1.27-4.98) | 2.58* (1.30-5.13) |
| CPD ≥ 20 (n=499) | | | | |
| Rs6265 |  |  |  |  |
| AA | 43 (31.6) | 1 | 1 | 1 |
| GA | 101 (43.3) | 1.65* (1.06-2.58) | 1.72* (1.08-2.74) | 1.77* (1.11-2.83) |
| GG | 64 (49.2) | 2.10* (1.27-3.45) | 2.22* (1.32-3.73) | 2.27* (1.35-3.81) |
| Rs4923457 |  |  |  |  |
| TT | 38 (31.1) | 1 | 1 | 1 |
| AT | 106 (45.1) | 1.82* (1.15-2.88) | 1.74* (1.08-2.80) | 1.78* (1.10-2.87) |
| AA | 64 (45.1) | 1.81* (1.09-3.01) | 1.82* (1.08-3.07) | 1.86* (1.10-3.14) |
| Rs11030104 |  |  |  |  |
| GG | 41 (31.5) | 1 | 1 | 1 |
| AG | 105 (50.5) | 1.66* (1.06-2.61) | 1.62* (1.02-2.58) | 1.68* (1.05-2.69) |
| AA | 62 (48.8) | 2.07* (1.25-3.44) | 2.13* (1.26-3.59) | 2.19* (1.29-3.70) |
| Rs6484320 |  |  |  |  |
| TT | 41 (31.5) | 1 | 1 | 1 |
| TA | 105 (44.5) | 1.74* (1.11-2.73) | 1.70* (1.07-2.71) | 1.76* (1.10-2.82) |
| AA | 62 (46.6) | 1.90* (1.15-3.13) | 1.96* (1.17-3.29) | 1.98* (1.18-3.34) |
| P for trend of rs6265 GG |  | 0.46 | 0.06 | 0.06 |
| P for trend of rs4923457 AA |  | 0.69 | 0.09 | 0.09 |
| P for trend of rs11030104 AA |  | 0.60 | 0.10 | 0.11 |
| P for trend of rs6484320 AA |  | 0.48 | 0.05 | 0.05 |
| ‡Central obesity was based on the criteria for the Asian population (waist circumference [WC] ≥ 90 cm in men and ≥ 80 cm in women)  Model A: Crude model Model B: Adjusted for age, sex Model C: Adjusted for age, sex, education, income level and physical activity  *P<0.05 | | | | |

**Table S10 Association of SNPs with central obesity in different Heavy Smoking Index (HSI) score groups before cessation in former smokers**

|  | Central obesity‡ n(%) | Model A | Model B | | Model C | | |
| --- | --- | --- | --- | --- | --- | --- | --- |
| HIS=0 (n=443) | | | | | | | |
| Rs6265 |  |  | |  | | |  |
| AA | 44 (37.3) | 1 | | 1 | | | 1 |
| GA | 87 (36.7) | 0.98 (0.62-1.54) | | 1.09 (0.67-1.78) | | | 1.09 (0.66-1.78) |
| GG | 41 (46.6) | 1.47 (0.84-2.57) | | 1.54 (0.84-2.81) | | | 1.52 (0.83-2.79) |
| Rs4923457 |  |  | |  | | |  |
| TT | 44 (37.9) | 1 | | 1 | | | 1 |
| AT | 84 (37.0) | 0.96 (0.61-1.53) | | 1.04 (0.64-1.71) | | | 1.05 (0.64-1.72) |
| AA | 44 (44.0) | 1.29 (0.75-2.22) | | 1.46 (0.81-2.63) | | | 1.46 (0.81-2.62) |
| Rs11030104 |  |  | |  | | |  |
| GG | 44 (38.3) | 1 | | 1 | | | 1 |
| AG | 88 (36.2) | 0.92 (0.58-1.45) | | 1.00 (0.61-1.63) | | | 1.00 (0.61-1.63) |
| AA | 40 (47.1) | 1.43 (0.81-2.53) | | 1.55 (0.84-2.85) | | | 1.53 (0.83-2.83) |
| Rs6484320 |  |  | |  | | |  |
| TT | 44 (37.9) | 1 | | 1 | | | 1 |
| TA | 86 (36.6) | 0.94 (0.60-1.50) | | 1.06 (0.65-1.73) | | | 1.06 (0.64-1.73) |
| AA | 42 (45.7) | 1.38 (0.79-2.40) | | 1.34 (0.74-2.43) | | | 1.33 (0.73-2.41) |
| HSI 1-3 (n=559) | | | | | | | |
| Rs6265 |  |  | |  | | |  |
| AA | 42 (28.6) | 1 | | 1 | | | 1 |
| GA | 121 (43.2) | 1.90* (1.24-2.92) | | 1.91* (1.22-2.99) | | | 1.95* (1.24-3.06) |
| GG | 60 (45.5) | 2.08* (1.27-3.42) | | 2.36* (1.41-3.95) | | | 2.44* (1.45-4.10) |
| Rs4923457 |  |  | |  | | |  |
| TT | 45 (31.3) | 1 | | 1 | | | 1 |
| AT | 115 (43.1) | 1.66* (1.09-2.55) | | 1.61* (1.04-2.53) | | | 1.66* (1.06-2.59) |
| AA | 63 (42.6) | 1.63* (1.01-2.64) | | 1.69* (1.03-2.78) | | | 1.78* (1.08-2.93) |
| Rs11030104 |  |  | |  | | |  |
| GG | 42 (29.2) | 1 | | 1 | | | 1 |
| AG | 121 (42.2) | 1.77* (1.15-2.72) | | 1.64* (1.05-2.56) | | | 1.69* (1.08-2.65) |
| AA | 60 (46.9) | 2.14* (1.30-3.53) | | 2.28* (1.36-3.82) | | | 2.40* (1.43-4.04) |
| Rs6484320 |  |  | |  | | |  |
| TT | 43 (30.3) | 1 | | 1 | | | 1 |
| TA | 122 (43.4) | 1.77* (1.15-2.71) | | 1.72* (1.10-2.68) | | | 1.77* (1.13-2.78) |
| AA | 58 (42.6) | 1.71* (1.05-2.81) | | 1.85* (1.11-3.08) | | | 1.92* (1.15-3.21) |
| HSI ≥ 4 (n=231) | | | | | | | |
| Rs6265 |  |  | |  | |  | |
| AA | 25 (35.7) | 1 | | 1 | | 1 | |
| GA | 39 (37.9) | 1.10 (0.58-2.06) | | 1.15 (0.60-2.22) | | 1.18 (0.60-2.31) | |
| GG | 33 (56.9) | 2.38* (1.16-4.85) | | 2.39* (1.13-5.04) | | 2.40* (1.13-5.12) | |
| Rs4923457 |  |  | |  | |  | |
| TT | 19 (35.2) | 1 | | 1 | | 1 | |
| AT | 45 (40.2) | 1.24 (0.63-2.43) | | 1.23 (0.61-2.48) | | 1.26 (0.61-2.57) | |
| AA | 33 (50.8) | 1.90 (0.91-3.98) | | 1.94 (0.90-4.22) | | 1.95 (0.89-4.26) | |
| Rs11030104 |  |  | |  | |  | |
| GG | 23 (36.5) | 1 | | 1 | | 1 | |
| AG | 44 (45.4) | 1.13 (0.60-2.13) | | 1.02 (0.53-1.96) | | 1.02 (0.52-2.00) | |
| AA | 30 (53.6) | 2.01 (0.96-4.18) | | 1.89 (0.89-4.02) | | 1.89 (0.88-4.07) | |
| Rs6484320 |  |  | |  | |  | |
| TT | 23 (34.3) | 1 | | 1 | | 1 | |
| TA | 42 (40.4) | 1.30 (0.68-2.45) | | 1.25 (0.65-2.43) | | 1.28 (0.65-2.53) | |
| AA | 32 (53.3) | 2.19* (1.07-4.47) | | 2.18* (1.04-4.59) | | 2.20* (1.03-4.66) | |
| P for trend of rs6265 GG |  | 0.28 | | 0.04 | | 0.03 | |
| P for trend of rs4923457 AA |  | 0.46 | | 0.07 | | 0.08 | |
| P for trend of rs11030104 AA |  | 0.49 | | 0.09 | | 0.10 | |
| P for trend of rs6484320 AA |  | 0.44 | | 0.05 | | 0.05 | |
| ‡Central obesity was based on the criteria for the Asian population (waist circumference [WC] ≥ 90 cm in men and ≥ 80 cm in women)  Model A: Crude model Model B: Adjusted for age, sex Model C: Adjusted for age, sex, education, income level and physical activity *P<0.05 | | | | | | | |

**Table S11 Association of SNPs with central obesity in different Fagerstrom Test for Nicotine Dependence (FTND) score groups before cessation in former smokers**

|  | Central obesity‡ n(%) | Model A | Model B | | | Model C | | | |
| --- | --- | --- | --- | --- | --- | --- | --- | --- | --- |
| FTND 0 (n=416) | | | | | | | | | |
| Rs6265 |  |  | | |  | | | |  |
| AA | 42 (32.8) | 1 | | | 1 | | | | 1 |
| GA | 79 (36.2) | 0.97 (0.61-1.56) | | | 1.11 (0.67-1.84) | | | | 1.10 (0.67-1.83) |
| GG | 40 (47.6) | 1.56 (0.88-2.76) | | | 1.64 (0.89-3.04) | | | | 1.62 (0.88-3.01) |
| Rs4923457 |  |  | | |  | | | |  |
| TT | 42 (36.8) | 1 | | | 1 | | | | 1 |
| AT | 76 (36.5) | 0.99 (0.62-1.59) | | | 1.09 (0.65-1.81) | | | | 1.09 (0.65-1.82) |
| AA | 43 (45.7) | 1.45 (0.83-2.52) | | | 1.65 (0.91-3.00) | | | | 1.65 (0.90-3.00) |
| Rs11030104 |  |  | | |  | | | |  |
| GG | 42 (38.2) | 1 | | | 1 | | | | 1 |
| AG | 80 (35.6) | 0.89 (0.56-1.43) | | | 1.00 (0.60-1.65) | | | | 0.99 (0.60-1.65) |
| AA | 39 (48.1) | 1.50 (0.84-2.69) | | | 1.64 (0.88-3.06) | | | | 1.62 (0.87-3.03) |
| Rs6484320 |  |  | | |  | | | |  |
| TT | 42 (37.5) | 1 | | | 1 | | | | 1 |
| TA | 78 (35.9) | 0.94 (0.58-1.50) | | | 1.06 (0.64-1.76) | | | | 1.05 (0.63-1.75) |
| AA | 41 (47.1) | 1.49 (0.84-2.62) | | | 1.48 (0.80-2.73) | | | | 1.46 (0.79-2.69) |
| FTND 1-3 (n=489) | | | | | | | | | |
| Rs6265 |  |  | | |  | | |  | |
| AA | 37 (28.7) | 1 | | | 1 | | | 1 | |
| GA | 107 (43.3) | 1.90* (1.20-3.00) | | | 1.78* (1.10-2.86) | | | 1.77* (1.09-2.87) | |
| GG | 50 (44.2) | 1.97* (1.16-3.36) | | | 2.20* (1.27-3.81) | | | 2.22* (1.28-3.87) | |
| Rs4923457 |  |  | | |  | | |  | |
| TT | 41 (33.3) | 1 | | | 1 | | | 1 | |
| AT | 99 (41.9) | 1.45 (0.92-2.28) | | | 1.33 (0.83-2.14) | | | 1.32 (0.82-2.13) | |
| AA | 54 (41.5) | 1.42 (0.85-2.37) | | | 1.47 (0.87-2.49) | | | 1.53 (0.90-2.62) | |
| Rs11030104 |  |  | | |  | | |  | |
| GG | 36 (28.1) | 1 | | | 1 | | | 1 | |
| AG | 109 (42.9) | 1.92* (1.22-3.04) | | | 1.69* (1.05-2.73) | | | 1.74* (1.08-2.82) | |
| AA | 49 (45.8) | 2.16* (1.26-3.71) | | | 2.29* (1.31-4.00) | | | 2.40* (1.37-4.21) | |
| Rs6484320 |  |  | | |  | | |  | |
| TT | 37 (30.1) | 1 | | | 1 | | | 1 | |
| TA | 109 (43.6) | 1.80* (1.14-2.84) | | | 1.64* (1.02-2.64) | | | 1.68* (1.04-2.71) | |
| AA | 48 (41.4) | 1.64 (0.96-2.80) | | | 1.72 (0.99-2.98) | | | 1.75* (1.01-3.05) | |
| FTND ≥ 4 (n=328) | | | | | | | | | |
| Rs6265 |  |  | |  | | |  | | |
| AA | 32 (34.8) | 1 | | 1 | | | 1 | | |
| GA | 61 (39.4) | 1.22 (0.71-2.08) | | 1.36 (0.77-2.37) | | | 1.37 (0.77-2.44) | | |
| GG | 44 (54.3) | 2.23* (1.21-4.11) | | 2.32* (1.22-4.41) | | | 2.32* (1.21-4.45) | | |
| Rs4923457 |  |  | |  | | |  | | |
| TT | 25 (32.5) | 1 | | 1 | | | 1 | | |
| AT | 69 (42.6) | 1.54 (0.87-2.73) | | 1.63 (0.90-2.96) | | | 1.63 (0.89-2.98) | | |
| AA | 43 (48.3) | 1.94* (1.03-3.66) | | 2.02* (1.05-3.92) | | | 2.01* (1.03-3.91) | | |
| Rs11030104 |  |  | |  | | |  | | |
| GG | 31 (36.9) | 1 | | 1 | | | 1 | | |
| AG | 64 (39.3) | 1.11 (0.64-1.90) | | 1.08 (0.61-1.89) | | | 1.07 (0.60-1.90) | | |
| AA | 42 (51.9) | 1.84 (0.99-3.43) | | 1.80 (0.95-3.41) | | | 1.77 (0.93-3.39) | | |
| Rs6484320 |  |  | |  | | |  | | |
| TT | 31 (34.4) | 1 | | 1 | | | 1 | | |
| TA | 63 (41.2) | 1.33 (0.78-2.29) | | 1.41 (0.80-2.48) | | | 1.42 (0.80-2.53) | | |
| AA | 43 (50.6) | 1.95* (1.06-3.58) | | 2.02* (1.07-3.81) | | | 2.02* (1.06-3.83) | | |
| P for trend of rs6265 GG |  | 0.40 | | 0.060 | | | 0.04 | | |
| P for trend of rs4923457 AA |  | 0.74 | | 0.18 | | | 0.21 | | |
| P for trend of rs11030104 AA |  | 0.64 | | 0.15 | | | 0.19 | | |
| P for trend of rs6484320 AA |  | 0.66 | | 0.12 | | | 0.15 | | |
| ‡Central obesity was based on the criteria for the Asian population (waist circumference [WC] ≥ 90 cm in men and ≥ 80 cm in women)  Model A: Crude model Model B: Adjusted for age, sex Model C: Adjusted for age, sex, education, income level and physical activity *P<0.05 | | | | | | | | | |

**Table S6-1 Association of SNPs with general obesity in different cigarettes per day (CPD) groups before cessation in former smokers (additive model)**

|  | CPD 1-9 (n=420) | | | CPD 10-19 (n=314) | | | CPD ≥20 (n=499) | | |
| --- | --- | --- | --- | --- | --- | --- | --- | --- | --- |
|  | Model A | Model B | Model C | Model A | Model B | Model C | Model A | Model B | Model C |
| Rs6265 |  |  |  |  |  |  |  |  |  |
| A | 1 | 1 | 1 | 1 | 1 | 1 | 1 | 1 | 1 |
| G | 1.04 (0.63-1.71) | 1.05 (0.64-1.72) | 1.04 (0.63-1.71) | 1.26 (0.65-2.42) | 1.27 (0.66-2.45) | 1.3 (0.67-2.50) | 1.49 (1.03-2.15) | 1.52 (1.05-2.19) | 1.53 (1.05-2.21) |
| P | 0.891 | 0.844 | 0.888 | 0.491 | 0.481 | 0.438 | 0.032 | 0.028 | 0.026 |
| Rs4923457 |  |  |  |  |  |  |  |  |  |
| T | 1 | 1 | 1 | 1 | 1 | 1 | 1 | 1 | 1 |
| A | 0.93 (0.58-1.51) | 0.96 (0.59-1.55) | 0.93 (0.57-1.52) | 1.45 (0.76-2.80) | 1.49 (0.77-2.88) | 1.52 (0.78-2.94) | 1.60 (1.1-2.33) | 1.63 (1.11-2.39) | 1.66 (1.13-2.44) |
| P | 0.772 | 0.862 | 0.780 | 0.263 | 0.239 | 0.216 | 0.014 | 0.012 | 0.010 |
| Rs11030104 |  |  |  |  |  |  |  |  |  |
| G | 1 | 1 | 1 | 1 | 1 | 1 | 1 | 1 | 1 |
| A | 1.04 (0.62-1.72) | 1.06 (0.64-1.75) | 1.05 (0.63-1.75) | 1.27 (0.65-2.49) | 1.25 (0.63-2.49) | 1.26 (0.63-2.50) | 1.49 (1.03-2.17) | 1.51 (1.04-2.21) | 1.53 (1.04-2.23) |
| P | 0.890 | 0.819 | 0.836 | 0.491 | 0.530 | 0.509 | 0.035 | 0.031 | 0.029 |
| Rs6484320 |  |  |  |  |  |  |  |  |  |
| T | 1 | 1 | 1 | 1 | 1 | 1 | 1 | 1 | 1 |
| A | 1.11 (0.68-1.82) | 1.12 (0.68-1.82) | 1.10 (0.67-1.8) | 1.25 (0.65-2.41) | 1.22 (0.63-2.36) | 1.24 (0.64-2.41) | 1.3 (0.90-1.87) | 1.32 (0.91-1.91) | 1.32 (0.91-1.91) |
| P | 0.680 | 0.658 | 0.705 | 0.503 | 0.552 | 0.515 | 0.161 | 0.144 | 0.145 |
| Model A: Crude model Model B: Adjusted for age, sex Model C: Adjusted for age, sex, education, income level and physical activity | | | | | | | | | |

**Table S7-1 Association of SNPs with general obesity in different Heavy Smoking Index (HSI) score groups before cessation in former smokers (additive model)**

|  | HSI=0 (n=443) | | | HSI 1-3 (n=559) | | | HSI ≥ 4 (n=231) | | |
| --- | --- | --- | --- | --- | --- | --- | --- | --- | --- |
|  | Model A | Model B | Model C | Model A | Model B | Model C | Model A | Model B | Model C |
| Rs6265 |  |  |  |  |  |  |  |  |  |
| A | 1 | 1 | 1 | 1 | 1 | 1 | 1 | 1 | 1 |
| G | 1.01 (0.6-1.72) | 1.00 (0.59-1.68) | 1.00 (0.59-1.7) | 1.20 (0.81-1.78) | 1.26 (0.84-1.90) | 1.27 (0.84-1.90) | 2.00 (1.19-3.35) | 2.00 (1.19-3.37) | 2.04 (1.2-3.45) |
| P | 0.964 | 0.991 | 0.985 | 0.369 | 0.258 | 0.256 | 0.008 | 0.009 | 0.008 |
| Rs4923457 |  |  |  |  |  |  |  |  |  |
| T | 1 | 1 | 1 | 1 | 1 | 1 | 1 | 1 | 1 |
| A | 1.01 (0.6-1.69) | 1.02 (0.61-1.70) | 1.03 (0.61-1.73) | 1.34 (0.91-1.99) | 1.38 (0.92-2.05) | 1.39 (0.93-2.07) | 1.80 (1.05-3.07) | 1.85 (1.07-3.21) | 1.86 (1.07-3.23) |
| P | 0.967 | 0.950 | 0.921 | 0.137 | 0.115 | 0.110 | 0.032 | 0.029 | 0.027 |
| Rs11030104 |  |  |  |  |  |  |  |  |  |
| G | 1 | 1 | 1 | 1 | 1 | 1 | 1 | 1 | 1 |
| A | 1.01 (0.59-1.73) | 1.01 (0.59-1.72) | 1.02 (0.60-1.75) | 1.31 (0.88-1.96) | 1.36 (0.90-2.06) | 1.37 (0.90-2.08) | 1.73 (1.03-2.93) | 1.73 (1.02-2.96) | 1.76 (1.02-3.02) |
| P | 0.963 | 0.969 | 0.938 | 0.182 | 0.144 | 0.139 | 0.040 | 0.044 | 0.041 |
| Rs6484320 |  |  |  |  |  |  |  |  |  |
| T | 1 | 1 | 1 | 1 | 1 | 1 | 1 | 1 | 1 |
| A | 1.13 (0.67-1.92) | 1.10 (0.65-1.85) | 1.11 (0.66-1.88) | 1.11 (0.75-1.65) | 1.15 (0.77-1.73) | 1.15 (0.77-1.73) | 1.67 (1.01-2.77) | 1.69 (1.01-2.83) | 1.71 (1.01-2.88) |
| P | 0.642 | 0.723 | 0.687 | 0.602 | 0.497 | 0.493 | 0.047 | 0.046 | 0.044 |
| Model A: Crude model Model B: Adjusted for age, sex Model C: Adjusted for age, sex, education, income level and physical activity | | | | | | | | | |

**Table S8-1 Association of SNPs with general obesity in different Fagerstrom Test for Nicotine Dependence (FTND) score groups before cessation in former smokers (additive model)**

|  | FTND 0 (n=416) | | | FTND 1-3 (n=489) | | | FTND ≥ 4 (n=328) | | |
| --- | --- | --- | --- | --- | --- | --- | --- | --- | --- |
|  | Model A | Model B | Model C | Model A | Model B | Model C | Model A | Model B | Model C |
| Rs6265 |  |  |  |  |  |  |  |  |  |
| A | 1 | 1 | 1 | 1 | 1 | 1 | 1 | 1 | 1 |
| G | 1.02 (0.6-1.73) | 1.00 (0.59-1.70) | 1.02 (0.6-1.72) | 1.19 (0.77-1.83) | 1.21 (0.78-1.89) | 1.22 (0.78-1.90) | 1.80(1.14-2.83) | 1.90 (1.20-3.03) | 1.95 (1.21-3.12) |
| P | 0.949 | 0.991 | 0.955 | 0.440 | 0.394 | 0.385 | 0.012 | 0.007 | 0.006 |
| Rs4923457 |  |  |  |  |  |  |  |  |  |
| T | 1 | 1 | 1 | 1 | 1 | 1 | 1 | 1 | 1 |
| A | 1.03 (0.62-1.74) | 1.04 (0.62-1.74) | 1.06 (0.63-1.78) | 1.28 (0.84-1.97) | 1.30 (0.84-2.00) | 1.32 (0.85-2.04) | 1.75 (1.09-2.79) | 1.88 (1.16-3.06) | 1.90 (1.17-3.09) |
| P | 0.897 | 0.886 | 0.837 | 0.251 | 0.240 | 0.216 | 0.019 | 0.010 | 0.010 |
| Rs11030104 |  |  |  |  |  |  |  |  |  |
| G | 1 | 1 | 1 | 1 | 1 | 1 | 1 | 1 | 1 |
| A | 1.01 (0.59-1.74) | 1.01 (0.59-1.73) | 1.03 (0.60-1.77) | 1.28 (0.83-1.99) | 1.30 (0.83-2.04) | 1.32 (0.84-2.08) | 1.65 (1.04-2.61) | 1.73 (1.07-2.78) | 1.76 (1.09-2.86) |
| P | 0.964 | 0.964 | 0.915 | 0.266 | 0.257 | 0.237 | 0.033 | 0.024 | 0.021 |
| Rs6484320 |  |  |  |  |  |  |  |  |  |
| T | 1 | 1 | 1 | 1 | 1 | 1 | 1 | 1 | 1 |
| A | 1.15 (0.67-1.95) | 1.12 (0.66-1.89) | 1.14 (0.67-1.92) | 1.08 (0.70-1.68) | 1.09 (0.70-1.70) | 1.10 (0.70-1.71) | 1.54 (0.99-2.41) | 1.66 (1.05-2.64) | 1.69 (1.06-2.70) |
| P | 0.614 | 0.682 | 0.637 | 0.713 | 0.699 | 0.688 | 0.056 | 0.030 | 0.027 |
| Model A: Crude model Model B: Adjusted for age, sex Model C: Adjusted for age, sex, education, income level and physical activity | | | | | | | | | |

**Table S9-1 Association of SNPs with central obesity in different cigarettes per day (CPD) groups before cessation in former smokers (additive model)**

|  | CPD 1-9 (n=420) | | | CPD 10-19 (n=314) | | | CPD ≥20 (n=499) | | |
| --- | --- | --- | --- | --- | --- | --- | --- | --- | --- |
|  | Model A | Model B | Model C | Model A | Model B | Model C | Model A | Model B | Model C |
| Rs6265 |  |  |  |  |  |  |  |  |  |
| A | 1 | 1 | 1 | 1 | 1 | 1 | 1 | 1 | 1 |
| G | 1.09 (0.81-1.46) | 1.17 (0.85-1.60) | 1.17 (0.85-1.60) | 1.62 (1.16-2.27) | 1.67 (1.18-2.36) | 1.70 (1.20-2.41) | 1.44 (1.13-1.85) | 1.48 (1.15-1.92) | 1.50 (1.16-1.94) |
| P | 0.559 | 0.336 | 0.344 | 0.004 | 0.004 | 0.003 | 0.004 | 0.003 | 0.002 |
| Rs4923457 |  |  |  |  |  |  |  |  |  |
| T | 1 | 1 | 1 | 1 | 1 | 1 | 1 | 1 | 1 |
| A | 1.02 (0.77-1.36) | 1.11 (0.82-1.51) | 1.11 (0.82-1.51) | 1.40 (1.01-1.94) | 1.46 (1.04-2.05) | 1.47 (1.05-2.07) | 1.32 (1.03-1.69) | 1.33 (1.03-1.72) | 1.34 (1.04-1.73) |
| P | 0.865 | 0.490 | 0.491 | 0.044 | 0.029 | 0.026 | 0.028 | 0.029 | 0.025 |
| Rs11030104 |  |  |  |  |  |  |  |  |  |
| G | 1 | 1 | 1 | 1 | 1 | 1 | 1 | 1 | 1 |
| A | 1.07 (0.80-1.44) | 1.15 (0.83-1.58) | 1.15 (0.83-1.58) | 1.60 (1.14-2.26) | 1.60 (1.12-2.28) | 1.63 (1.14-2.33) | 1.43 (1.11-1.84) | 1.45 (1.12-1.89) | 1.47 (1.13-1.91) |
| P | 0.660 | 0.402 | 0.405 | 0.007 | 0.009 | 0.007 | 0.005 | 0.005 | 0.004 |
| Rs6484320 |  |  |  |  |  |  |  |  |  |
| T | 1 | 1 | 1 | 1 | 1 | 1 | 1 | 1 | 1 |
| A | 1.00 (0.75-1.33) | 1.04 (0.76-1.42) | 1.03 (0.76-1.41) | 1.56 (1.12-2.18) | 1.57 (1.11-2.21) | 1.59 (1.12-2.25) | 1.37 (1.07-1.75) | 1.39 (1.07-1.79) | 1.39 (1.08-1.80) |
| P | 0.982 | 0.822 | 0.833 | 0.009 | 0.010 | 0.009 | 0.014 | 0.012 | 0.011 |
| Model A: Crude model Model B: Adjusted for age, sex Model C: Adjusted for age, sex, education, income level and physical activity | | | | | | | | | |

**Table S10-1 Association of SNPs with central obesity in different Heavy Smoking Index (HSI) score groups before cessation in former smokers (additive model)**

|  | HSI=0 (n=443) | | | HSI 1-3 (n=559) | | | HSI ≥ 4 (n=231) | | |
| --- | --- | --- | --- | --- | --- | --- | --- | --- | --- |
|  | Model A | Model B | Model C | Model A | Model B | Model C | Model A | Model B | Model C |
| Rs6265 |  |  |  |  |  |  |  |  |  |
| A | 1 | 1 | 1 | 1 | 1 | 1 | 1 | 1 | 1 |
| G | 1.20 (0.90-1.59) | 1.23 (0.91-1.66) | 1.22 (0.90-1.66) | 1.44 (1.13-1.83) | 1.53 (1.18-1.97) | 1.55 (1.20-2.00) | 1.53 (1.07-2.19) | 1.54 (1.05-2.24) | 1.55 (1.06-2.27) |
| P | 0.215 | 0.181 | 0.191 | 0.004 | 0.001 | 0.001 | 0.020 | 0.025 | 0.025 |
| Rs4923457 |  |  |  |  |  |  |  |  |  |
| T | 1 | 1 | 1 | 1 | 1 | 1 | 1 | 1 | 1 |
| A | 1.13 (0.86-1.49) | 1.21 (0.90-1.62) | 1.20 (0.90-1.62) | 1.27 (1.00-1.60) | 1.29 (1.01-1.65) | 1.32 (1.03-1.69) | 1.39 (0.96-2.01) | 1.41 (0.95-2.07) | 1.41 (0.95-2.08) |
| P | 0.385 | 0.214 | 0.220 | 0.050 | 0.041 | 0.027 | 0.082 | 0.086 | 0.086 |
| Rs11030104 |  |  |  |  |  |  |  |  |  |
| G | 1 | 1 | 1 | 1 | 1 | 1 | 1 | 1 | 1 |
| A | 1.18 (0.88-1.57) | 1.23 (0.90-1.67) | 1.22 (0.90-1.66) | 1.46 (1.14-1.87) | 1.51 (1.17-1.95) | 1.55 (1.19-2.00) | 1.41 (0.98-2.05) | 1.37 (0.93-2.00) | 1.38 (0.93-2.03) |
| P | 0.265 | 0.194 | 0.203 | 0.003 | 0.002 | 0.001 | 0.066 | 0.107 | 0.105 |
| Rs6484320 |  |  |  |  |  |  |  |  |  |
| T | 1 | 1 | 1 | 1 | 1 | 1 | 1 | 1 | 1 |
| A | 1.16 (0.88-1.54) | 1.15 (0.85-1.55) | 1.15 (0.85-1.55) | 1.30 (1.02-1.66) | 1.35 (1.05-1.74) | 1.37 (1.07-1.77) | 1.48 (1.03-2.12) | 1.48 (1.02-2.15) | 1.49 (1.02-2.17) |
| P | 0.297 | 0.358 | 0.373 | 0.034 | 0.019 | 0.014 | 0.033 | 0.041 | 0.041 |
| Model A: Crude model Model B: Adjusted for age, sex Model C: Adjusted for age, sex, education, income level and physical activity | | | | | | | | | |

**Table S11-1 Association of SNPs with central obesity in different Fagerstrom Test for Nicotine Dependence (FTND) score groups before cessation in former smokers (additive model)**

|  | FTND 0 (n=416) | | | FTND 1-3 (n=489) | | | FTND ≥ 4 (n=328) | | |
| --- | --- | --- | --- | --- | --- | --- | --- | --- | --- |
|  | Model A | Model B | Model C | Model A | Model B | Model C | Model A | Model B | Model C |
| Rs6265 |  |  |  |  |  |  |  |  |  |
| A | 1 | 1 | 1 | 1 | 1 | 1 | 1 | 1 | 1 |
| G | 1.23 (0.92-1.64) | 1.27 (0.93-1.73) | 1.26 (0.93-1.72) | 1.40 (1.08-1.82) | 1.48 (1.13-1.94) | 1.49 (1.13-1.96) | 1.49 (1.10-2.03) | 1.52 (1.10-2.10) | 1.53 (1.10-2.12) |
| P | 0.159 | 0.129 | 0.140 | 0.011 | 0.005 | 0.005 | 0.011 | 0.010 | 0.011 |
| Rs4923457 |  |  |  |  |  |  |  |  |  |
| T | 1 | 1 | 1 | 1 | 1 | 1 | 1 | 1 | 1 |
| A | 1.20 (0.90-1.58) | 1.28 (0.95-1.73) | 1.28 (0.94-1.73) | 1.18 (0.92-1.53) | 1.21 (0.93-1.57) | 1.24 (0.95-1.61) | 1.38 (1.01-1.89) | 1.41 (1.02-1.95) | 1.40 (1.01-1.95) |
| P | 0.213 | 0.111 | 0.114 | 0.189 | 0.159 | 0.119 | 0.042 | 0.039 | 0.043 |
| Rs11030104 |  |  |  |  |  |  |  |  |  |
| G | 1 | 1 | 1 | 1 | 1 | 1 | 1 | 1 | 1 |
| A | 1.20 (0.90-1.61) | 1.26 (0.92-1.73) | 1.25 (0.91-1.72) | 1.47 (1.13-1.92) | 1.51 (1.15-1.99) | 1.55 (1.17-2.05) | 1.36 (0.99-1.86) | 1.34 (0.97-1.86) | 1.34 (0.96-1.86) |
| P | 0.220 | 0.149 | 0.162 | 0.005 | 0.003 | 0.002 | 0.054 | 0.074 | 0.082 |
| Rs6484320 |  |  |  |  |  |  |  |  |  |
| T | 1 | 1 | 1 | 1 | 1 | 1 | 1 | 1 | 1 |
| A | 1.20 (0.90-1.60) | 1.21 (0.89-1.64) | 1.20 (0.88-1.63) | 1.27 (0.98-1.65) | 1.31 (1.00-1.71) | 1.32 (1.00-1.73) | 1.40 (1.03-1.89) | 1.42 (1.04-1.95) | 1.42 (1.03-1.96) |
| P | 0.206 | 0.229 | 0.247 | 0.069 | 0.053 | 0.048 | 0.032 | 0.030 | 0.032 |
| Model A: Crude model Model B: Adjusted for age, sex Model C: Adjusted for age, sex, education, income level and physical activity | | | | | | | | | |

**Table S12 Odds ratios (ORs) (95%CI) of general obesity for different SNPs in different smoke exposure groups (excluding participants with body mass index (BMI) <18.5 (N=14,329)**

|  |  | Never smokers (n=12,230) | | |  | Former smokers (n=1,191) | | |  | Current smokers (n=908) | | |
| --- | --- | --- | --- | --- | --- | --- | --- | --- | --- | --- | --- | --- |
|  | General obesity‡ n(%) | Model A | Model B | Model C | General obesity‡ n(%) | Model A | Model B | Model C | General obesity‡ n(%) | Model A | Model B | Model C |
| Rs6265 | |  |  |  |  |  |  |  |  |  |  |  |
| AA | 365 (11.4) | 1 | 1 | 1 | 28 (8.8) | 1 | 1 | 1 | 19 (8.0) | 1 | 1 | 1 |
| GA | 763 (12.4) | 1.10  (0.96-1.26) | 1.10  (0.96-1.25) | 1.10 (0.96-1.25) | 55 (9.2) | 1.04  (0.65-1.68) | 1.07  (0.66-1.73) | 1.06  (0.66-1.72) | 34 (7.5) | 0.93  (0.52-1.67) | 0.90 (0.50-1.63) | 0.90 (0.50-1.62) |
| GG | 342 (12.0) | 1.06  (0.90-1.24) | 1.06  (0.90-1.24) | 1.06  (0.90-1.24) | 38 (14.0) | 1.68*  (1.00-2.82) | 1.74*  (1.04-2.94) | 1.74*  (1.04-2.94) | 20 (9.2) | 1.17  (0.60-2.25) | 1.11  (0.57-2.15) | 1.10  (0.57-2.14) |
| Rs4923457 | |  |  |  |  |  |  |  |  |  |  |  |
| TT | 346 (11.9) | 1 | 1 | 1 | 25 (8.3) | 1 | 1 | 1 | 14 (6.8) | 1 | 1 | 1 |
| AT | 737 (12.1) | 1.02  (0.89-1.17) | 1.02  (0.89-1.17) | 1.03  (0.90-1.18) | 54 (9.2) | 1.12  (0.68-1.84) | 1.13  (0.69-1.86) | 1.12  (0.68-1.84) | 37 (8.3 ) | 1.23  (0.65-2.33) | 1.23  (0.65-2.34) | 1.23  (0.65-2.35) |
| AA | 387 (11.9) | 1.01  (0.86-1.17) | 1.00  (0.86-1.17) | 1.01  (0.87-1.18) | 42 (13.8) | 1.77*  (1.05-2.30) | 1.85*  (1.09-3.12) | 1.85*  (1.09-3.12) | 22 (8.6) | 1.29  (0.64-2.59) | 1.27  (0.63-2.56) | 1.28  (0.63-2.59) |
| Rs11030104 | |  |  |  |  |  |  |  |  |  |  |  |
| GG | 368 (11.4) | 1 | 1 | 1 | 26 (8.5) | 1 | 1 | 1 | 20 (8.4) | 1 | 1 | 1 |
| AG | 771 (12.5) | 1.11  (0.97-1.27) | 1.11  (0.97-1.26) | 1.11  (0.97-1.27) | 59 (9.5) | 1.13  (0.70-1.83) | 1.13  (0.70-1.84) | 1.12  (0.69-1.82) | 32 (7.1) | 0.88  (0.49-1.57) | 0.82 (0.46-1.48) | 0.81  (0.45-1.46) |
| AA | 331 (11.6) | 1.02 (0.87-1.19) | 1.01 (0.87-1.19) | 1.02  (0.87-1.19) | 36 (13.6) | 1.70*  (1.00-2.90) | 1.75*  (1.02-3.00) | 1.75*  (1.03-3.00) | 21 (9.5) | 1.15  (0.61-2.18) | 1.09 (0.57-2.07) | 1.08  (0.56-2.07) |
| Rs6484320 | |  |  |  |  |  |  |  |  |  |  |  |
| TT | 355 (11.4) | 1 | 1 | 1 | 28 (9.0) | 1 | 1 | 1 | 18 (7.9) | 1 | 1 | 1 |
| TA | 771 (12.5) | 1.11  (0.97-1.27) | 1.11  (0.97-1.27) | 1.11  (0.97-1.27) | 56 (9.3) | 1.04  (0.64-1.67) | 1.05  (0.65-1.69) | 1.04 (0.65-1.68) | 35 (7.7) | 0.97  (0.54-1.76) | 0.96 (0.53-1.74) | 0.96  (0.53-1.75) |
| AA | 344 (11.6) | 1.01  (0.87-1.19) | 1.04  (0.87-1.19) | 1.02  (0.87-1.19) | 37 (13.2) | 1.53  (0.91-2.57) | 1.56  (0.93-2.63) | 1.56  (0.95-2.63) | 20 (8.9) | 1.14  (0.59-2.21) | 1.10  (0.56-2.15) | 1.10  (0.56-2.16) |
| ‡General obesity was defined as a body mass index (BMI) ≥ 28 kg/m2 using the criteria for the Asian population.  Model A: Crude model Model B: Adjusted for age, sex Model C: Adjusted for age, sex, education, income level and physical activity *P<0.05 | | | | | | | | | | | | |

**Table S13 Odds ratios (ORs) (95%CI) of general obesity for different SNPs in former smokers (grouped by years of smoking cessation)**

|  |  | Smoking cessation time ≤5 years (n=283) | | | | |
| --- | --- | --- | --- | --- | --- | --- |
|  | General obesity‡ n(%) | Model A | Model B | | Model C | |
| Rs6265 |  |  |  | |  | |
| AA | 7 (9.0) | 1 | 1 | | 1 | |
| GA | 16 (11.2) | 1.28 (0.50-3.25) | 1.33 (0.51-3.49) | | 1.34 (0.51-3.54) | |
| GG | 9 (14.5) | 1.72 (0.60-4.92) | 1.95 (0.66-5.75) | | 1.96 (0.66-5.81) | |
| Rs4923457 |  |  |  | |  | |
| TT | 7 (9.2) | 1 | 1 | | 1 | |
| AT | 15 (11.1) | 1.23 (0.48-3.17) | 1.21 (0.46-3.20) | | 1.23 (0.46-3.29) | |
| AA | 10 (13.9) | 1.59 (0.57-4.43) | 1.75 (0.61-5.04) | | 1.69 (0.59-4.90) | |
| Rs11030104 |  |  |  | |  | |
| GG | 6 (7.7) | 1 | 1 | | 1 | |
| AG | 18 (12.2) | 1.66 (0.63-4.37) | 1.71 (0.64-4.62) | | 1.76 (0.64-4.83) | |
| AA | 8 (14.0) | 1.96 (0.64-6.00) | 2.41 (0.76-7.64) | | 2.52 (0.79-8.07) | |
| Rs6484320 |  |  |  | |  | |
| TT | 7 (9.2) | 1 | 1 | | 1 | |
| TA | 16 (11.3) | 1.25 (0.49-3.19) | 1.25 (0.48-3.27) | | 1.36 (0.51-3.60) | |
| AA | 9 (13.8) | 1.58 (0.56-4.52) | 1.66 (0.56-4.86) | | 1.62 (0.55-4.80) | |
|  |  | Smoking cessation time > 5 years (n=950) | | | | |
| Rs6265 |  |  | |  | |  |
| AA | 21 (8.2) | 1 | | 1 | | 1 |
| GA | 39 (8.2) | 1.01 (0.58-1.74) | | 1.02 (0.58-1.77) | | 1.01 (0.58-1.76) |
| GG | 29 (13.4) | 1.74 (0.96-3.16) | | 1.77 (0.98-3.21) | | 1.77 (0.97-3.20) |
| Rs4923457 |  |  | |  | |  |
| TT | 18 (7.6) | 1 | | 1 | | 1 |
| AT | 39 (8.3) | 1.10 (0.62-1.97) | | 1.11 (0.62-1.99) | | 1.11 (0.62-1.98) |
| AA | 32 (13.3) | 1.87* (1.02-3.44) | | 1.92* (1.04-3.53) | | 1.91* (1.04-3.52) |
| Rs11030104 |  |  | |  | |  |
| GG | 20 (8.2) | 1 | | 1 | | 1 |
| AG | 41 (8.3) | 1.01 (0.58-1.77) | | 1.01 (0.58-1.77) | | 1.00 (0.57-1.79) |
| AA | 28 (13.2) | 1.70 (0.93-3.12) | | 1.72 (0.94-3.15) | | 1.71 (0.93-3.15) |
| Rs6484320 |  |  | |  | |  |
| TT | 21 (8.4) | 1 | | 1 | | 1 |
| TA | 40 (8.4) | 0.99 (0.57-1.72) | | 1.00 (0.58-1.74) | | 1.00 (0.57-1.73) |
| AA | 28 (12.6) | 1.56 (0.86-2.83) | | 1.57 (0.87-2.87) | | 1.58 (0.87-2.87) |

‡General obesity was defined as a body mass index (BMI) ≥ 28 kg/m2 using the criteria for the Asian population.

Model A: Crude model
Model B: Adjusted for age, sex
Model C: Adjusted for age, sex, education, income level and physical activity

*P<0.05

**Table S14 Relative risks (RRs) (95%CI) of new general obesity for different SNPs in former smokers**

| Smoking cessation time ≤5 years (n=256) | | | | | |
| --- | --- | --- | --- | --- | --- |
|  | General obesity‡ n(%) | | Model A | Model B | Model C |
| Rs6265 |  | |  |  |  |
| AA | 2 (2.7) | | 1 | 1 | 1 |
| GA | 6 (4.7) | | 1.72 (0.35-8.54) | 1.60 (0.32-7.98) | 1.57 (0.31-7.97) |
| GG | 3 (5.4) | | 1.96 (0.33-11.70) | 2.16 (0.36-12.95) | 2.18 (0.35-13.61) |
| Rs4923457 |  | |  |  |  |
| TT | 2 (2.8) | | 1 | 1 | 1 |
| AT | 5 (4.1) | | 1.46 (0.28-7.50) | 1.35 (0.26-7.02) | 1.30 (0.25-6.78) |
| AA | 4 (6.3) | | 2.25 (0.41-12.31) | 2.18 (0.40-11.93) | 2.17 (0.39-12.01) |
| Rs11030104 |  | |  |  |  |
| GG | 1 (1.4) | | 1 | 1 | 1 |
| AG | 7 (5.3) | | 3.82 (0.47-31.03) | 3.61 (0.44-29.41) | 3.40 (0.41-28.32) |
| AA | 3 (5.8) | | 4.15 (0.43-39.93) | 4.54 (0.47-43.77) | 4.83 (0.49-47.56) |
| Rs6484320 |  | |  |  |  |
| TT | 2 (2.8) | | 1 | 1 | 1 |
| TA | 5 (4.0) | | 1.42 (0.28-7.32) | 1.33 (0.26-6.85) | 1.37 (0.26-7.28) |
| AA | 4 (6.7) | | 2.37 (0.43-12.92) | 2.26 (0.41-12.36) | 2.16 (0.39-12.02) |
| Smoking cessation time > 5 years (n=871) | | | | | |
| Rs6265 | |  |  |  |  |
| AA | | 9 (3.8) | 1 | 1 | 1 |
| GA | | 11 (2.5) | 0.66 (0.28-1.60) | 0.66 (0.27-1.59) | 0.69 (0.29-1.68) |
| GG | | 8 (4.2) | 1.13 (0.44-2.93) | 1.12 (0.43-2.90) | 1.12 (0.43-2.91) |
| Rs4923457 | |  |  |  |  |
| TT | | 9 (4.0) | 1 | 1 | 1 |
| AT | | 10 (2.3) | 0.58 (0.23-1.42) | 0.57 (0.23-1.41) | 0.60 (0.24-1.47) |
| AA | | 9 (4.2) | 1.06 (0.41-2.67) | 1.04 (0.41-2.63) | 1.04 (0.41-2.63) |
| Rs11030104 | |  |  |  |  |
| GG | | 8 (3.5) | 1 | 1 | 1 |
| AG | | 12 (2.6) | 0.75 (0.31-1.83) | 0.76 (0.31-1.85) | 0.80 (0.33-1.96) |
| AA | | 8 (4.3) | 1.23 (0.46-3.27) | 1.22 (0.46-3.26) | 1.22 (0.46-3.26) |
| Rs6484320 | |  |  |  |  |
| TT | | 9 (3.9) | 1 | 1 | 1 |
| TA | | 11 (2.5) | 0.64 (0.27-1.55) | 0.64 (0.27-1.55) | 0.68 (0.28-1.64) |
| AA | | 8 (4.1) | 1.05 (0.40-2.71) | 1.04 (0.40-2.70) | 1.05 (0.41-2.72) |
| Former smokers (n=1,127) | | | | | |
| Rs6265 |  | |  |  |  |
| AA | 11 (3.5) | | 1 | 1 | 1 |
| GA | 17 (3.0) | | 0.85 (0.40-1.82) | 0.85 (0.40-1.82) | 0.86 (0.40-1.84) |
| GG | 11 (4.5) | | 1.28 (0.55-2.95) | 1.29 (0.56-2.97) | 1.29 (0.56-2.97) |
| Rs4923457 |  | |  |  |  |
| TT | 11 (3.7) | | 1 | 1 | 1 |
| AT | 15 (2.7) | | 0.73 (0.33-1.58) | 0.73 (0.33-1.59) | 0.73 (0.33-1.58) |
| AA | 13 (4.7) | | 1.27 (0.57-2.84) | 1.28 (0.57-2.87) | 1.28 (0.57-2.86) |
| Rs11030104 |  | |  |  |  |
| GG | 9 (3.0) | | 1 | 1 | 1 |
| AG | 19 (3.2) | | 1.08 (0.49-2.38) | 1.07 (0.49-2.37) | 1.07 (0.48-2.38) |
| AA | 11 (4.6) | | 1.54 (0.64-3.72) | 1.55 (0.64-3.74) | 1.55 (0.64-3.75) |
| Rs6484320 |  | |  |  |  |
| TT | 11 (3.6) | | 1 | 1 | 1 |
| TA | 16 (2.8) | | 0.78 (0.36-1.68) | 0.78 (0.36-1.68) | 0.78 (0.36-1.68) |
| AA | 12 (4.7) | | 1.29 (0.57-2.92) | 1.29 (0.57-2.92) | 1.29 (0.57-2.91) |

‡General obesity was defined as a body mass index (BMI) ≥ 28 kg/m^2^ using the criteria for the Asian population.

Model A: Crude model
Model B: Adjusted for age, sex
Model C: Adjusted for age, sex, education, income level and physical activity

**Table S15 Odds raios (ORs) (95%CI) of central obesity for different SNPs in former smokers (grouped by years of smoking cessation)**

|  |  | Smoking cessation time ≤5 years (n=283) | | | | |
| --- | --- | --- | --- | --- | --- | --- |
|  | Central obesity‡ n(%) | Model A | Model B | | Model C | |
| Rs6265 |  |  |  | |  | |
| AA | 22 (28.2) | 1 | 1 | | 1 | |
| GA | 63 (44.1) | 2.01* (1.11-3.63) | 2.14* (1.16-3.98) | | 2.10* (1.13-3.92) | |
| GG | 31 (50.0) | 2.55* (1.26-5.13) | 2.94* (1.42-6.09) | | 2.89* (1.39-5.99) | |
| Rs4923457 |  |  |  | |  | |
| TT | 26 (34.2) | 1 | 1 | | 1 | |
| AT | 58 (43.0) | 1.45 (0.81-2.60) | 1.43 (0.78-2.62) | | 1.40 (0.76-2.58) | |
| AA | 32 (44.4) | 1.54 (0.79-2.99) | 1.68 (0.84-3.33) | | 1.66 (0.84-3.30) | |
| Rs11030104 |  |  |  | |  | |
| GG | 24 (30.8) | 1 | 1 | | 1 | |
| AG | 62 (41.9) | 1.62 (0.91-2.90) | 1.68 (0.92-3.08) | | 1.64 (0.89-3.02) | |
| AA | 30 (52.6) | 2.50* (1.23-5.08) | 3.02* (1.44-6.31) | | 2.97* (1.42-6.22) | |
| Rs6484320 |  |  |  | |  | |
| TT | 24 (31.6) | 1 | 1 | | 1 | |
| TA | 61 (43.0) | 1.63 (0.91-2.93) | 1.66 (0.90-3.03) | | 1.62 (0.87-2.99) | |
| AA | 31 (47.7) | 1.98 (1.00-3.92) | 2.09* (1.03-4.25) | | 2.05* (1.01-4.17) | |
|  |  | Smoking cessation time > 5 years (n=950) | | | | |
| Rs6265 |  |  | |  | |  |
| AA | 89 (34.6) | 1 | | 1 | | 1 |
| GA | 184 (38.6) | 1.19 (0.86-1.63) | | 1.22 (0.88-1.70) | | 1.23 (0.88-1.72) |
| GG | 103 (47.7) | 1.72* (1.19-2.49) | | 1.85* (1.26-2.73) | | 1.86* (1.26-2.75) |
| Rs4923457 |  |  | |  | |  |
| TT | 82 (34.5) | 1 | | 1 | | 1 |
| AT | 186 (39.5) | 1.24 (0.90-1.72) | | 1.31 (0.93-1.84) | | 1.33 (0.94-1.88) |
| AA | 108 (44.8) | 1.55* (1.07-2.23) | | 1.72* (1.17-2.53) | | 1.74* (1.18-2.57) |
| Rs11030104 |  |  | |  | |  |
| GG | 85 (34.8) | 1 | | 1 | | 1 |
| AG | 191 (38.7) | 1.18 (0.86-1.62) | | 1.14 (0.82-1.60) | | 1.16 (0.83-1.63) |
| AA | 100 (47.2) | 1.67* (1.15-2.44) | | 1.75* (1.18-2.59) | | 1.77* (1.19-2.62) |
| Rs6484320 |  |  | |  | |  |
| TT | 86 (34.5) | 1 | | 1 | | 1 |
| TA | 189 (39.5) | 1.24 (0.90-1.71) | | 1.26 (0.90-1.77) | | 1.28 (0.91-1.79) |
| AA | 101 (45.3) | 1.57* (1.08-2.27) | | 1.64* (1.11-2.43) | | 1.65* (1.12-2.44) |

‡Central obesity was based on the criteria for the Asian population (waist circumference [WC] ≥ 90 cm in men and ≥ 80 cm in women)

Model A: Crude model
Model B: Adjusted for age, sex
Model C: Adjusted for age, sex, education, income level and physical activity

*P<0.05

**Table S16 Relative risks (RRs) (95%CI) of new central obesity for different SNPs in former smokers**

| Smoking cessation time ≤5 years (n=205) | | | | | | | |
| --- | --- | --- | --- | --- | --- | --- | --- |
|  | Central obesity‡ n(%) | Model A | Model B | | | Model C | |
| Rs6265 |  |  |  | | |  | |
| AA | 9 (14.1) | 1 | 1 | | | 1 | |
| GA | 30 (29.4) | 2.09 (0.99-4.41) | 2.22* (1.05-4.70) | | | 2.18* (1.03-4.61) | |
| GG | 11 (28.2) | 2.01 (0.83-4.84) | 2.02 (0.84-4.88) | | | 2.00 (0.83-4.83) | |
| Rs4923457 |  |  |  | | |  | |
| TT | 11 (18.3) | 1 | 1 | | | 1 | |
| AT | 29 (29.0) | 1.58 (0.79-3.17) | 1.67 (0.83-3.36) | | | 1.62 (0.80-3.26) | |
| AA | 10 (22.2) | 1.21 (0.52-2.85) | 1.24 (0.52-2.92) | | | 1.24 (0.52-2.93) | |
| Rs11030104 |  |  |  | | |  | |
| GG | 10 (16.1) | 1 | 1 | | | 1 | |
| AG | 29 (26.9) | 1.67 (0.81-3.42) | 1.76 (0.85-3.61) | | | 1.70 (0.82-3.51) | |
| AA | 11 (31.4) | 1.95 (0.83-4.59) | 2.07 (0.88-4.87) | | | 1.99 (0.84-4.71) | |
| Rs6484320 |  |  |  | | |  | |
| TT | 10 (16.4) | 1 | 1 | | | 1 | |
| TA | 28 (27.5) | 1.68 (0.81-3.45) | 1.69 (0.82-3.49) | | | 1.61 (0.78-3.34) | |
| AA | 12 (28.6) | 1.74 (0.75-4.23) | 1.72 (0.74-3.98) | | | 1.67 (0.72-3.88) | |
| Smoking cessation time > 5 years (n=691) | | | | | | | |
| Rs6265 |  |  |  | |  | | |
| AA | 39 (20.2) | 1 | 1 | | 1 | | |
| GA | 73 (20.9) | 1.03 (0.70-1.52) | 1.05 (0.71-1.55) | | 1.05 (0.71-1.55) | | |
| GG | 43 (29.1) | 1.44 (0.93-2.22) | 1.49 (0.96-2.30) | | 1.49 (0.96-2.29) | | |
| Rs4923457 |  |  |  | |  | | |
| TT | 38 (20.9) | 1 | 1 | | 1 | | |
| AT | 78 (22.5) | 1.08 (0.73-1.59) | 1.09 (0.74-1.61) | | 1.11 (0.75-1.63) | | |
| AA | 39 (24.1) | 1.15 (0.74-1.80) | 1.21 (0.77-1.89) | | 1.22 (0.78-1.91) | | |
| Rs11030104 |  |  |  | |  | | |
| GG | 37 (20.2) | 1 | 1 | | 1 | | |
| AG | 77 (21.2) | 1.08 (0.71-1.55) | 1.02 (0.69-1.50) | | 1.02 (0.69-1.51) | | |
| AA | 41 (28.3) | 1.40 (0.90-2.18) | 1.42 (0.91-2.21) | | 1.42 (0.91-2.22) | | |
| Rs6484320 |  |  |  | |  | | |
| TT | 37 (19.9) | 1 | 1 | | 1 | | |
| TA | 78 (22.2) | 1.12 (0.76-1.65) | 1.12 (0.76-1.65) | | 1.11 (0.75-1.65) | | |
| AA | 40 (26.0) | 1.31 (0.84-2.04) | 1.34 (0.86-2.09) | | 1.33 (0.85-2.08) | | |
| Former smokers (n=896) | | | | | | | |
| Rs6265 |  |  | |  | | |  |
| AA | 48 (18.7) | 1 | | 1 | | | 1 |
| GA | 103 (22.8) | 1.22 (0.87-1.72) | | 1.24 (0.88-1.75) | | | 1.25 (0.89-1.76) |
| GG | 54 (28.9) | 1.55* (1.05-2.28) | | 1.59* (1.08-2.34) | | | 1.59* (1.08-2.34) |
| Rs4923457 |  |  | |  | | |  |
| TT | 49 (20.2) | 1 | | 1 | | | 1 |
| AT | 107 (23.9) | 1.18 (0.84-1.66) | | 1.19 (0.85-1.67) | | | 1.20 (0.86-1.69) |
| AA | 49 (23.7) | 1.17 (0.79-1.74) | | 1.20 (0.81-1.79) | | | 1.21 (0.81-1.79) |
| Rs11030104 |  |  | |  | | |  |
| GG | 47 (19.2) | 1 | | 1 | | | 1 |
| AG | 106 (22.5) | 1.17 (0.83-1.65) | | 1.15 (0.82-1.62) | | | 1.16 (0.82-1.63) |
| AA | 52 (28.9) | 1.51* (1.02-2.23) | | 1.53* (1.03-2.27) | | | 1.54* (1.04-2.28) |
| Rs6484320 |  |  | |  | | |  |
| TT | 47 (19.0) | 1 | | 1 | | | 1 |
| TA | 106 (23.4) | 1.23 (0.87-1.73) | | 1.23 (0.87-1.73) | | | 1.23 (0.87-1.73) |
| AA | 52 (26.5) | 1.39 (0.94-2.07) | | 1.41 (0.95-2.09) | | | 1.41 (0.95-2.09) |

‡Central obesity was based on the criteria for the Asian population (waist circumference [WC] ≥ 90 cm in men and ≥ 80 cm in women)

Model A: Crude model
Model B: Adjusted for age, sex
Model C: Adjusted for age, sex, education, income level and physical activity

*P<0.05

**Table S17** **Odds Ratios (ORs) (95%CI) of general obesity‡ for different SNPs in different smoke exposure groups (including participants whose weight changed significantly since 18 years old only, N=11,891)**

|  |  | Never smokers (n=10,176) | | |  | Former smokers (n=972) | | |  | Current smokers (n=743) | | |
| --- | --- | --- | --- | --- | --- | --- | --- | --- | --- | --- | --- | --- |
|  | Cases | Model A | Model B | Model C | Cases | Model A | Model B | Model C | Cases | Model A | Model B | Model C |
| Rs6265 | |  |  |  |  |  |  |  |  |  |  |  |
| AA | 338 (12.7) | 1 | 1 | 1 | 26 (10.0) | 1 | 1 | 1 | 16 (8.1) | 1 | 1 | 1 |
| GA | 719 (14.0) | 1.12  (0.98-1.29) | 1.12 (0.98-1.29) | 1.13 (0.98-1.30) | 51 (10.6) | 1.07  (0.65-1.76) | 1.09  (0.66-1.80) | 1.09  (0.66-1.80) | 31 (8.4) | 1.04  (0.55-1.95) | 1.01  (0.54-1.90) | 1.00  (0.53-1.88) |
| GG | 322 (13.5) | 1.08  (0.92-1.27) | 1.08  (0.92-1.27) | 1.08  (0.92-1.28) | 38 (16.4) | 1.76* (1.03-3.01) | 1.84*  (1.07-3.15) | 1.83*  (1.07-3.14) | 19 (10.9) | 1.39  (0.69-2.79) | 1.33  (0.66-2.69) | 1.32  (0.65-2.68) |
| Rs4923457 | |  |  |  |  |  |  |  |  |  |  |  |
| TT | 319 (13.2) | 1 | 1 | 1 | 23 (9.1) | 1 | 1 | 1 | 11 (6.4) | 1 | 1 | 1 |
| AT | 694 (13.7) | 1.04  (0.90-1.20) | 1.04  (0.90-1.20) | 1.05  (0.91-1.21) | 50 (10.8) | 1.21  (0.72-2.04) | 1.22 (0.72-2.05) | 1.22 (0.72-2.05) | 34 (9.4 ) | 1.51  (0.75-3.06) | 1.51  (0.74-3.06) | 1.50 (0.74-3.63) |
| AA | 366 (13.6) | 1.03  (0.88-1.22) | 1.03  (0.88-1.22) | 1.04  (0.89-1.23) | 42 (16.5) | 1.99*  (1.16-3.42) | 2.10*  (1.22-3.63) | 2.10*  (1.22-3.63) | 21 (10.1) | 1.64  (0.77-3.51) | 1.61 (0.75-3.45) | 1.63  (0.76-3.52) |
| Rs11030104 | |  |  |  |  |  |  |  |  |  |  |  |
| GG | 338 (12.6) | 1 | 1 | 1 | 24 (9.6) | 1 | 1 | 1 | 17 (8.5) | 1 | 1 | 1 |
| AG | 729 (14.2) | 1.15  (1.00-1.32) | 1.15  (1.00-1.32) | 1.15*  (1.00-1.32) | 55 (11.1) | 1.17 (0.71-1.94) | 1.17  (0.70-1.94) | 1.16 (0.70-1.94) | 29 (8.0) | 0.94  (0.50-1.76) | 0.92  (0.49-1.72) | 0.90  (0.48-1.69) |
| AA | 312 (13.2) | 1.06  (0.89-1.24) | 1.05  (0.89-1.24) | 1.06  (0.90-1.25) | 36 (15.9) | 1.77*  (1.02-3.07) | 1.84*  (1.05-3.20) | 1.83*  (1.05-3.20) | 20 (11.0) | 1.33  (0.67-2.62) | 1.28  (0.65-2.54) | 1.28  (0.64-2.55) |
| Rs6484320 | |  |  |  |  |  |  |  |  |  |  |  |
| TT | 327 (12.6) | 1 | 1 | 1 | 26 (10.3) | 1 | 1 | 1 | 15 (7.8) | 1 | 1 | 1 |
| TA | 727 (14.2) | 1.15 (1.00-1.32) | 1.15  (1.00-1.32) | 1.15*  (1.00-1.33) | 52 (10.8) | 1.06  (0.64-1.74) | 1.07  (0.65-1.77) | 1.07  (0.65-1.77) | 32 (8.7) | 1.12  (0.59-2.12) | 1.10  (0.58-2.08) | 1.09  (0.57-2.08) |
| AA | 325 (13.1) | 1.05  (0.89-1.23) | 1.05  (0.89-1.23) | 1.05  (0.89-1.24) | 37 (15.5) | 1.61  (0.94-2.75) | 1.66 (0.97-2.85) | 1.65  (0.96-2.84) | 19 (10.4) | 1.38  (0.68-2.80) | 1.34  (0.66-2.74) | 1.34  (0.65-2.75) |
| ‡General obesity was defined as a body mass index (BMI) ≥ 28 kg/m2 using the criteria for the Asian population.  Model A: Crude model Model B: Adjusted for age, sex Model C: Adjusted for age, sex, education, income level and physical activity *P<0.05 | | | | | | | | | | | | |

**Table S18 Odds ratios (ORs) (95%CI) of central obesity‡ for different SNPs in different smoke exposure groups (including participants whose waist circumference changed significantly since 18 years old only, N=11,485)**

|  | Cases | Model A | Model B | Model C |
| --- | --- | --- | --- | --- |
| Never smokers (n=9,867) | | | | |
| Rs6265 |  |  |  |  |
| AA | 1,547 (59.6) | 1 | 1 | 1 |
| GA | 3,088 (62.2) | 1.12*  (1.01-1.23) | 1.11*  (1.01-1.23) | 1.11*  (1.01-1.23) |
| GG | 1,477 (63.9) | 1.20*  (1.07-1.35) | 1.19*  (1.06-1.34) | 1.19*  (1.06-1.34) |
| Rs4923457 |  |  |  |  |
| TT | 1,435 (60.9) | 1 | 1 | 1 |
| AT | 3,021 (61.7) | 1.03  (0.94-1.14) | 1.03  (0.93-1.14) | 1.04  (0.93-1.15) |
| AA | 1,656 (63.5) | 1.12  (0.99-1.25) | 1.10  (0.98-1.24) | 1.11  (0.98-1.25) |
| Rs11030104 |  |  |  |  |
| GG | 1,559 (60.0) | 1 | 1 | 1 |
| AG | 3,088 (62.1) | 1.09  (0.99-1.20) | 1.09  (0.98-1.20) | 1.09  (0.98-1.20) |
| AA | 1,465 (63.8) | 1.15*  (1.05-1.27) | 1.16*  (1.03-1.31) | 1.16*  (1.03-1.31) |
| Rs6484320 |  |  |  |  |
| TT | 1,508 (59.8) | 1 | 1 | 1 |
| TA | 3,086 (62.4) | 1.11*  (1.01-1.23) | 1.11  (1.00-1.23) | 1.11  (1.00-1.22) |
| AA | 1,518 (63.3) | 1.16*  (1.03-1.30) | 1.15*  (1.02-1.29) | 1.15*  (1.02-1.29) |
| Former smokers (n=905) | | | | |
| Rs6265 |  |  |  |  |
| AA | 96 (40.7) | 1 | 1 | 1 |
| GA | 214 (47.6) | 1.32  (0.96-1.82) | 1.33  (0.96-1.85) | 1.33  (0.96-1.86) |
| GG | 121 (51.3) | 1.80 * (1.24-2.61) | 1.93*  (1.31-2.83) | 1.93* (1.31-2.84) |
| Rs4923457 |  |  |  |  |
| TT | 93 (41.3) | 1 | 1 | 1 |
| AT | 211 (48.0) | 1.31  (0.95-1.81) | 1.31  (0.94-1.84) | 1.31  (0.94-1.84) |
| AA | 127 (52.9) | 1.60*  (1.11-2.30) | 1.72*  (1.17-2.51) | 1.72*  (1.17-2.51) |
| Rs11030104 |  |  |  |  |
| GG | 94 (42.2) | 1 | 1 | 1 |
| AG | 220 (47.0) | 1.22  (0.88-1.68) | 1.18  (0.84-1.65) | 1.19  (0.85-1.66) |
| AA | 117 (54.7) | 1.66*  (1.13-2.42) | 1.74* (1.18-2.58) | 1.76* (1.19-2.60) |
| Rs6484320 |  |  |  |  |
| TT | 96 (42.1) | 1 | 1 | 1 |
| TA | 217 (48.0) | 1.27  (0.92-1.75) | 1.27  (0.91-1.77) | 1.28  (0.91-1.78) |
| AA | 118 (52.4) | 1.52*  (1.05-2.20) | 1.57*  (1.07-2.31) | 1.58*  (1.07-2.31) |
| Current smokers (n=713) | | | | |
| Rs6265 |  |  |  |  |
| AA | 73 (39.2) | 1 | 1 | 1 |
| GA | 118 (34.2) | 0.81  (0.56-1.16) | 0.75  (0.52-1.10) | 0.76  (0.52-1.11) |
| GG | 71 (39.0) | 0.99  (0.65-1.51) | 0.91  (0.59-1.39) | 0.92  (0.60-1.43) |
| Rs4923457 |  |  |  |  |
| TT | 59 (36.6) | 1 | 1 | 1 |
| AT | 122 (35.8 ) | 0.96  (0.65-1.42) | 0.96  (0.64-1.43) | 0.96  (0.64-1.43) |
| AA | 81 (38.4) | 1.08  (0.71-1.65) | 1.03  (0.66-1.59) | 1.04  (0.67-1.61) |
| Rs11030104 |  |  |  |  |
| GG | 73 (39.0) | 1 | 1 | 1 |
| AG | 117 (34.6) | 0.83 (0.57-1.20) | 0.79  (0.54-1.15) | 0.79  (0.54-1.16) |
| AA | 72 (38.3) | 0.97 (0.64-1.47) | 0.91 (0.59-1.39) | 0.93 (0.60-1.43) |
| Rs6484320 |  |  |  |  |
| TT | 74 (41.3) | 1 | 1 | 1 |
| TA | 116 (33.5) | 0.72  (0.49-1.04) | 0.69  (0.47-1.01) | 0.69  (0.47-1.01) |
| AA | 72 (38.3) | 0.88  (0.58-1.34) | 0.82  (0.54-1.27) | 0.84  (0.54-1.30) |
| ‡Central obesity was based on the criteria for the Asian population (waist circumference [WC] ≥ 90 cm in men and ≥ 80 cm in women)  Model A: Crude model Model B: Adjusted for age, sex Model C: Adjusted for age, sex, education, income level and physical activity  *P<0.05 | | | | |

**Table S19 Odds ratios (ORs) (95%CI) of central obesity‡ for different SNPs in different smoke exposure groups (including male participants whose waist circumference changed significantly since 18 years old only, N=2,299)**

|  |  | Never smokers (n=925) | | |  | Former smokers (n=756) | | |  | Current smokers (n=618) | | |
| --- | --- | --- | --- | --- | --- | --- | --- | --- | --- | --- | --- | --- |
|  | Cases | Model A | Model B | Model C | Cases | Model A | Model B | Model C | Cases | Model A | Model B | Model C |
| Rs6265 |  |  |  |  |  |  |  |  |  |  |  |  |
| AA | 85 (33.2) | 1 | 1 | 1 | 68 (34.3) | 1 | 1 | 1 | 60 (29.9) | 1 | 1 | 1 |
| GA | 163 (35.2) | 1.09  (0.79-1.51) | 1.09  (0.79-1.51) | 1.09  (0.79-1.51) | 154 (41.5) | 1.36  (0.95-1.94) | 1.36 (0.95-1.96) | 1.37  (0.96-1.97) | 87 (29.3) | 0.75  (0.50-1.12) | 0.75 (0.50-1.12) | 0.75 (0.50-1.13) |
| GG | 78 (37.9) | 1.23  (0.84-1.80) | 1.23  (0.84-1.80) | 1.24  (0.84-1.81) | 96 (51.3) | 2.02*  (1.34-3.04) | 2.03*  (1.35-3.07) | 2.04*  (1.35-3.09) | 54 (35.3) | 0.98  (0.62-1.55) | 0.98  (0.62-1.55) | 1.00  (0.63-1.60) |
| Rs4923457 |  |  |  |  |  |  |  |  |  |  |  |  |
| TT | 79 (35.1) | 1 | 1 | 1 | 66 (35.1) | 1 | 1 | 1 | 45 (31.7) | 1 | 1 | 1 |
| AT | 165 (35.0) | 1.00  (0.71-1.39) | 1.00  (0.71-1.39) | 0.99 (0.71-1.39) | 153 (42.3) | 1.35  (0.94-1.95) | 1.36  (0.94-1.95) | 1.36  (0.94-1.96) | 94 (31.5 ) | 0.99 (0.65-1.53) | 1.00  (0.65-1.54) | 1.00  (0.65-1.55) |
| AA | 82 (35.8) | 1.03  (0.70-1.51) | 1.03  (0.70-1.51) | 1.04  (0.70-1.52) | 99 (48.1) | 1.71*  (1.14-2.57) | 1.72*  (1.15-2.59) | 1.72*  (1.15-2.59) | 62 (34.8) | 1.15  (0.72-1.84) | 1.15  (0.72-1.85) | 1.17  (0.73-1.87) |
| Rs11030104 |  |  |  |  |  |  |  |  |  |  |  |  |
| GG | 86 (33.6) | 1 | 1 | 1 | 68 (35.8) | 1 | 1 | 1 | 59 (29.4) | 1 | 1 | 1 |
| AG | 164 (35.3) | 1.08  (0.78-1.49) | 1.08  (0.78-1.49) | 1.07  (0.78-1.48) | 156 (40.8) | 1.24  (0.86-1.78) | 1.24  (0.87-1.78) | 1.26  (0.88-1.81) | 88 (30.1) | 0.79  (0.53-1.18) | 0.79  (0.53-1.19) | 0.80 (0.53-1.20) |
| AA | 76 (37.3) | 1.17 (0.80-1.72) | 1.17 (0.80-1.72) | 1.18 (0.81-1.74) | 94 (51.1) | 1.87*  (1.24-2.84) | 1.89*  (1.25-2.86) | 1.91*  (1.26-2.90) | 54 (34.0) | 0.94  (0.60-1.49) | 0.94  (0.60-1.49) | 0.97  (0.61-1.53) |
| Rs6484320 |  |  |  |  |  |  |  |  |  |  |  |  |
| TT | 79 (32.5) | 1 | 1 | 1 | 69 (35.9) | 1 | 1 | 1 | 59 (37.1) | 1 | 1 | 1 |
| TA | 165 (35.1) | 1.12  (0.81-1.56) | 1.12  (0.81-1.56) | 1.12  (0.80-1.55) | 157 (42.0) | 1.29  (0.90-1.85) | 1.29  (0.90-1.85) | 1.30  (0.91-1.87) | 87 (29.0) | 0.69  (0.46-1.04) | 0.69  (0.46-1.05) | 0.70  (0.47-1.06) |
| AA | 82 (38.7) | 1.31  (0.89-1.93) | 1.31  (0.89-1.93) | 1.31  (0.89-1.93) | 92 (48.4) | 1.67*  (1.11-2.52) | 1.68*  (1.12-2.54) | 1.69*  (1.12-2.55) | 55 (34.6) | 0.90  (0.57-1.42) | 0.89  (0.56-1.41) | 0.91  (0.57-1.45) |
| ‡Central obesity was based on the criteria for the Asian population (waist circumference [WC] ≥ 90 cm in men and ≥ 80 cm in women)  Model A: Crude model Model B: Adjusted for age Model C: Adjusted for age, education, income level and physical activity  *P<0.05 | | | | | | | | | | | | |

**Table S20 Odds ratios (ORs) (95%CI) of general obesity and central obesity^‡^ for different SNPs in former smokers (excluding participants who quit smoking because of diseases, N=746)**

|  | Cases | Model A | Model B | Model C |
| --- | --- | --- | --- | --- |
| **General obesity** | | | | |
| Rs6265 |  |  |  |  |
| AA | 15 (7.3) | 1 | 1 | 1 |
| GA | 39 (10.4) | 1.48 (0.79-2.75) | 1.56 (0.83-2.92) | 1.53 (0.81-2.86) |
| GG | 22 (13.3) | 1.96 (0.98-3.91) | 2.01 (1.00-4.04) | 1.99 (0.99-4.01) |
| Rs4923457 |  |  |  |  |
| TT | 15 (7.7) | 1 | 1 | 1 |
| AT | 35 (9.6) | 1.27 (0.68-2.39) | 1.27 (0.68-2.40) | 1.23 (0.65-2.32) |
| AA | 26 (14.0) | 1.95 (1.00-3.81) | 1.98 (1.01-3.89) | 1.95 (0.99-3.83) |
| Rs11030104 |  |  |  |  |
| GG | 13 (6.6) | 1 | 1 | 1 |
| AG | 42 (10.8) | 1.70 (0.89-3.25) | 1.75 (0.91-3.35) | 1.70 (0.89-3.27) |
| AA | 21 (13.1) | 2.13 (1.03-4.40) | 2.20 (1.06-4.56) | 2.18 (1.05-4.53) |
| Rs6484320 |  |  |  |  |
| TT | 15 (7.5) | 1 | 1 | 1 |
| TA | 39 (10.4) | 1.44 (0.77-2.67) | 1.48 (0.79-2.76) | 1.44 (0.77-2.70) |
| AA | 22 (12.8) | 1.81 (0.91-3.61) | 1.83 (0.91-3.66) | 1.81 (0.90-3.63) |
| **Central obesity** | | | | |
| Rs6265 |  |  |  |  |
| AA | 69 (33.5) | 1 | 1 | 1 |
| GA | 153 (40.8) | 1.37 (0.96-1.95) | 1.46 (1.00-2.13) | 1.47 (1.01-2.14) |
| GG | 77 (46.7) | 1.74 (1.14-2.65) | 1.90 (1.22-2.96) | 1.91 (1.23-2.99) |
| Rs4923457 |  |  |  |  |
| TT | 66 (33.8) | 1 | 1 | 1 |
| AT | 150 (41.1) | 1.36 (0.95-1.96) | 1.36 (0.93-2.00) | 1.37 (0.93-2.01) |
| AA | 83 (44.6) | 1.58 (1.04-2.38) | 1.65 (1.07-2.55) | 1.65 (1.07-2.55) |
| Rs11030104 |  |  |  |  |
| GG | 66 (33.7) | 1 | 1 | 1 |
| AG | 158 (40.5) | 1.34 (0.94-1.92) | 1.37 (0.94-2.00) | 1.37 (0.94-2.01) |
| AA | 75 (46.9) | 1.74 (1.13-2.67) | 1.90 (1.21-2.99) | 1.92 (1.22-3.02) |
| Rs6484320 |  |  |  |  |
| TT | 67 (33.5) | 1 | 1 | 1 |
| TA | 155 (41.4) | 1.41 (0.98-2.01) | 1.47 (1.00-2.14) | 1.47 (1.00-2.15) |
| AA | 77 (44.8) | 1.61 (1.06-2.45) | 1.70 (1.09-2.64) | 1.70 (1.09-2.65) |

‡General obesity was defined as a body mass index (BMI) ≥ 28 kg/m2 using the criteria for the Asian population; Central obesity was based on the criteria for the Asian population (waist circumference [WC] ≥ 90 cm in men and ≥ 80 cm in women)

Model A: Crude model
Model B: Adjusted for age
Model C: Adjusted for age, education, income level and physical activity

*P<0.05

**Table S21 Characteristics of the participants with or without genetic data**

|  | Without genetic data | With genetic data | P | Excluded from this study | Included in this study |  |
| --- | --- | --- | --- | --- | --- | --- |
|  | (n=14,053) | (n=16,465) |  | (n=1,393) | (n=15,072) |  |
| Mean(SD) |  |  |  |  |  |  |
| **Age(years)** | 63.2 (7.2) | 61.0 (6.9) | <0.001 | 61.0 (6.9) | 61.0 (6.9) | 0.66 |
| **Body Mass Index** | 23.8 (3.4) | 23.8 (3.3) | 0.37 | 23.7 (3.2) | 23.8 (3.3) | 0.385 |
| n (%) |  |  |  |  |  |  |
| **Gender** |  |  | <0.001 |  |  | <0.001 |
| Female | 9,232 (65.7) | 12,835 (78.0) |  | 932 (66.9) | 11,903 (79.0) |  |
| Male | 4,821 (34.3) | 3,630 (22.0) |  | 461 (33.1) | 3,169 (21.0) |  |
| **Education** |  |  | <0.001 |  |  | 0.06 |
| <=primary | 6,547 (46.7) | 6,540 (39.7) |  | 515 (37.1) | 6,025 (40.0) |  |
| middle school | 6,177 (44.0) | 8,501 (51.6) |  | 739 (53.2) | 7,762 (51.5) |  |
| >=college | 1,310 (9.3) | 1,421 (8.6) |  | 136 (9.8) | 1,285 (8.5) |  |
| **Income (Yuan,**  **US$1=6.88Yuan)** |  |  | <0.001 |  |  | 0.21 |
| <10,000 | 4,796 (34.2) | 5,356 (32.5) |  | 421 (30.3) | 4,935 (32.7) |  |
| 10000-15,000 | 5,788 (41.2) | 7,336 (44.6) |  | 625 (45.0) | 6,711 (44.5) |  |
| ≥15,000 | 2,745 (19.6) | 2,998 (18.2) |  | 275 (19.8) | 2,723 (18.1) |  |
| don't know | 707 (5.0) | 770 (4.7) |  | 67 (4.8) | 703 (4.7) |  |
| **Exercise(IPAQ)** |  |  | <0.001 |  |  | 0.08 |
| Low | 1,223 (8.7) | 1,337 (8.1) |  | 100 (7.2) | 1,237 (8.2) |  |
| Moderate | 6,064 (43.2) | 6,394 (38.8) |  | 516 (37.0) | 5,878 (39.0) |  |
| High | 6,766 (48.1) | 8,734 (53.0) |  | 777 (55.8) | 7,957 (52.8) |  |

IPAQ = International Physical Activity Questionnaire (short-form)

**Table S22 Odds ratios (95%CI) of general obesity for different SNPs in different smoke exposure groups (additive model)**

|  | Never smokers (n=12,852) | | | Former smokers (n=1,233) | | | Current smokers (n=987) | | |
| --- | --- | --- | --- | --- | --- | --- | --- | --- | --- |
|  | Model A | Model B | Model C | Model A | Model B | Model C | Model A | Model B | Model C |
| Rs6265 |  |  |  |  |  |  |  |  |  |
| A | 1 | 1 | 1 | 1 | 1 | 1 | 1 | 1 | 1 |
| G | 1.04 (0.96-1.12) | 1.03 (0.96-1.12) | 1.04 (0.96-1.12) | 1.34 (1.02-1.75) | 1.36 (1.04-1.77) | 1.36 (1.04-1.77) | 1.08 (0.77-1.5) | 1.05 (0.75-1.48) | 1.05 (0.75-1.47) |
| P | 0.378 | 0.389 | 0.382 | 0.034 | 0.027 | 0.027 | 0.664 | 0.761 | 0.775 |
| Rs4923457 |  |  |  |  |  |  |  |  |  |
| T | 1 | 1 | 1 | 1 | 1 | 1 | 1 | 1 | 1 |
| A | 1.01 (0.93-1.09) | 1.01 (0.93-1.09) | 1.01 (0.93-1.09) | 1.39 (1.07-1.82) | 1.36 (1.05-1.78) | 1.40 (1.07-1.83) | 1.12 (0.80-1.57) | 1.11 (0.79-1.55) | 1.12 (0.80-1.57) |
| P | 0.863 | 0.866 | 0.806 | 0.015 | 0.022 | 0.015 | 0.496 | 0.54 | 0.522 |
| Rs11030104 |  |  |  |  |  |  |  |  |  |
| G | 1 | 1 | 1 | 1 | 1 | 1 | 1 | 1 | 1 |
| A | 1.02 (0.94-1.10) | 1.02 (0.94-1.10) | 1.02 (0.94-1.10) | 1.34 (1.02-1.76) | 1.36 (1.03-1.79) | 1.36 (1.03-1.80) | 1.07 (0.77-1.50) | 1.05 (0.75-1.47) | 1.05 (0.75-1.47) |
| P | 0.609 | 0.623 | 0.615 | 0.036 | 0.028 | 0.028 | 0.675 | 0.765 | 0.782 |
| Rs6484320 |  |  |  |  |  |  |  |  |  |
| T | 1 | 1 | 1 | 1 | 1 | 1 | 1 | 1 | 1 |
| A | 1.02 (0.94-1.10) | 1.02 (0.94-1.10) | 1.02 (0.94-1.10) | 1.26 (0.97-1.65) | 1.27 (0.97-1.67) | 1.27 (0.97-1.67) | 1.07 (0.77-1.5) | 1.06 (0.75-1.48) | 1.05 (0.75-1.48) |
| P | 0.686 | 0.697 | 0.671 | 0.087 | 0.077 | 0.079 | 0.685 | 0.753 | 0.758 |
| Model A: Crude model Model B: Adjusted for age, sex Model C: Adjusted for age, sex, education, income level and physical activity | | | | | | | | | |

**Table S23 Odds ratios (95%CI) of central obesity for different SNPs in different smoke exposure groups (additive model)**

|  | Never smokers (n=12,852) | | | Former smokers (n=1,233) | | | Current smokers (n=987) | | |
| --- | --- | --- | --- | --- | --- | --- | --- | --- | --- |
|  | Model A | Model B | Model C | Model A | Model B | Model C | Model A | Model B | Model C |
| Rs6265 |  |  |  |  |  |  |  |  |  |
| A | 1 | 1 | 1 | 1 | 1 | 1 | 1 | 1 | 1 |
| G | 1.08 (1.03-1.13) | 1.08 (1.02-1.13) | 1.08 (1.02-1.13) | 1.37 (1.16-1.61) | 1.43 (1.21-1.70) | 1.44 (1.21-1.71) | 0.97 (0.80-1.18) | 0.94 (0.77-1.15) | 0.94 (0.77-1.14) |
| P | 0.002 | 0.005 | 0.004 | <0.001 | <0.001 | <0.001 | 0.774 | 0.547 | 0.524 |
| Rs4923457 |  |  |  |  |  |  |  |  |  |
| T | 1 | 1 | 1 | 1 | 1 | 1 | 1 | 1 | 1 |
| A | 1.04 (0.99-1.09) | 1.03 (0.98-1.09) | 1.04 (0.99-1.09) | 1.24 (1.06-1.46) | 1.30 (1.10-1.54) | 1.30 (1.10-1.54) | 1.03 (0.85-1.25) | 1.03 (0.84-1.25) | 1.02 (0.84-1.25) |
| P | 0.124 | 0.185 | 0.137 | 0.008 | 0.002 | 0.002 | 0.742 | 0.793 | 0.814 |
| Rs11030104 |  |  |  |  |  |  |  |  |  |
| G | 1 | 1 | 1 | 1 | 1 | 1 | 1 | 1 | 1 |
| A | 1.08 (1.02-1.13) | 1.07 (1.02-1.12) | 1.07 (1.02-1.13) | 1.35 (1.14-1.60) | 1.40 (1.18-1.67) | 1.41 (1.18-1.68) | 1.00 (0.82-1.21) | 0.98 (0.80-1.19) | 0.98 (0.80-1.19) |
| P | 0.004 | 0.009 | 0.008 | <0.001 | <0.001 | <0.001 | 0.984 | 0.838 | 0.815 |
| Rs6484320 |  |  |  |  |  |  |  |  |  |
| T | 1 | 1 | 1 | 1 | 1 | 1 | 1 | 1 | 1 |
| A | 1.06 (1.01-1.12) | 1.06 (1.01-1.11) | 1.06 (1.01-1.12) | 1.29 (1.09-1.51) | 1.32 (1.11-1.57) | 1.32 (1.12-1.57) | 0.94 (0.77-1.14) | 0.92 (0.76-1.12) | 0.92 (0.75-1.12) |
| P | 0.013 | 0.024 | 0.019 | 0.002 | 0.001 | 0.001 | 0.518 | 0.418 | 0.397 |
| Model A: Crude model Model B: Adjusted for age, sex Model C: Adjusted for age, sex, education, income level and physical activity | | | | | | | | | |

**S24 The new 8 SNPs both related with smoking behavior and BMI in EBI GWAS Catalog**

| SNP | P | Gene |
| --- | --- | --- |
| rs12902602 | 6 x 10^-13^ | CHRNB4, AC022748.2 |
| rs10182181 | 1 x 10^-18^ | DNAJC27, ADCY3 |
| rs3888190 | 6 x 10^-18^ | ATP2A1, SH2B1 |
| rs2112347 | 2 x 10^-12^ | SLC25A5P9, POC5 |
| rs205262 | 3 x 10^-9^ | ILRUN |
| rs13078960 | 3 x 10^-9^ | CADM2 |
| rs9641123 | 2 x 10^-11^ | CALCR |
| rs29941 | 2 x 10^-8^ | AC008556.1, KCTD15 |
