# Supplementary figures and images for: Brain-derived neurotrophic factor gene variants and obesity in former smokers
Source: BMC Genomics. 2021 Sep 15;22:668. doi: 10.1186/s12864-021-07928-0 (PMC8442367; doi:10.1186/s12864-021-07928-0)

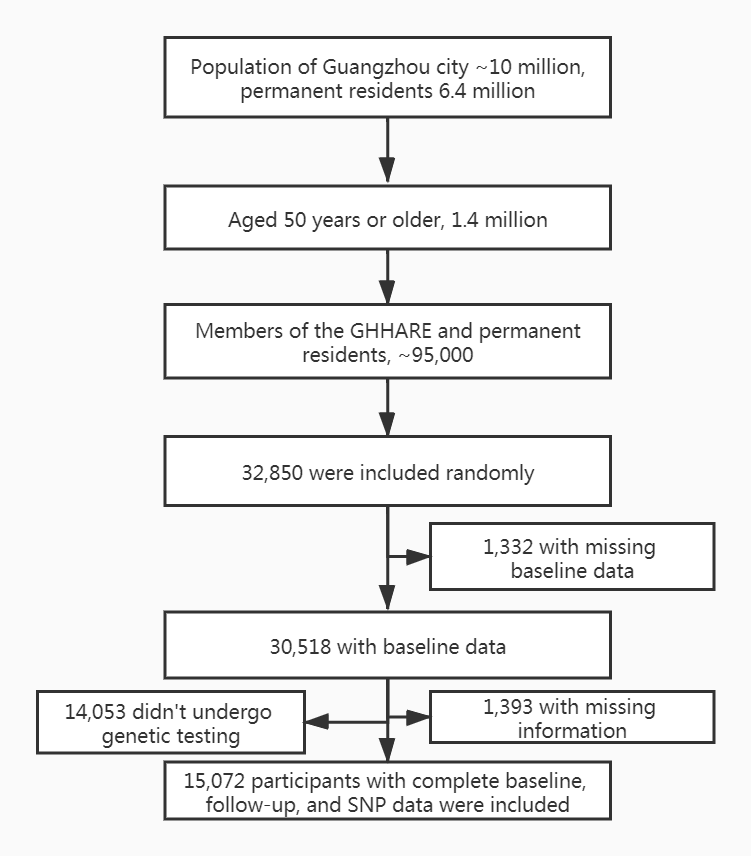

Supplement: Supplementary file 2 — Additional file 2: Fig. S1 The flow chart of participants. [file 12864_2021_7928_MOESM2_ESM.jpg]
